# Mediation Analyses Link Cardiometabolic Factors and Liver Fat With White Matter Hyperintensities and Cognitive Performance: A UK Biobank Study

**DOI:** 10.1016/j.bpsgos.2025.100488

**Published:** 2025-03-20

**Authors:** Daniel E. Askeland-Gjerde, Lars T. Westlye, Patrik Andersson, Max Korbmacher, Ann-Marie de Lange, Dennis van der Meer, Olav B. Smeland, Sigrun Halvorsen, Ole A. Andreassen, Tiril P. Gurholt

**Affiliations:** a Section for Precision Psychiatry, Division of Mental Health and Addiction, Oslo University Hospital, Oslo, Norway; b Institute of Clinical Medicine, University of Oslo, Oslo, Norway; c Department of Psychology, University of Oslo, Oslo, Norway; d K.G. Jebsen Centre for Neurodevelopmental Disorders, University of Oslo, Oslo, Norway; e AMRA Medical AB, Linköping, Sweden; f Neuro-SysMed Center of Excellence for Clinical Research in Neurological Diseases, Department of Neurology, Haukeland University Hospital, Bergen, Norway; g Mohn Medical Imaging and Visualization Centre, Department of Radiology, Haukeland University Hospital, Bergen, Norway; h Department of Health and Functioning, Western Norway University of Applied Sciences, Bergen, Norway; i Department of Clinical Neurosciences, Lausanne University Hospital and University of Lausanne, Lausanne, Switzerland; j Department of Psychiatry, University of Oxford, Oxford, United Kingdom; k School of Mental Health and Neuroscience, Faculty of Health, Medicine and Life Sciences, Maastricht University, Maastricht, the Netherlands; l Department of Cardiology, Oslo University Hospital and University of Oslo, Oslo, Norway

**Keywords:** Cerebral small vessel disease, Metabolic dysfunction–associated steatotic liver disease, Neurodegenerative diseases, Nonalcoholic fatty liver disease, Vascular cognitive impairment and dementia

## Abstract

**Background:**

Liver fat is associated with cardiometabolic disease, cerebrovascular disease, and dementia. Cerebrovascular disease, most often cerebral small vessel disease, identified by magnetic resonance imaging as white matter hyperintensities (WMHs) often contributes to dementia. However, liver fat’s role in the relationship between cardiometabolic risk, WMHs, and cognitive performance is unclear.

**Methods:**

In the UK Biobank cohort (*N* = 32,461, 52.6% female; mean age 64.2 ± 7.7 years; *n* = 23,354 in the cognitive performance subsample), we used linear regression to investigate associations between cardiometabolic factors measured at baseline and liver fat, WMHs, and cognitive performance measured at follow-up, which was 9.3 ± 2.0 years later on average. We used structural equation modeling to investigate whether liver fat mediated associations between cardiometabolic factors and WMHs and whether WMHs mediated associations between liver fat and cognitive performance.

**Results:**

Nearly all cardiometabolic factors were significantly associated with liver fat (|*r*| range = 0.03–0.41, *p* = 3.4 × 10^−8^ to 0) and WMHs (|*r*| = 0.04–0.15, *p* = 5.8 × 10^−13^ to 7.0 × 10^−159^) in regression models. Liver fat was associated with WMHs (*r* = 0.11, *p* = 4.3 × 10^−82^) and cognitive performance (*r* = −0.03, *p* = 1.6 × 10^−7^). Liver fat mediated the associations between cardiometabolic factors and WMHs (|β_mediation_| = 0.003–0.027, *p*_mediation_ = 1.9 × 10^−8^ to 0), and WMHs mediated the associations between liver fat and cognitive performance (β_mediation_ = −0.01, *p*_mediation_ = 0).

**Conclusions:**

Our findings indicate that liver fat mediates associations between cardiometabolic factors and WMHs and that WMHs mediate the association between liver fat and cognitive performance. This suggests that liver fat may be important for understanding the effects of cardiometabolic factors on cerebrovascular disease and cognitive function. Experimental studies are warranted to determine relevant targets for preventing vascular-driven cognitive impairment.

## Plain Language Summary

The study examined links between fat stored in the liver, small brain lesions, and cognitive skills, such as memory. The results indicated that people with more liver fat had more small lesions in the brain and that they did not perform as well on cognitive tests as people with less fat in the liver. Therefore, reducing fat in the liver may be beneficial to avoid cognitive decline resulting from small brain lesions.

Metabolic dysfunction–associated steatotic liver disease (MASLD), formerly called nonalcoholic fatty liver disease (NAFLD) (1), affects 30% of adults (2) and is associated with cerebrovascular disease (3) and dementia (4). Vascular pathology, most frequently cerebral small vessel disease (CSVD), is implicated in 50% to 70% of dementia cases (5,6). Because MASLD (2) and dementia (7) cases are expected to increase, it is vital to understand liver fat’s role in the early stages of CSVD-driven cognitive impairment both to identify at-risk individuals and to establish efficient preventive measures and treatment strategies.

MASLD is characterized by excessive fat accumulation in liver cells and one or more cardiometabolic abnormalities (8). Because the diagnoses of MASLD and NAFLD are highly concordant (9), we use the term MASLD for both diagnoses. MASLD is associated with CSVD (10, 11, 12), identified as white matter hyperintensities (WMHs) on magnetic resonance imaging (MRI) (5), although inconsistently (13,14). However, technological advances allow for accurate MRI liver fat quantification in large samples that may clarify the relationship between liver fat and CSVD (15).

Cardiometabolic factors have been associated with MASLD (8) and WMHs (5). However, whether the putative association between MASLD and WMHs partially explains associations between cardiometabolic factors and WMHs remains unclear. Because WMHs are associated with cognitive impairment (16), an association between MASLD and WMHs may partially explain an observed association between MASLD and cognitive performance (17). Furthermore, these associations may differ in males and females because sex differences have been shown for MASLD (18), CSVD (19), and dementia (20). We hypothesized that 1) cardiometabolic risk factors are associated with WMHs and that liver fat would mediate these associations; 2) liver fat is associated with cognitive performance and WMHs would mediate this association; and 3) there are sex-related differences in these associations.

## Methods and Materials

### Participant Sample

This observational study used UK Biobank data (access no. 27412) and was approved by the Norwegian Regional Committees for Medical and Health Research Ethics. All participants gave informed consent and could withdraw their consent at any time (opt-out list dated April 26, 2023).

Initially, we included eligible participants with liver and brain MRI (*n* = 41,760) (Figure S1). Secondly, we excluded participants with a history of chronic liver disease (except MASLD); alcohol-related disorders; malignancies of the liver, biliary tract, or central nervous system; encephalitis; myelitis; stroke; traumatic brain injury; and neurodegenerative and demyelinating disorders (*n* = 869) (Table S1). Thirdly, we excluded participants who lacked data on sex, education, anthropometric measurements, blood pressure, serum measures, smoking status, or alcohol consumption (*n* = 8430). After exclusions, the final sample included 32,461 participants; 23,354 participants completed cognitive testing at follow-up (i.e., cognitive subsample).

### Liver and Brain MRI

Liver and brain MRI were performed at 4 sites (Cheadle, Newcastle, Reading, and Bristol) at the follow-up assessment using Siemens 1.5T MAGNETOM Aera and 3T Skyra scanners, respectively (21,22). AMRA Researcher (AMRA Medical AB) was used to estimate liver fat percentage (15), and the University of Oxford’s Wellcome Centre for Integrative Neuroimaging estimated WMH volume using FSL BIANCA (23,24) and intracranial volume (ICV) with FreeSurfer (25).

### Demographic and Clinical Data

From the baseline assessment (Table S2), we included sex, education, ethnicity, body mass index (BMI), waist circumference, systolic blood pressure (SBP), diastolic blood pressure (DBP), alcohol consumption, and nonfasting high-sensitivity C-reactive protein (CRP), glycated hemoglobin (HbA1c), high-density lipoprotein (HDL) cholesterol, low-density lipoprotein (LDL) cholesterol, total cholesterol, and triglycerides. From the follow-up assessment, we included age, site, alcohol consumption, smoking status, liver fat, WMH, ICV, numeric memory, fluid intelligence, Trail Making Test B, matrix test, symbol digit substitution, tower rearranging, paired associate learning, and pairs matching.

Sex was gathered from National Health Service registers. We categorized education into higher (college or university degree), intermediate (A-levels, O-levels, or equivalent), and lower education (otherwise) and smoking status as current, former, and never. We calculated pulse pressure by subtracting DBP from SBP and alcohol consumption by converting total weekly and monthly alcohol consumption into grams of alcohol per week.

We classified participants with probable hypertension (blood pressure ≥ 140/90 mm Hg, antihypertensive treatment, or ICD-10 diagnoses I10–I15), diabetes (HbA1c ≥ 48 mmol/mol, antidiabetic treatment, or ICD-10 E10–E14 diagnoses), or dyslipidemia [HDL cholesterol < 1.03 mmol/L, LDL cholesterol > 4.13 mmol/L, total cholesterol ≥ 6.20 mmol/L, triglycerides > 2.25 mmol/L, lipid-lowering treatment, or ICD-10 diagnosis E78 (26,27)] using baseline information.

We classified participants with probable steatotic liver disease based on current or former diagnostic criteria (MASLD, NAFLD, metabolic dysfunction–associated fatty liver disease [MAFLD]) as follows. NAFLD was defined by liver fat ≥ 5% and baseline and follow-up alcohol consumption < 20/30 g/day (female/male) (28). MASLD was defined by probable NAFLD and ≥1 of the following: BMI ≥ 25 or ≥ 23 (Asian), waist circumference ≥ 80/94 cm (female/male) or waist circumference ≥ 80/90 cm (Asian, female/male), blood pressure ≥ 130/85 mm Hg or antihypertensive treatment, HbA1c ≥ 39 mmol/mol or diabetes, triglycerides ≥ 1.7 mmol/L or lipid-lowering treatment, and HDL cholesterol ≤ 1.3/1.0 mmol/L (female/male) or lipid-lowering treatment (8). MAFLD was defined by liver fat ≥ 5% and diabetes, BMI ≥ 25 or ≥ 23 (Asian) or ≥2 of the following: waist circumference ≥ 88/102 cm (female/male) or waist circumference ≥ 80/90 cm (Asian, female/male), blood pressure ≥ 130/85 mm Hg or antihypertensive treatment, HbA1c ≥ 39 mmol/mol, triglycerides ≥ 1.7 mmol/L or lipid-lowering treatment, HDL cholesterol < 1.3/1.0 mmol/L (female/male) or lipid-lowering treatment, and CRP > 2 mg/L (29).

### Cardiometabolic Principal Component Analysis

We conducted principal component analysis (PCA) across 11 correlated cardiometabolic variables (Figure S2) to create uncorrelated composite measures of cardiometabolic risk. To stabilize variances (30), we log-transformed CRP and triglycerides (due to non-normal distributions) (Figures S3 and S4) and standardized the remaining variables (i.e., BMI, waist circumference, SBP, DBP, pulse pressure, HbA1c, HDL cholesterol, LDL cholesterol, and total cholesterol) by mean centering and dividing by the standard deviation. Because PCA is sensitive to outliers (30), we removed values that deviated >3 SDs from the mean. A total of 2,362 participants lacked ≥1 data point after outlier removal (Table S4), but all participants had >50% cardiometabolic data and were included in subsequent analyses. Next, we imputed the missing cardiometabolic data based on the existing cardiometabolic data with a PCA method (*missMDA* R package) (31), conducted the PCA (*prcomp* R function), and extracted the resulting PCs 1 to 3 based on their explained variances, which were 32.0%, 23.4%, and 17.9%, respectively (Figure S5A). PC1’s largest loadings were from SBP, DBP, and anthropometric measures (loadings = 0.38–0.47) (Figure S5B); PC2’s largest loadings were from cholesterol and anthropometric measures (|loadings| = 0.20–0.64); and PC3’s largest loadings were from pulse pressure, SBP, and anthropometric measures (|loadings| = 0.35–0.50). All PC1’s loadings indicated higher cardiometabolic risk.

We also conducted sensitivity analysis using a similar PCA on the participants without incomplete cardiometabolic data and obtained results similar to those of the original PCA (Figure S6).

### Cognitive PCA

Because vascular cognitive impairment affects multiple cognitive domains (6), we derived a measure of general cognitive performance using PCA (Figure S7). We log-transformed Trail Making Test B and pairs matching scores (due to non-normal distributions) (Figures S8 and S9) and standardized the remaining variables (i.e., numeric memory, fluid intelligence, matrix test, symbol digit substitution, tower rearranging, and paired associate learning). Higher scores indicated better performance, except for Trail Making Test B and pairs matching. We did not include reaction time (data field 20023) because it may have a lower correlation with general cognitive performance (32) and a different genetic basis (33). We also did not include prospective memory (data field 20018) because it was a binary variable.

We used similar methods as described above. After outlier removal (Table S5), 9107 participants lacked >50% of cognitive data and were excluded from subsequent analyses. We imputed the missing cognitive data with existing cognitive test data for the remaining 23,354 participants, performed PCA, and extracted the resulting PC1 based on its explained variance (40.7%) (Figure S10A). All cognitive tests contributed to PC1, with the largest contributions coming from fluid intelligence, matrix test, and tower rearranging (loadings = 0.39–0.46) (Figure S10B). All loadings indicated better cognitive task performance, suggesting that the PC1 (hereafter referred to as general cognitive performance) captures general cognitive performance.

Additionally, we conducted sensitivity analysis using a similar PCA on the participants without incomplete cognitive data and obtained results similar to those of the original PCA (Figure S11).

### Statistical Analysis

We used R version 4.2.0 (34) for the analyses. Continuous variables were assessed using histograms and quantile-quantile plots (Figures S3, S4, S8, and S9). Because residuals from linear regression analyses with liver fat, WMH, Trail Making Test B, and pairs matching as outcomes and CRP and triglycerides as predictors were non-normal, we log-transformed these variables. The remaining continuous variables were standardized by mean centering and dividing by the standard deviation. We used the total sample (*N* = 32,461) for the analyses with liver fat and WMH as outcomes and the cognitive subsample (*n* = 23,354) for analyses with cognitive outcome measures. We tested for differences between males and females and between the total sample and the cognitive subsample using *t* tests and χ^2^ tests, as appropriate (Tables S6 and S7).

First, we conducted multiple linear regression analyses with liver fat, WMH, and general cognitive performance as outcomes to verify the assumptions of our planned mediation analyses. We used the cardiometabolic risk factors and PC1 to PC3 as predictors for all outcomes, liver fat and steatotic liver disease as predictors for WMH and general cognitive performance, and WMH as a predictor for general cognitive performance. Each predictor was analyzed by itself in a separate model. Next, we performed analyses with an interaction term between the predictor and sex to test for sex-related differences. We conducted sensitivity analyses with PC1 to PC3 derived without imputed data as predictors and for general cognitive performance derived without imputed data and individual cognitive tests as the outcomes.

We adjusted for linear (age) and nonlinear (age^2^) aging effects (35, 36, 37); sex (18,20,38); interactions between age, age^2^, and sex (age × sex and age^2^ × sex) (39); alcohol consumption (8,40); and smoking status (40,41) because they are associated with cardiometabolic, liver, and brain outcomes. We used follow-up alcohol consumption because it was correlated with baseline values (*r* = 0.78). We adjusted for scanner site because scanner differences may influence imaging results; ICV in WMH analyses because brain volume may influence lesion volume; and education in cognitive analyses to mitigate confounding from early-life cognitive performance. We also performed sensitivity analyses adjusted for hypertension, diabetes, and dyslipidemia status.

**Figure 1:**
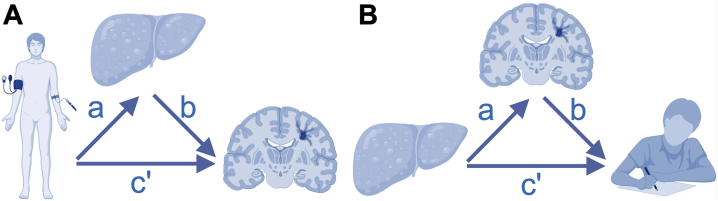
Path diagram of the mediation analyses. The figure shows path diagrams of the mediation analyses with **(A)** white matter hyperintensities (WMHs) as outcome, liver fat as mediator, and cardiometabolic factors as predictors and with **(B)** general cognitive performance as outcome, WMHs as mediator, and liver fat and steatotic liver disease as predictors. (Figure created with BioRender.com.)

Next, we performed structural equation modeling (SEM) mediation analyses (Figure 1) using the *sem* function in the *lavaan* R package version 0.6-11 (42). We computed standard errors with bootstrapping using 10,000 draws. Each model consists of 2 regression equations and includes outcome (*Y*), mediator (*M*), predictor (*X*), intercepts (*i*), and error terms (*e*). *Lavaan* does not support interaction terms (42). Therefore, we performed additional analyses in males and females separately for predictors that had significant interactions with sex in the regression analyses.

In the first set of mediation analyses, we used WMH as outcome, liver fat as mediator, cardiometabolic factors and PC1 to PC3 separately as predictors (Figure 1A) as follows:

(1) $$M_{Liver\;fat}\;=\;i_{M}\;+\;aX_{Cardiometabolic\;factor}\;+\;\text{age}\;+\;{\text{age}}^{2}\;+\;\text{sex}\;+\;\text{site}\;+\;\text{smoking}\;\text{status}+\;\text{alcohol}\;\text{consumption}\;+\;eM$$

(2) $$Y_{WMH}\;=\;i_{Y}\;+\;c^{'}X_{Cardiometabolic\;factor}\;+\;bM_{Liver\;fat}\;+\;\text{age}\;+\;{\text{age}}^{2}\;+\;\text{sex}\;+\;\text{site}\;+\;\text{smoking}\;\text{status}+\;\text{alcohol}\;\text{consumption}\;+\;eY$$

Initially, we included ICV as a covariate in equation 2. The confirmatory fit index (CFI), standardized root mean squared residual (SRMR), and root mean square error of approximation (RMSEA) were acceptable, but the Tucker-Lewis index (TLI) was poor, possibly due to the number of model parameters, low degrees of freedom, and low correlation between ICV and remaining variables (43). After removing ICV from the equation, the models were saturated. We conducted sensitivity analyses with PC1 to PC3 derived without imputed data and analyses adjusted for hypertension, diabetes, and dyslipidemia. These models were also saturated.

In the second set of mediation analyses, we used general cognitive performance as outcome, WMH as mediator, and liver fat and steatotic liver disease separately as predictors (Figure 1B) as follows:

(3) $$M_{WMH}\;=\;i_{M}\;+\;aX_{Liver\;fat}\;+\;\text{age}\;+\;{\text{age}}^{2}\;+\;\text{sex}\;+\;\text{site}\;+\;\text{smoking}\;\text{status}\;+\;\text{alcohol}\;\text{consumption}\;+\;\text{ICV}\;+\;eM$$

(4) $$Y_{General\;cognitive\;performance}\;=\;i_{Y}\;+\;c^{'}X_{Liver\;fat}\;+\;bM_{WMH}\;+\;\text{age}\;+\;{\text{age}}^{2}+\;\text{sex }+\text{site }+\;\text{smoking}\;\text{status }+\;\text{alcohol}\;\text{consumption}\;+\;\text{ICV }+\;\text{education}\;+\;eY$$

The fit measures were good: CFI = 0.999, TLI = 0.985–0.990, RMSEA = 0.018–0.022, and SRMR = 0.003. We conducted sensitivity mediation analyses on general cognitive performance derived without imputed data (CFI = 0.999, TLI = 0.987–0.991, RMSEA = 0.017–0.021, and SRMR = 0.003), analyses adjusted for hypertension, diabetes, and dyslipidemia (CFI = 0.999, TLI = 0.987–0.991, RMSEA = 0.016–0.019, and SRMR = 0.002), and on individual cognitive tests (CFI = 0.998–1.000, TLI = 0.975–0.998, RMSEA = 0.007–0.025, and SRMR = 0.002–0.004).

We report partial correlation coefficients (*r*s) (44) from linear regression analyses and standardized regression coefficients (βs) from mediation analyses. We derived a study-wide Bonferroni threshold *p* ≤ .05/N = .05/156 = .0003, where N is the analysis count. We planned 28 (14 sex-specific), 36 (18 sex-specific), and 38 (19 sex-specific) regression analyses with liver fat, WMH, and general cognitive performance as outcomes, respectively, and 42 (28 sex-specific) and 12 (8 sex-specific) mediation analyses with WMH and general cognitive performance as outcomes, respectively. Sensitivity analyses were not included in the analysis count. Results are described as significant if they passed the Bonferroni threshold. Because R uses double-precision values, some *p* values are reported as 0, which indicates approximately equal to 0.

## Results

### Sample Description

The sample consisted of 32,461 UK Biobank participants (*n* = 17,063 [52.6%] females) (Table S5), who were mostly middle-age or older (mean age, 64.2 ± 7.7 years; range, 48–83), had completed higher (46.5%) or intermediate (32.8%) education, and were followed up 9.4 ± 2.0 years (range 4.3–14.9) later on average. Males had higher BMI (27.08 ± 3.64 vs. 26.03 ± 4.52) and liver fat (4.70 ± 4.18 vs. 3.87 ± 3.99) on average and higher risk of hypertension (54.3% vs. 36.7%), diabetes (4.6% vs. 2.3%), and dyslipidemia (61.9% vs. 46.8%) than females. Steatotic liver disease prevalences varied based on the diagnostic criteria used, but males had consistently higher risk of steatotic liver diseases, NAFLD (16.9% vs. 14.1%), MASLD (16.5% vs. 13.7%), and MAFLD (26.8% vs. 17.0%), than females. The cognitive subsample (*n* = 23,354) had demographic, clinical, and imaging data comparable to that of the total sample (Table S6).

### Cardiometabolic Factors and Liver Fat

**Figure 2:**
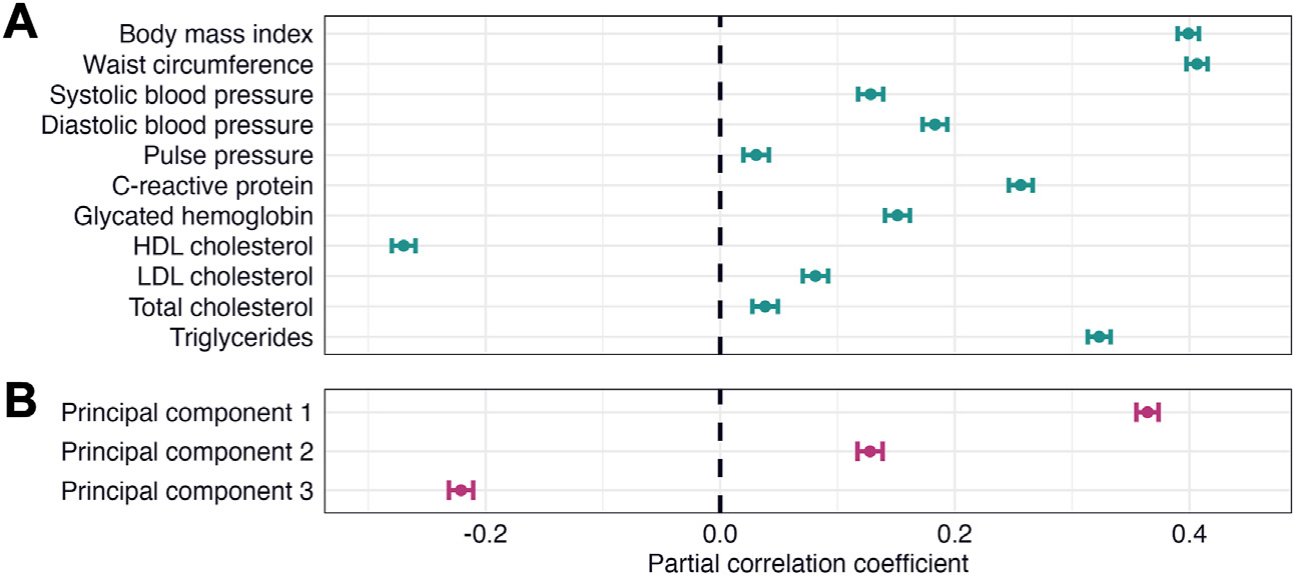
Linear associations between cardiometabolic factors and liver fat. The figure shows forest plots with the associations of **(A)** cardiometabolic risk factors and **(B)** cardiometabolic principal components with liver fat. The error bars correspond to 95% CIs. The regression models were adjusted for age, age^2^, sex, age × sex, age^2^ × sex, site, smoking status, and alcohol consumption. HDL, high-density lipoprotein; LDL, low-density lipoprotein.

Multiple linear regression analyses revealed significant associations between all cardiometabolic factors and liver fat (Figure 2A, B and Table S8). BMI, waist circumference, triglycerides, and PC1 (largest loadings from anthropometric and blood pressure measurements) showed medium to large effects (*r* = 0.323–0.407, *p* values = 0). The remaining variables showed small to medium effects (|*r*| = 0.031–0.270, *p* values = 3.39 × 10^−8^ to 2.95 × 10^−243^). All variables except HDL cholesterol and PC3 (negative loadings from BMI and waist circumference) were associated with higher liver fat.

Multiple linear regression analyses revealed significant interactions between sex and BMI, DBP, CRP, LDL cholesterol, and triglycerides on liver fat (Table S9). An increase in BMI was associated with a steeper increase in liver fat in males than in females (*r* = 0.026, *p* = 2.21 × 10^−6^), while increases in DBP, CRP, LDL cholesterol, and triglycerides were associated with a steeper increase in liver fat in females than in males (*r* = −0.023 to −0.070, *p* values = 3.72 × 10^−5^ to 6.73 × 10^−37^).

Sensitivity analyses adjusted for hypertension, diabetes, and dyslipidemia yielded largely similar results (Tables S10 and S11).

### Cardiometabolic Factors, Liver Fat, and WMH

**Figure 3:**
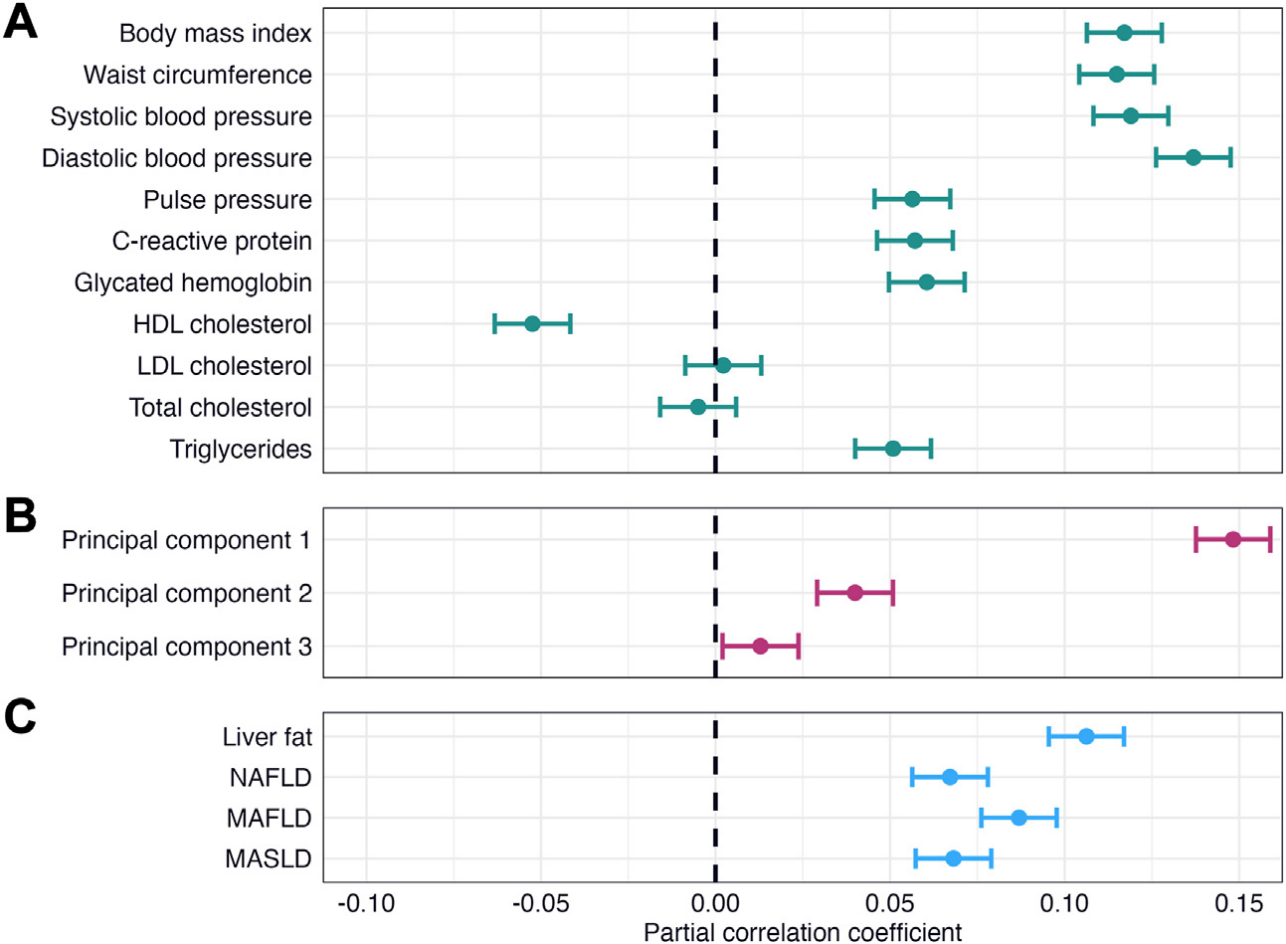
Linear associations between cardiometabolic factors, liver fat, and white matter hyperintensities (WMHs). The figure shows forest plots with the associations of **(A)** cardiometabolic risk factors, **(B)** cardiometabolic principal components, and **(C)** liver fat and steatotic liver disease with WMHs. The error bars correspond to 95% CIs. The regression models were adjusted for age, age^2^, sex, age × sex, age^2^ × sex, site, smoking status, alcohol consumption, and intracranial volume. HDL, high-density lipoprotein; LDL, low-density lipoprotein; MAFLD, metabolic dysfunction–associated fatty liver disease; MASLD, metabolic dysfunction–associated steatotic liver disease; NAFLD, nonalcoholic fatty liver disease.

Linear regression revealed significant associations between all cardiometabolic factors (except LDL cholesterol, total cholesterol, and PC3) and WMH (Figure 3A, B and Table S12) with small to medium effects (*r* = 0.040–0.148, *p* values = 5.85 × 10^−13^ to 6.95 × 10^−159^). BMI, waist circumference, SBP, DBP, and PC1 had the largest effects. Liver fat and steatotic liver diseases were associated with higher WMH volume with small effects (*r* = 0.067–0.106, *p* values = 8.78 × 10^−34^ to 4.34 × 10^−82^) (Figure 3C).

Linear regression analyses revealed significant interactions between sex and BMI, waist circumference, and PC2 on WMH (Table S13). An increase in BMI, waist circumference, and PC2 (largest loadings from BMI and waist circumference) was associated with a steeper increase in WMH in males than in females (*r* = 0.023–0.045, *p* values = 3.67 × 10^−5^ to 2.43 × 10^−16^).

Sensitivity analyses adjusted for hypertension, diabetes, and dyslipidemia yielded largely similar but attenuated results (Tables S14 and S15).

### Cardiometabolic Factors, Liver Fat, WMHs, and Cognitive Performance

**Figure 4:**
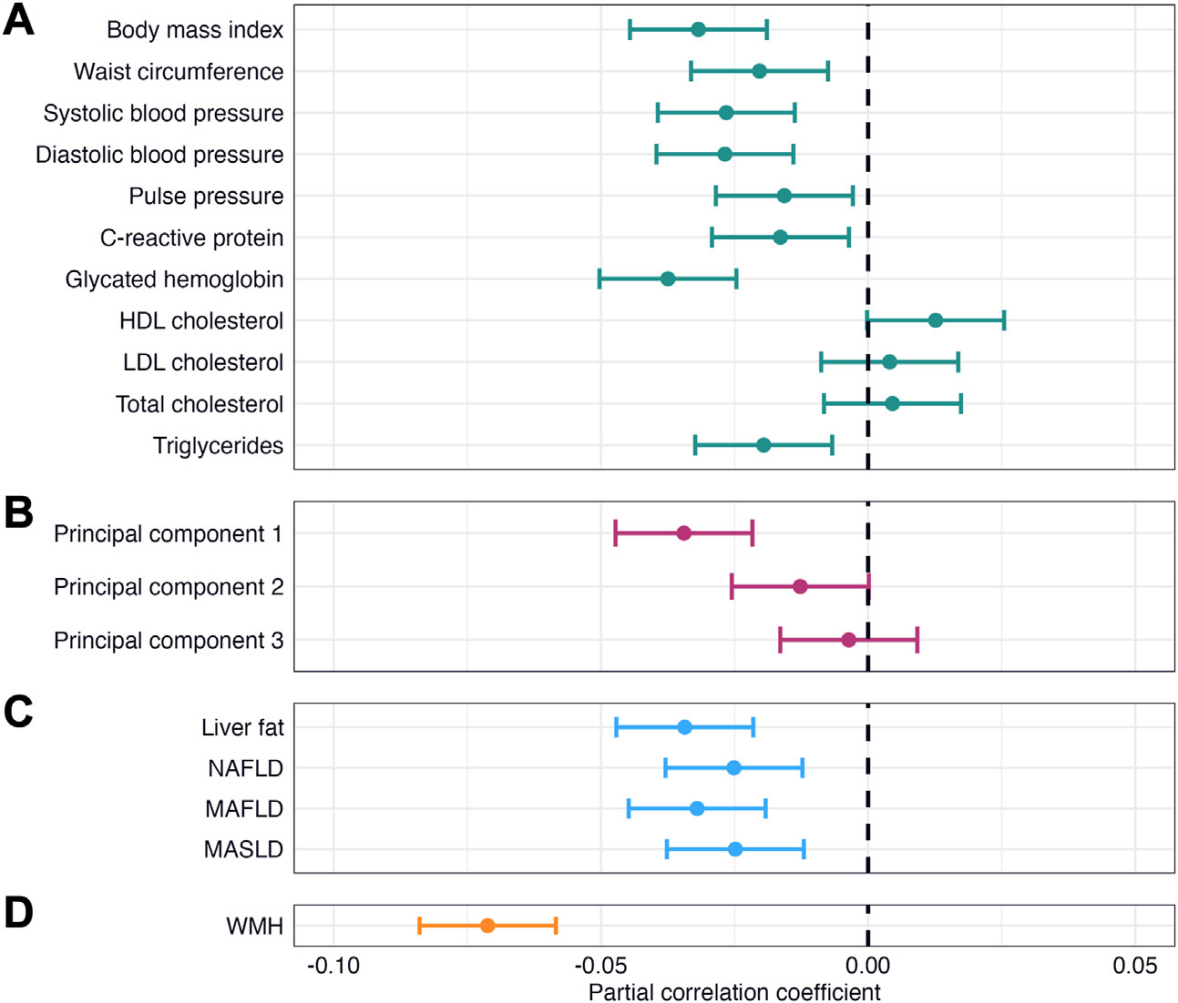
Linear associations between cardiometabolic factors, liver fat, white matter hyperintensities (WMHs), and general cognitive performance. The figure shows forest plots with the associations of **(A)** cardiometabolic risk factors, **(B)** cardiometabolic principal components, **(C)** liver fat and steatotic liver disease, and **(D)** WMHs with general cognitive performance. The error bars correspond to 95% CIs. The regression models were adjusted for age, age^2^, sex, age × sex, age^2^ × sex, site, smoking status, alcohol consumption, education, and intracranial volume (only WMHs). HDL, high-density lipoprotein; LDL, low-density lipoprotein; MAFLD, metabolic dysfunction–associated fatty liver disease; MASLD, metabolic dysfunction–associated steatotic liver disease; NAFLD, nonalcoholic fatty liver disease.

Linear regression revealed significant associations between general cognitive performance and BMI, SBP, DBP, HbA1c, and PC1 (*r* = −0.027 to −0.037, *p* values = 5.00 × 10^−5^ to 1.03 × 10^−8^) (Figure 4A, B and Table S16). Liver fat and steatotic liver diseases were significantly associated with general cognitive performance (*r* = −0.025 to −0.034, *p* values = 1.47 × 10^−4^ to 1.59 × 10^−7^) (Figure 4C). Of all the variables, WMH had the largest effect on general cognitive performance (*r* = −0.071, *p* = 1.27 × 10^−27^) (Figure 4D). None of the predictors had significant interactions with sex (Table S17).

Sensitivity analyses adjusted for hypertension, diabetes, and dyslipidemia and analyses on general cognitive performance derived without imputed cognitive data yielded similar but attenuated results (Tables S18–S21).

Follow-up analyses revealed significant associations with a range of individual cognitive tests, most often numeric memory, matrix test, and paired associate learning, but no significant interactions with sex (Tables S22 and S23). Liver fat was significantly associated with all cognitive tests except Trail Making Test B and pairs matching.

### Liver Fat Mediates the Associations Between Cardiometabolic Factors and WMH

**Figure 5:**
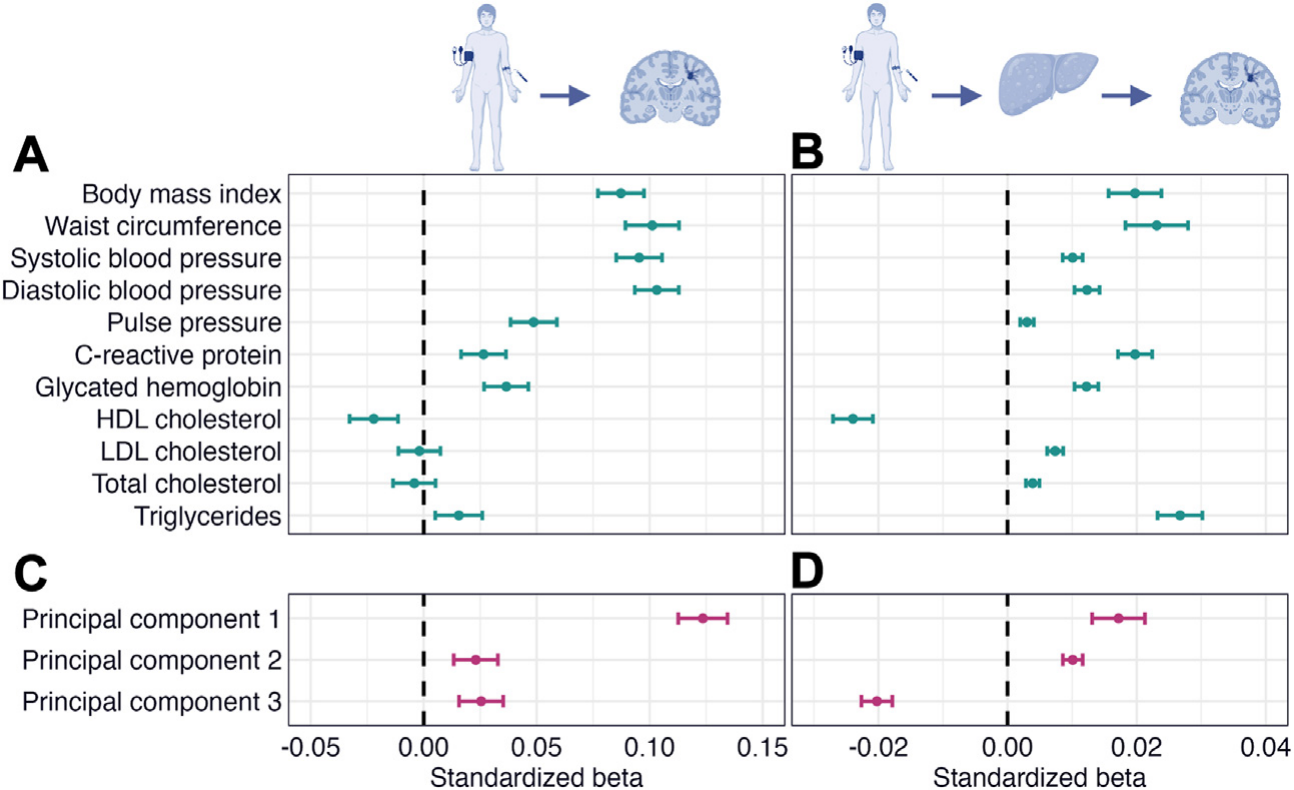
Mediation analyses with white matter hyperintensities (WMHs) as the outcome and liver fat as the mediator. The figure shows forest plots with the **(A)** direct and **(B)** mediation (i.e., indirect) effects via liver fat of cardiometabolic risk factors and the **(C)** direct and **(D)** mediation effects via liver fat of cardiometabolic principal components on WMHs. Error bars correspond to standardized 95% CIs. (Illustrations created with BioRender.com.) HDL, high-density lipoprotein; LDL, low-density lipoprotein.

SEM mediation analyses revealed significant total and mediation (i.e., indirect) effects via liver fat on WMHs for all cardiometabolic factors except LDL cholesterol, total cholesterol, and PC3 (Table S24). BMI, waist circumference, SBP, DBP, and PC1 had the largest direct effects (β = 0.087–0.124, *p* values = 0) (Figure 5A, C), while waist circumference, HDL cholesterol, and triglycerides had the largest mediation effects (|β| = 0.023–0.027, *p* values = 0) (Figure 5B, D).

Sensitivity analyses adjusted for hypertension, diabetes, and dyslipidemia revealed similar but attenuated results (Table S25).

### Liver Fat Mediates the Associations Between Cardiometabolic Factors and WMH: Sex-Related Differences

**Figure 6:**
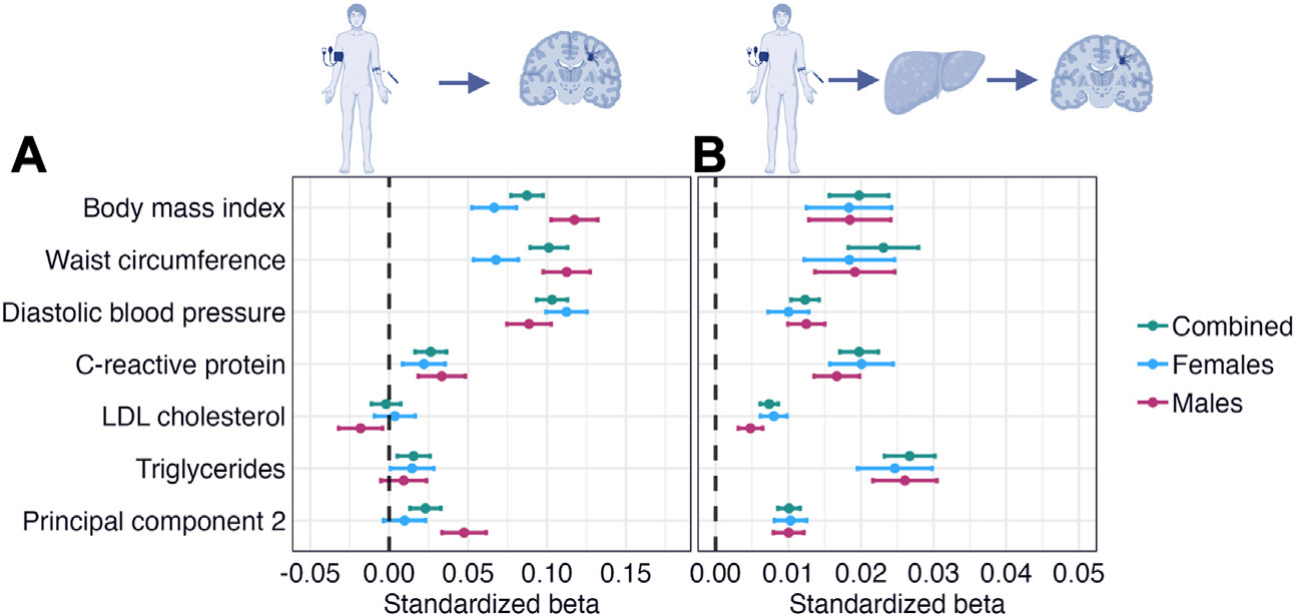
Mediation analyses with white matter hyperintensities (WMHs) as the outcome and liver fat as the mediator in the total sample and in males and females separately. The figure shows forest plots with the **(A)** direct and **(B)** mediation effects via liver fat of cardiometabolic risk factors and cardiometabolic principal components on WMHs. Error bars correspond to standardized 95% CIs. (Illustrations created with BioRender.com.) LDL, low-density lipoprotein.

Sex-stratified analyses (Table S26) revealed higher direct effects (Figure 6A) on WMH of BMI and waist circumference in males (β = 0.112–0.117, *p* values = 0) than in females (β = 0.067–0.068, *p* values = 0), while the mediation effects via liver fat (Figure 6B) were similar (β = 0.018–0.019, *p* values = 6.42 × 10^−9^ to 8.06 × 10^−12^). Furthermore, PC2 (largest loadings from anthropometric and cholesterol measures) had a significant total effect on WMH in males (β = 0.058, *p* = 4.44 × 10^−16^) but not in females (β = 0.020, *p* = 2.84 × 10^−3^). The results indicate that the direct effects of anthropometric measures on WMH may be stronger in males than females, while the mediation effects via liver fat are similar.

Sensitivity analyses adjusted for hypertension, diabetes, and dyslipidemia revealed similar but attenuated results (Table S27).

### WMHs Mediate the Associations of Liver Fat and Steatotic Liver Diseases With Cognitive Performance

**Figure 7:**
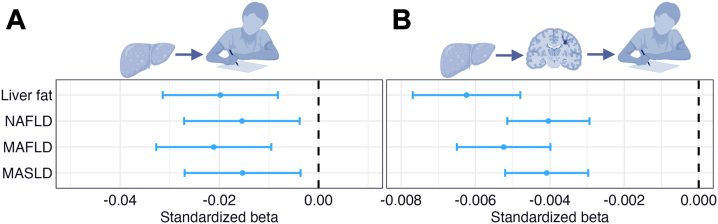
Mediation analyses with general cognitive performance as the outcome and white matter hyperintensities (WMHs) as the mediator. The figure shows forest plots with the **(A)** direct and **(B)** mediation effects via WMHs of liver fat and steatotic liver disease on general cognitive performance. Error bars correspond to standardized 95% CIs. (Illustrations created with BioRender.com.) MAFLD, metabolic dysfunction–associated fatty liver disease; MASLD, metabolic dysfunction–associated steatotic liver disease; NAFLD, nonalcoholic fatty liver disease.

SEM mediation analyses revealed significant total and mediation effects via WMH on general cognitive performance for both liver fat and steatotic liver diseases (Table S28). Effect sizes were comparable across predictors for direct effects (β = −0.015 to −0.021, *p* values = 0.010 to 3.60 × 10^−4^) (Figure 7A) and mediation effects (β = −0.004 to −0.006, *p* values = 7.56 × 10^−13^ to 0) (Figure 7B).

In follow-up analyses, WMH mediated the associations of numeric memory, symbol digits substitution, and paired associate learning with liver fat (Table S29). Sensitivity analyses on general cognitive performance derived without imputed data and analyses adjusted for hypertension, diabetes, and dyslipidemia yielded similar but attenuated results (Tables S30 and S31).

## Discussion

In this study, we showed that liver fat mediated the associations between cardiometabolic risk factors and higher WMH and that WMH mediated the associations between higher liver fat and lower cognitive performance in middle-age and older participants. Furthermore, higher BMI and waist circumference may be more strongly associated with liver fat and WMH in males than in females. Our results indicate that liver fat may contribute to CSVD and cognitive performance and that a greater WMH burden may link higher liver fat to lower cognitive performance. Therefore, liver fat may be a relevant treatment target for preventing the development of vascular cognitive impairment.

SEM mediation analyses revealed that liver fat mediated the link between nearly all cardiometabolic factors and WMH, thereby expanding our results from the corresponding regression analyses. Our findings are consistent with observed associations between WMH and anthropometric (45), blood pressure (46), and serum (47, 48, 49, 50, 51) measurements. The link between liver fat and WMH may be explained by liver fat’s association with exacerbated cardiometabolic risk (52, 53, 54, 55) and inflammatory factors such as homocysteine (56). While causal interpretations remain speculative, our results implicate liver fat in the link between cardiometabolic risk and higher WMH volume.

The cardiometabolic factors BMI, waist circumference, CRP, HDL cholesterol, triglycerides, and PC1 (largest loadings from anthropometric and blood pressure measures) had the overall largest mediation effects via liver fat on WMH. Our findings are consistent with the strong links between liver fat and anthropometric measures (57), the hypothesis that ectopic fat (e.g., liver fat) may be more strongly associated with cerebrovascular disease than subcutaneous fat (58), and observations that liver fat may initiate inflammatory pathways (59, 60, 61), alter lipid and lipoprotein regulation (55), and contribute to higher blood pressure through higher vasoconstriction (62) and impaired peripheral vasodilation (63). Taken together, our findings suggest that interventions aimed at general and abdominal obesity, dyslipidemia, and low-grade inflammation may be particularly beneficial in preventing liver fat accumulation and WMH development.

In the current sample, males had higher cardiometabolic risk, liver fat, and WMH volume on average than females. Regression analyses revealed steeper increases in liver fat and WMH per increase in BMI in males, consistent with former findings on MASLD (64,65) and CSVD (19). In mediation analyses, PC2 (largest loadings from anthropometric measures) was only significantly associated with WMH in males, and the direct effect sizes of BMI and waist circumference were larger in males than females, while indirect effects via liver fat were similar. Perhaps males store less fat in the subcutis than females (38), leading to a more harmful body fat distribution that could contribute to CSVD and possibly other brain outcomes, as has been shown for brain age (66,67).

We showed that WMH mediated the associations between lower general cognitive performance and liver fat and steatotic liver disease, expanding on previous observations (10, 11, 12,17). Furthermore, in analyses on individual cognitive tests, WMH mediated associations between liver fat and lower performance on numeric memory, symbol digit substitution, and paired associate learning tests, which are tests that cover working memory, processing speed, and verbal declarative memory (32). Thus, our findings may suggest a role of liver fat in the development of CSVD-driven cognitive decline. Improving cardiometabolic health is one of the strategies that has been outlined to prevent dementia cases (68), and our findings suggest that lowering liver fat may also be relevant, as has been shown for muscle fat infiltration (69). Importantly, our study demonstrated close links between general cardiometabolic risk and liver fat. Weight loss interventions can be effective in lowering liver fat (70), but that does not always improve MASLD, especially in severe cases (71,72). Therefore, interventions for preventing liver fat accumulation in the general population are needed.

Our study has strengths and limitations. It is significantly larger than previous studies and assesses liver fat (15) and WMH (24) with accurate, quantitative methods. We used a well-characterized sample, individual and composite cardiometabolic factors, liver fat percentage and steatotic liver disease diagnoses, and general cognitive performance and individual cognitive tests, and we tested for sex differences. The blood samples were nonfasting, which might have influenced the findings, although the measurements used in this study are largely unaffected by fasting (73,74), except for triglycerides (74). However, nonfasting triglycerides are still considered valid (74). We only assessed WMH because it was beyond the scope of this study to investigate other CSVD markers. We created 2 mediation models in our study, but future studies may consider implementing serial mediation. UK Biobank participants are healthier, wealthier, and less ethnically diverse than the general U.K. population (75,76), which may limit the generalizability of our findings. Although brain MRI and cognitive testing were performed years after cardiometabolic assessment, we did not use cardiometabolic or imaging data from multiple time points. Therefore, we cannot fully exclude different directions of effects, altered associations due to changes during follow-up, learning effects of repeated cognitive testing, or the possibility that other confounders (e.g., lung function) might have influenced the results. Thus, differently designed studies are needed to make causal claims.

### Conclusions

Our findings suggest that liver fat may play a role in CSVD both directly and by mediating the associations between cardiometabolic risk factors and higher WMH volume. Higher BMI and waist circumference may be more strongly associated with liver fat and WMH in males than in females, while the link between liver fat and WMH appears to be similar in both sexes. Our results indicate links between liver fat and cognitive performance for general cognitive performance, working memory, processing speed, and verbal declarative memory. The associations with cognitive performance were mediated by higher WMH volume, which suggests that liver fat may contribute to the development of vascular cognitive impairment. Our findings warrant experimental studies on the underlying mechanisms and on liver fat as a potential target for preventing or delaying cognitive decline.

## Supplementary Material

Figures S1–S11 and Tables S1–S31

Key Resource Table

## References

[1] Rinella M.E., Lazarus J.V., Ratziu V., Francque S.M., Sanyal A.J., Kanwal F. (2023). A multisociety Delphi consensus statement on new fatty liver disease nomenclature. J Hepatol.

[2] Paik J.M., Henry L., Younossi Y., Ong J., Alqahtani S., Younossi Z.M. (2023). The burden of nonalcoholic fatty liver disease (NAFLD) is rapidly growing in every region of the world from 1990 to 2019. Hepatol Commun.

[3] Moon J.H., Jeong S., Jang H., Koo B.K., Kim W. (2023). Metabolic dysfunction-associated steatotic liver disease increases the risk of incident cardiovascular disease: A nationwide cohort study. EClinicalmedicine.

[4] Kim G.A., Oh C.H., Kim J.W., Jeong S.J., Oh I.H., Lee J.S. (2022). Association between non-alcoholic fatty liver disease and the risk of dementia: A nationwide cohort study. Liver Int.

[5] Wardlaw J.M., Smith C., Dichgans M. (2019). Small vessel disease: Mechanisms and clinical implications. Lancet Neurol.

[6] van der Flier W.M., Skoog I., Schneider J.A., Pantoni L., Mok V., Chen C.L.H., Scheltens P. (2018). Vascular cognitive impairment. Nat Rev Dis Primers.

[7] GBD 2019 Dementia Forecasting Collaborators (2022). Estimation of the global prevalence of dementia in 2019 and forecasted prevalence in 2050: An analysis for the Global Burden of Disease Study 2019. Lancet Public Health.

[8] European Association for the Study of the Liver (EASL), European Association for the Study of Diabetes (EASD), European Association for the Study of Obesity (EASO) (2024). EASL–EASD–EASO Clinical Practice Guidelines on the management of metabolic dysfunction-associated steatotic liver disease (MASLD). J Hepatol.

[9] Hagström H., Vessby J., Ekstedt M., Shang Y. (2024). 99% of patients with NAFLD meet MASLD criteria and natural history is therefore identical. J Hepatol.

[10] Lu Y., Pike J.R., Hoogeveen R., Walker K., Raffield L., Selvin E. (2024). Nonalcoholic fatty liver disease and longitudinal change in imaging and plasma biomarkers of Alzheimer disease and vascular pathology. Neurology.

[11] Jang H., Kang D., Chang Y., Kim Y., Lee J.S., Kim K.W. (2019). Non-alcoholic fatty liver disease and cerebral small vessel disease in Korean cognitively normal individuals. Sci Rep.

[12] McCracken C., Raisi-Estabragh Z., Veldsman M., Raman B., Dennis A., Husain M. (2022). Multi-organ imaging demonstrates the heart-brain-liver axis in UK Biobank participants. Nat Commun.

[13] Weinstein G., Zelber-Sagi S., Preis S.R., Beiser A.S., DeCarli C., Speliotes E.K. (2018). Association of nonalcoholic fatty liver disease with lower brain volume in healthy middle-aged adults in the Framingham study. JAMA Neurol.

[14] Weinstein G., O’Donnell A., Frenzel S., Xiao T., Yaqub A., Yilmaz P. (2024). Nonalcoholic fatty liver disease, liver fibrosis, and structural brain imaging: The Cross-Cohort Collaboration. Eur J Neurol.

[15] Linge J., Borga M., West J., Tuthill T., Miller M.R., Dumitriu A. (2018). Body composition profiling in the UK Biobank imaging study. Obesity (Silver Spring).

[16] Debette S., Schilling S., Duperron M.G., Larsson S.C., Markus H.S. (2019). Clinical significance of magnetic resonance imaging markers of vascular brain injury: A systematic review and meta-analysis. JAMA Neurol.

[17] Mikkelsen A.C.D., Kjærgaard K., Schapira A.H.V., Mookerjee R.P., Thomsen K.L. (2025). The liver-brain axis in metabolic dysfunction-associated steatotic liver disease. Lancet Gastroenterol Hepatol.

[18] Ji H., Cheng S., Heart-Liver Axis Research Collaboration (2024). Sex differences in prevalence and prognosis of steatotic liver disease phenotypes: Biological sex matters. J Hepatol.

[19] Jiménez-Sánchez L., Hamilton O.K.L., Clancy U., Backhouse E.V., Stewart C.R., Stringer M.S. (2021). Sex differences in cerebral small vessel disease: A systematic review and meta-analysis. Front Neurol, Internet.

[20] Gong J., Harris K., Lipnicki D.M., Castro-Costa E., Lima-Costa M.F., Diniz B.S. (2023). Sex differences in dementia risk and risk factors: Individual-participant data analysis using 21 cohorts across six continents from the COSMIC consortium. Alzheimers Dement.

[21] Alfaro-Almagro F., Jenkinson M., Bangerter N.K., Andersson J.L.R., Griffanti L., Douaud G. (2018). Image processing and Quality Control for the first 10,000 brain imaging datasets from UK Biobank. Neuroimage.

[22] Miller K.L., Alfaro-Almagro F., Bangerter N.K., Thomas D.L., Yacoub E., Xu J. (2016). Multimodal population brain imaging in the UK Biobank prospective epidemiological study. Nat Neurosci.

[23] Jenkinson M., Beckmann C.F., Behrens T.E.J., Woolrich M.W., Smith S.M. (2012). FSL. Neuroimage.

[24] Griffanti L., Zamboni G., Khan A., Li L., Bonifacio G., Sundaresan V. (2016). BIANCA (Brain Intensity AbNormality Classification Algorithm): A new tool for automated segmentation of white matter hyperintensities. Neuroimage.

[25] Fischl B.F.S. (2012). FreeSurfer. Neuroimage.

[26] Kopin L., Lowenstein C.J. (2017). Dyslipidemia. Ann Intern Med.

[27] Arvanitis M., Lowenstein C.J. (2023). Dyslipidemia. Ann Intern Med.

[28] European Association for the Study of the Liver (EASL), European Association for the Study of Diabetes (EASD), European Association for the Study of Obesity (EASO) (2016). EASL-EASD-EASO clinical practice guidelines for the management of non-alcoholic fatty liver disease. Obes Facts.

[29] Eslam M., Newsome P.N., Sarin S.K., Anstee Q.M., Targher G., Romero-Gomez M. (2020). A new definition for metabolic dysfunction-associated fatty liver disease: An international expert consensus statement. J Hepatol.

[30] Greenacre M., Groenen P.J.F., Hastie T., D’Enza A.I., Markos A., Tuzhilina E. (2022). Principal component analysis. Nat Rev Methods Primer.

[31] Josse J., Husson F. (2016). missMDA: A package for handling missing values in multivariate data analysis. J Stat Soft.

[32] Fawns-Ritchie C., Deary I.J. (2020). Reliability and validity of the UK Biobank cognitive tests. PLoS One.

[33] Davies G., Lam M., Harris S.E., Trampush J.W., Luciano M., Hill W.D. (2018). Study of 300,486 individuals identifies 148 independent genetic loci influencing general cognitive function. Nat Commun.

[34] R Core Team (2024). https://www.r-project.org/.

[35] Chia C.W., Egan J.M., Ferrucci L. (2018). Age-related changes in glucose metabolism, hyperglycemia, and cardiovascular risk. Circ Res.

[36] Debette S., Markus H.S. (2010). The clinical importance of white matter hyperintensities on brain magnetic resonance imaging: Systematic review and meta-analysis. BMJ.

[37] Gurholt T.P., Kaufmann T., Frei O., Alnæs D., Haukvik U.K., van der Meer D. (2021). Population-based body–brain mapping links brain morphology with anthropometrics and body composition. Transl Psychiatry.

[38] Gerdts E., Regitz-Zagrosek V. (2019). Sex differences in cardiometabolic disorders. Nat Med.

[39] Zhernakova D.V., Sinha T., Andreu-Sánchez S., Prins J.R., Kurilshikov A., Balder J.W. (2022). Age-dependent sex differences in cardiometabolic risk factors. Nat CardioVasc Res.

[40] Livingston G., Huntley J., Liu K.Y., Costafreda S.G., Selbæk G., Alladi S. (2024). Dementia prevention, intervention, and care: 2024 report of the Lancet standing Commission. Lancet.

[41] Akhavan Rezayat A., Dadgar Moghadam M., Ghasemi Nour M., Shirazinia M., Ghodsi H., Rouhbakhsh Zahmatkesh M.R. (2018). Association between smoking and non-alcoholic fatty liver disease: A systematic review and meta-analysis. Sage Open Med.

[42] Rosseel Y. (2012). lavaan: An R package for Structural Equation Modeling. J Stat Softw.

[43] SEM Measuring model fit (David A. Kenny). https://davidakenny.net/cm/fit.htm.

[44] Nakagawa S., Cuthill I.C. (2007). Effect size, confidence interval and statistical significance: A practical guide for biologists. Biol Rev Camb Philos Soc.

[45] Lampe L., Zhang R., Beyer F., Huhn S., Kharabian Masouleh S., Preusser S. (2019). Visceral obesity relates to deep white matter hyperintensities via inflammation. Ann Neurol.

[46] White W.B., Wakefield D.B., Moscufo N., Guttmann C.R.G., Kaplan R.F., Bohannon R.W. (2019). Effects of intensive versus standard ambulatory blood pressure control on cerebrovascular outcomes in older people (INFINITY). Circulation.

[47] Reitz C., Guzman V.A., Narkhede A., DeCarli C., Brickman A.M., Luchsinger J.A. (2017). Relation of dysglycemia to structural brain changes in a multiethnic elderly cohort. J Am Geriatr Soc.

[48] Tamura Y., Kimbara Y., Yamaoka T., Sato K., Tsuboi Y., Kodera R. (2017). White matter hyperintensity in elderly patients with diabetes mellitus is associated with cognitive impairment, functional disability, and a high glycoalbumin/glycohemoglobin ratio. Front Aging Neurosci, Internet.

[49] Walker K.A., Power M.C., Hoogeveen R.C., Folsom A.R., Ballantyne C.M., Knopman D.S. (2017). Midlife systemic inflammation, late-life white matter integrity, and cerebral small vessel disease: The Atherosclerosis Risk in Communities Study. Stroke.

[50] Dickie D.A., Ritchie S.J., Cox S.R., Sakka E., Royle N.A., Aribisala B.S. (2016). Vascular risk factors and progression of white matter hyperintensities in the Lothian Birth Cohort 1936. Neurobiol Aging.

[51] Schilling S., Tzourio C., Dufouil C., Zhu Y., Berr C., Alpérovitch A. (2014). Plasma lipids and cerebral small vessel disease. Neurology.

[52] Ciardullo S., Grassi G., Mancia G., Perseghin G. (2022). Nonalcoholic fatty liver disease and risk of incident hypertension: A systematic review and meta-analysis. Eur J Gastroenterol Hepatol.

[53] Ballestri S., Zona S., Targher G., Romagnoli D., Baldelli E., Nascimbeni F. (2016). Nonalcoholic fatty liver disease is associated with an almost 2-fold increased risk of incident type 2 diabetes and metabolic syndrome. Evidence from a systematic review and meta-analysis. J Gastroenterol Hepatol.

[54] Mantovani A., Petracca G., Beatrice G., Tilg H., Byrne C.D., Targher G. (2021). Non-alcoholic fatty liver disease and risk of incident diabetes mellitus: An updated meta-analysis of 501 022 adult individuals. Gut.

[55] Bril F., Sninsky J.J., Baca A.M., Superko H.R., Portillo Sanchez P., Biernacki D. (2016). Hepatic steatosis and insulin resistance, but not steatohepatitis, promote atherogenic dyslipidemia in NAFLD. J Clin Endocrinol Metab.

[56] Nam K.W., Kwon H.M., Jeong H.Y., Park J.H., Kwon H., Jeong S.M. (2019). Serum homocysteine level is related to cerebral small vessel disease in a healthy population. Neurology.

[57] Quek J., Chan K.E., Wong Z.Y., Tan C., Tan B., Lim W.H. (2023). Global prevalence of non-alcoholic fatty liver disease and non-alcoholic steatohepatitis in the overweight and obese population: A systematic review and meta-analysis. Lancet Gastroenterol Hepatol.

[58] Horn J.W., Feng T., Mørkedal B., Strand L.B., Horn J., Mukamal K., Janszky I. (2021). Obesity and risk for first ischemic stroke depends on metabolic syndrome: The HUNT study. Stroke.

[59] Sookoian S., Castaño G.O., Burgueño A.L., Rosselli M.S., Gianotti T.F., Mallardi P. (2010). Circulating levels and hepatic expression of molecular mediators of atherosclerosis in nonalcoholic fatty liver disease. Atherosclerosis.

[60] Simons N., Bijnen M., Wouters K.A.M., Rensen S.S., Beulens J.W.J., van Greevenbroek M.M.J. (2020). The endothelial function biomarker soluble E-selectin is associated with nonalcoholic fatty liver disease. Liver Int.

[61] Fricker Z.P., Pedley A., Massaro J.M., Vasan R.S., Hoffmann U., Benjamin E.J., Long M.T. (2019). Liver fat is associated with markers of inflammation and oxidative stress in analysis of data from the Framingham heart study. Clin Gastroenterol Hepatol.

[62] Zhao Y.C., Zhao G.J., Chen Z., She Z.G., Cai J., Li H. (2020). Nonalcoholic fatty liver disease: An Emerging Driver of Hypertension. Hypertension.

[63] Long M.T., Wang N., Larson M.G., Mitchell G.F., Palmisano J., Vasan R.S. (2015). Nonalcoholic fatty liver disease and vascular function: Cross-sectional analysis in the Framingham heart study. Arterioscler Thromb Vasc Biol.

[64] Lefebvre P., Staels B. (2021). Hepatic sexual dimorphism — Implications for non-alcoholic fatty liver disease. Nat Rev Endocrinol.

[65] Westerbacka J., Cornér A., Tiikkainen M., Tamminen M., Vehkavaara S., Häkkinen A.M. (2004). Women and men have similar amounts of liver and intra-abdominal fat, despite more subcutaneous fat in women: Implications for sex differences in markers of cardiovascular risk. Diabetologia.

[66] Subramaniapillai S., Suri S., Barth C., Maximov I.I., Voldsbekk I., van der Meer D. (2022). Sex- and age-specific associations between cardiometabolic risk and white matter brain age in the UK Biobank cohort. Hum Brain Mapp.

[67] Subramaniapillai S., Schindler L.S., Redmond P., Bastin M.E., Wardlaw J.M., Valdés Hernández M. (2024). Sex-dependent effects of cardiometabolic health and APOE4 on brain age: A Longitudinal Cohort Study. Neurology.

[68] Livingston G., Huntley J., Sommerlad A., Ames D., Ballard C., Banerjee S. (2020). Dementia prevention, intervention, and care: 2020 report of the Lancet Commission. Lancet.

[69] Gurholt T.P., Borda M.G., Parker N., Fominykh V., Kjelkenes R., Linge J. (2024). Linking sarcopenia, brain structure and cognitive performance: A large-scale UK Biobank study. Brain Commun.

[70] Sanyal A.J., Van Natta M.L., Clark J., Neuschwander-Tetri B.A., Diehl A., Dasarathy S. (2021). Prospective study of outcomes in adults with nonalcoholic fatty liver disease. N Engl J Med.

[71] Loomba R., Abdelmalek M.F., Armstrong M.J., Jara M., Kjær M.S., Krarup N. (2023). Semaglutide 2·4 mg once weekly in patients with non-alcoholic steatohepatitis-related cirrhosis: A randomised, placebo-controlled phase 2 trial. Lancet Gastroenterol Hepatol.

[72] Loomba R., Hartman M.L., Lawitz E.J., Vuppalanchi R., Boursier J., Bugianesi E. (2024). Tirzepatide for metabolic dysfunction–associated steatohepatitis with liver fibrosis. N Engl J Med.

[73] Cosentino F., Grant P.J., Aboyans V., Bailey C.J., Ceriello A., Delgado V. (2019). ESC Guidelines on diabetes, pre-diabetes, and cardiovascular diseases developed in collaboration with the EASD: The Task Force for diabetes, pre-diabetes, and cardiovascular diseases of the European Society of Cardiology (ESC) and the European Association for the Study of Diabetes (EASD). Eur Heart J.

[74] Mach F., Baigent C., Catapano A.L., Koskinas K.C., Casula M., Badimon L. (2019). ESC/EAS Guidelines for the management of dyslipidaemias: Lipid modification to reduce cardiovascular risk: The Task Force for the management of dyslipidaemias of the European Society of Cardiology (ESC) and European Atherosclerosis Society (EAS). Eur Heart J.

[75] Fry A., Littlejohns T.J., Sudlow C., Doherty N., Adamska L., Sprosen T. (2017). Comparison of sociodemographic and health-related characteristics of UK Biobank participants with those of the general population. Am J Epidemiol.

[76] Lyall D.M., Quinn T., Lyall L.M., Ward J., Anderson J.J., Smith D.J. (2022). Quantifying bias in psychological and physical health in the UK Biobank imaging sub-sample. Brain Commun.

