# Supplementary material for: Mediation Analyses Link Cardiometabolic Factors and Liver Fat With White Matter Hyperintensities and Cognitive Performance: A UK Biobank Study
Source: Biol Psychiatry Glob Open Sci. 2025 Mar 20;5(4):100488. doi: 10.1016/j.bpsgos.2025.100488 (PMC12052680; doi:10.1016/j.bpsgos.2025.100488)
Supplement: Figures S1–S11 and Tables S1–S31 [file mmc1.pdf]

SUPPLEMENTARY INFORMATION

Mediation analyses link cardiometabolic factors and liver fat with white matter hyperintensities and cognitive performance: A UK Biobank study

Askeland-Gjerde *et al.*

Supplementary tables 4
Table S1: Diagnoses of exclusion 4
Table S2: UK Biobank data fields........ 5
Table S3: Cardiometabolic medication 6
Table S4: Cardiometabolic outliers and missing data 8
Table S5: Cognitive test outliers and missing data 9
Table S6: Descriptive statistics for male and female participants........ 10
Table S7: Descriptive statistics for the total sample and the cognitive subsample........ 12
Table S8: Liver fat regression analyses........ 14

Table S9: Sex-interactions in liver fat regression analyses 15
Table S10: Liver fat regression analyses adjusted for cardiometabolic diagnoses 16
Table S11: Sex-interactions in liver fat regression analyses adjusted for cardiometabolic diagnoses 17
Table S12: White matter hyperintensities regression analyses 18
Table S13: Sex-interactions in white matter hyperintensities regression analyses 19
Table S14: White matter hyperintensities regression analyses adjusted for cardiometabolic diagnoses 20
Table S15: Sex-interaction in white matter hyperintensities regression analyses adjusted for cardiometabolic diagnoses 21
Table S16: General cognitive performance regression analyses 22
Table S17: Sex-interactions in general cognitive performance regression analyses 23
Table S18: General cognitive performance regression analyses adjusted for cardiometabolic diagnoses 24
Table S19: Sex-interactions in general cognitive performance regression analyses adjusted for cardiometabolic diagnoses 25
Table S20 General cognitive performance regression analyses without imputation 26
Table S21 Sex-interactions in general cognitive performance regression analyses without imputation 27
Table S22 Cognitive test regression analyses 28
Table S23 Sex-interactions in cognitive test regression analyses 35
Table S24: Mediation analyses on cardiometabolic factors, liver fat, and white matter hyperintensities 42
Table S25: Mediation analyses on cardiometabolic factors, liver fat, and white matter hyperintensities, adjusted for cardiometabolic diagnoses 43
Table S26: Sex-stratified mediation analyses on cardiometabolic factors, liver fat, and white matter hyperintensities 44
Table S27: Sex-stratified mediation analyses on cardiometabolic factors, liver fat, and white matter hyperintensities, adjusted for cardiometabolic diagnoses 45
Table S28: Mediation analyses on liver fat variables, white matter hyperintensities, and general cognitive performance 46
Table S29: Mediation analyses on liver fat variables, white matter hyperintensities, and cognitive tests 47
Table S30: Mediation analyses on liver fat variables, white matter hyperintensities, and general cognitive performance, without imputed data 49

Table S31: Mediation analyses on liver fat variables, white matter hyperintensities, and general cognitive performance, adjusted for cardiometabolic diagnoses 50

Supplementary figures 51

Figure S1: Flow chart of the study 52

Figure S2: Correlations of the cardiometabolic variables 53

Figure S3: Histograms of the cardiometabolic and imaging-related variables 54

Figure S4: Quantile-quantile plots of the cardiometabolic and imaging-related variables 55

Figure S5: Cardiometabolic principal component analysis 56

Figure S6: Cardiometabolic principal component analysis without imputed data 57

Figure S7: Correlations of the cognitive test variables 58

Figure S8: Histograms of the cognitive test variables 59

Figure S9: Quantile-quantile plots of the cognitive test variables 60

Figure S10: Cognitive principal component analysis 61

Figure S11: Cognitive principal component analysis without imputed data 62

Supplementary tables

Table S1: Diagnoses of exclusion

C22 Malignant neoplasm of liver and intrahepatic bile ducts	C23 Malignant neoplasm of gallbladder	C24 Malignant neoplasm of other and unspecified parts of biliary tract
C70 Malignant neoplasm of meninges	C71 Malignant neoplasm of brain	C72 Malignant neoplasm of spinal cord, cranial nerves and other parts of central nervous system
F00 Dementia in Alzheimer's disease	F01 Vascular dementia	F02 Dementia in other diseases classified elsewhere
F03 Unspecified dementia	F06.7 Mild cognitive disorder	G04 Encephalitis, myelitis and encephalomyelitis
G05 Encephalitis, myelitis and encephalomyelitis in diseases classified elsewhere	G06 Intracranial and intraspinal abscess and granuloma	G10 Huntington's disease
G11 Hereditary ataxia	G12 Spinal muscular atrophy and related syndromes	G13 Systemic atrophies primarily affecting central nervous system in diseases classified elsewhere
G14 Postpolio syndrome	G20 Parkinson's disease	G23 Other degenerative diseases of basal ganglia
G30 Alzheimer's disease	G31 Other degenerative diseases of nervous system, not elsewhere classified	G32 Other degenerative disorders of nervous system in diseases classified elsewhere
G35 Multiple sclerosis	G36 Other acute disseminated demyelination	G37 Other demyelinating diseases of central nervous system
I60 Subarachnoid haemorrhage	I61 Intracerebral haemorrhage	I62 Other nontraumatic intracranial haemorrhage
I63 Cerebral infarction	I64 Stroke, not specified as haemorrhage or infarction	I69 Sequelae of cerebrovascular disease
K70 Alcoholic liver disease	K71 Toxic liver disease	K72 Hepatic failure, not elsewhere classified
K73 Chronic hepatitis, not elsewhere classified	K74 Fibrosis and cirrhosis of liver	K75 Other inflammatory liver diseases
S06.2 Diffuse brain injury	S06.3 Focal brain injury	S06.4 Epidural haemorrhage
S06.5 Traumatic subdural haemorrhage	S06.6 Traumatic subarachnoid haemorrhage	S06.7 Intracranial injury with prolonged coma

Notes: ICD-10 diagnostic codes and names.

Table S2: UK Biobank data fields

41280 Date of first in-patient diagnosis - ICD10	41270 Diagnoses - ICD10	30870 Triglycerides
30780 LDL direct	30760 HDL cholesterol	30750 Glycated haemoglobin (HbA1c)
30710 C-reactive protein	30690 Cholesterol	26521 Volume of EstimatedTotalIntraCranial (whole brain)
25781 Total volume of white matter hyperintensities (from T1 and T2_FLAIR images)	24352 FR liver PDFF mean	23324 Number of symbol digit matches made correctly
22436 Liver PDFF (proton density fat fraction)	21004 Number of puzzles correct	21003 Age when attended assessment centre
21001 Body mass index (BMI)	21000 Ethnic background	20197 Number of word pairs correctly associated
20139 Number of letters correctly identified	20117 Alcohol drinker status	20116 Smoking status
20023 Mean time to correctly identify matches	20016 Fluid intelligence score	20003 Treatment/medication code
10722 Qualifications (pilot)	10137 Number of incorrect matches in round (pilot)	6373 Number of puzzles correctly solved
6350 Duration to complete alphanumeric path	6348 Duration to complete numeric path	6138 Qualifications
5364 Average weekly intake of other alcoholic drinks	4462 Average monthly intake of other alcoholic drinks	4451 Average monthly fortified wine intake
4440 Average monthly spirits intake	4429 Average monthly beer plus cider intake	4418 Average monthly champagne plus white wine intake
4407 Average monthly red wine intake	4282 Maximum digits remembered correctly	4080 Systolic blood pressure, automated reading
4079 Diastolic blood pressure, automated reading	1608 Average weekly fortified wine intake	1598 Average weekly spirits intake
1588 Average weekly beer plus cider intake	1578 Average weekly champagne plus white wine intake	1568 Average weekly red wine intake
1558 Alcohol intake frequency	399 Number of incorrect matches in round	94 Diastolic blood pressure, manual reading
93 Systolic blood pressure, manual reading	54 UK Biobank assessment centre	53 Date of attending assessment centre
48 Waist circumference	31 Sex	eid Participant identification

Notes: Field number and names of included UK Biobank data fields.

Table S3: Cardiometabolic medication

Antihypertensive drugs:					
hygroton k tablet combination pack	vasetic co-amilozide 5/50mg tablet	acezide 50mg tablets x56	capozide 50mg tablets x28	metoprolol tartrate+chlorthalidone 100mg/12.5mg tablet	tenoret 50 tablet
tenoretic tablet	sotalol hydrochloride+hydrochlorothiazide 80mg/12.5mg tablet	timolol maleate+co-amilozide 10mg/2.5mg/25mg tablet	beta-adalat capsule	tenif capsule	metoprolol tartrate+hydrochlorothiazide 100mg/12.5mg tablet
prestim tablet	propranolol hydrochloride+bendrofluazide 80mg/2.5mg capsule	acebutolol+hydrochlorothiazide 200mg/12.5mg tablet	atenolol+nifedipine 50mg/20mg m/r capsule	methyldopa+hydrochlorothiazide 250mg/15mg tablet	lisinopril
carace 2.5mg tablet	zestril 2.5mg tablet	quinapril	accuretic tablet	quinalapril+hydrochlorothiazide 10mg/12.5mg tablet	captopril
acepril 12.5mg tablet	capoten 12.5mg tablet	captopril+hydrochlorothiazide 25mg/12.5mg tablet	innovace 2.5mg tablet	innozide tablet	enalapril maleate+hydrochlorothiazide 20mg/12.5mg tablet
coversyl 2mg tablet	ramipril	staril 10mg tablet	cilazapril	trandolapril	gopten 500micrograms capsule
nifedipine	adalat 5mg capsule	coracten sr 10mg m/r capsule	tildiem 60mg m/r tablet	angiozem 60mg m/r tablet	adizem-60 m/r tablet
dilzem sr 60mg long acting m/r capsule	cardene 20mg capsule	isradipine	prescal 2.5mg tablet	istin 5mg tablet	lacidipine
motens 2mg tablet	zestoretic 10 tablet	carace 10 plus tablet	bisoprolol fumarate+hydrochlorothiazide 10mg/6.25mg tablet	lisinopril+hydrochlorothiazide 10mg/12.5mg tablet	hydroflumethiazide
methyclothiazide	polythiazide	diurexan 20mg tablet	aprinox 2.5mg tablet	chlorothiazide	hygroton 50mg tablet
cyclopenthiazide	hydrochlorothiazide	triamterene+benzthiazide 50mg/25mg capsule	dyazide tablet	dytide capsule	kalspare tablet
moduret 25 tablet	moduretic tablet	amiloride hcl+cyclopenthiazide 2.5mg/250micrograms tablet	amiloride hydrochloride+bumetanide 5mg/1mg tablet	atenolol	nimodipine
bisoprolol	amlodipine	diltiazem	nicardipine	metoprolol	oxprenolol
propranolol	adalate 10mg capsule	accupro 5mg tablet	capozide tablet	acezide tablet	verapamil
amiloride	enalapril	fosinopril	perindopril	antihypertensive	felodipine
carvedilol	slozem 120mg m/r capsule	beta-blocker	losartan	cozaar half strength 25mg tablet	angitil sr 90 m/r capsule
metazem 60mg m/r tablet	co-triamterzide	co-amilozide	co-flumactone	co-tenidone	adipine mr 10 m/r tablet
kentiazem 60mg m/r capsule	moexipril	perdix 7.5mg tablet	diltiazem hcl+hydrochlorothiazide 150mg/12.5mg m/r capsule	adizem-xl plus m/r capsule	amilamont 5mg/ml s/f oral solution
tensipine mr 10 m/r tablet	plendil 2.5mg m/r tablet	nisoldipine	syscor mr 10mg m/r tablet	valsartan	diovan 40mg capsule
atenolol+chlorthalidone	atenolol+bendrofluazide	atenolol+co-amilozide	natrilix sr 1.5mg m/r tablet	slofedipine 20mg m/r tablet	verapress mr 240 m/r tablet

losartan potassium+hydrochlorothiazide 50mg/12.5mg tablet	cozaar-comp 50mg/12.5mg tablet	hypapril 12.5mg tablet	viazem xl 120mg m/r capsule	irbesartan	aprovel 75mg tablet
lercanidipine	zanidip 10mg tablet	tarka 2mg/180mg m/r capsule	trandolapril+verapamil hydrochloride	candesartan cilexetil	amias 2mg tablet
nifedipress mr 10 m/r tablet	imidapril hydrochloride	tanatril 5mg tablet	nebivolol	felodipine+ramipril	triapin mite 2.5mg/2.5mg tablet
telmisartan	tensopril 12.5mg tablet	zemtard 120 xl m/r capsule	cardicor 1.25mg tablet	eprosartan	teveten 300mg tablet
micardis 20mg tablet	irbesartan+hydrochlorothiazide 150mg/12.5mg tablet	coaprovel 150mg/12.5mg tablet	zemret 180 xl m/r capsule	bi-carzem sr 60mg m/r capsule	cozaar 25mg tablet
horizem sr 90mg m/r capsule	perindopril+indapamide	coversyl plus 4mg/1.25mg tablet	atenolol+chlortalidone	telmisartan+hydrochlorothiazide 40mg/12.5mg tablet	micardisplus 40mg/12.5mg tablet
felotens xl 5mg m/r tablet	tritace 1.25mg tablet	felendil xl 5mg m/r tablet	vascalpha 5mg m/r tablet	olmesartan	olmetec 10mg tablet
bendroflumethiazide	bendroflumethiazide+potassium 2.5mg/7.7mmol m/r tablet	nadolol+bendroflumethiazide 40mg/5mg tablet	timolol maleate+bendroflumethiazide 10mg/2.5mg tablet	atenolol+bendroflumethiazide	cardioplen xl 5mg m/r tablet
lopace 2.5mg capsule	amlostin 5mg tablet	valsartan+hydrochlorothiazide 80mg/12.5mg tablet	co-diovan 80mg/12.5mg tablet	eplerenone	inspra 25mg tablet
**Lipid-lowering drugs:**					
bezafibrate	clofibrate	fenofibrate	simvastatin	lipostat 10mg tablet	ciprofibrate
lescol 20mg capsule	zocor 10mg tablet	fluvastatin	pravastatin	lipitor 10mg tablet	atorvastatin
bezafibrate product	rosuvastatin	crestor 10mg tablet	ezetimibe	ezetrol 10mg tablet	zocor heart-pro 10mg tablet
**Antidiabetic drugs:**					
acarbose	glucobay 50mg tablet	glipizide	glibenese 5mg tablet	minodiab 2.5mg tablet	tolbutamide
glucophage 500mg tablet	gliclazide	diamicron 80mg tablet	insulin product	metformin	glimepiride
amaryl 1mg tablet	glipizide product	repaglinide	pioglitazone	actos 15mg tablet	starlix 60mg tablet
nateglinide	rosiglitazone 1mg / metformin 500mg tablet				

Notes: Drugs considered as antihypertensive, lipid-lowering, and antidiabetic. Gathered from nurse-led interviews (UK Biobank data field 20003).

Table S4: Cardiometabolic outliers and missing data

Variables	Outliers	Missingness (%)
Body mass index	357	1.10
Waist circumference	144	0.44
Systolic blood pressure	191	0.59
Diastolic blood pressure	138	0.43
Pulse pressure	293	0.90
C-reactive protein	542	1.67
Glycated hemoglobin	473	1.46
High-density lipoprotein cholesterol	238	0.73
Low-density lipoprotein cholesterol	129	0.40
Total cholesterol	144	0.44
Triglycerides	266	0.82

Notes: Outliers were defined as values deviating more than 3 standard deviations from the mean.

Table S5: Cognitive test outliers and missing data

Variables	Outliers	Missingness (%)
Numeric memory	107	1.25
Fluid intelligence	51	0.73
Trail making test B	202	15.29
Matrix test	30	12.48
Symbol digit substitution	130	12.80
Tower rearranging	33	13.21
Paired associate learning	0	0.00
Pairs matching	17	0.05

Notes: Outliers were defined as values deviating more than 3 standard deviations from the mean.

Table S6: Descriptive statistics for male and female participants

Variables	Males (n=15,398)	Females (n=17,063)	Test statistic	P-value
**Demographic variables:**				
**Age baseline**	55.55±7.60	54.23±7.38	15.84	$2.60e^{-56}$
**Age imaging**	64.89±7.78	63.59±7.57	15.14	$1.35e^{-51}$
**Follow-up time**	9.33±1.99	9.36±1.97	-1.08	$2.79e^{-01}$
**Higher education**	7,474 (48.54%)	7,606 (44.58%)	50.94	$9.53e^{-13}$
**Intermediate education**	4,479 (29.09%)	6,152 (36.05%)	178.03	$1.30e^{-40}$
**Asian**	240 (1.56%)	182 (1.07%)	14.89	$1.14e^{-04}$
**Black**	77 (0.50%)	105 (0.62%)	1.73	$1.89e^{-01}$
**Mixed**	46 (0.30%)	106 (0.62%)	17.38	$3.07e^{-05}$
**Other ethnicity**	68 (0.44%)	97 (0.57%)	2.33	$1.27e^{-01}$
**White**	14,914 (96.86%)	16,536 (96.91%)	0.06	$8.02e^{-01}$
**Cheadle**	8,796 (57.12%)	9,339 (54.73%)	18.68	$1.54e^{-05}$
**Newcastle**	4,065 (26.40%)	4,828 (28.30%)	14.53	$1.38e^{-04}$
**Reading**	2,513 (16.32%)	2,872 (16.83%)	1.49	$2.22e^{-01}$
**Bristol**	24 (0.16%)	24 (0.14%)	0.04	$8.33e^{-01}$
**Behavioral risk factors:**				
**Current smoker**	587 (3.81%)	471 (2.76%)	28.07	$1.17e^{-07}$
**Former smoker**	5,679 (36.88%)	5,279 (30.94%)	127.58	$1.39e^{-29}$
**Alcohol consumption at baseline**	169.23±155.86	79.88±85.42	63.10	$0.00e^{+00}$
**Alcohol consumption at imaging**	145.17±143.42	69.49±79.17	57.99	$0.00e^{+00}$
**Cardiometabolic variables:**				
**Body mass index**	27.08±3.64	26.03±4.52	23.05	$1.32e^{-116}$
**Waist circumference**	94.53±9.98	81.68±11.13	109.63	$0.00e^{+00}$
**Systolic blood pressure**	138.81±16.40	131.29±17.90	39.50	$0.00e^{+00}$
**Diastolic blood pressure**	83.61±9.63	79.45±9.69	38.69	$3.55e^{-305}$
**Pulse pressure**	55.20±11.52	51.83±12.72	25.02	$7.29e^{-137}$
**C-reactive protein**	1.94±3.34	2.14±3.76	-5.01	$5.50e^{-07}$
**Glycated hemoglobin**	35.20±5.50	34.71±4.56	8.67	$4.37e^{-18}$
**High-density lipoprotein cholesterol**	1.31±0.30	1.64±0.37	-88.93	$0.00e^{+00}$
**Low-density lipoprotein cholesterol**	3.57±0.82	3.59±0.82	-2.01	$4.47e^{-02}$
**Total cholesterol**	5.60±1.07	5.85±1.07	-20.89	$2.82e^{-96}$
**Triglycerides**	1.88±1.05	1.41±0.76	46.53	$0.00e^{+00}$
**Cardiometabolic diagnoses:**				
**Hypertension**	8,366 (54.33%)	6,259 (36.68%)	1,017.83	$2.39e^{-223}$
**Diabetes**	712 (4.62%)	393 (2.30%)	131.87	$1.60e^{-30}$

Variables	Males (n=15,398)	Females (n=17,063)	Test statistic	P-value
**Dyslipidemia**	9,524 (61.85%)	7,993 (46.84%)	733.25	$1.76e^{-161}$
**Liver fat and brain variables:**				
**Liver fat**	4.70±4.18	3.87±3.99	18.24	$6.49e^{-74}$
**Steatosis**	4,385 (28.48%)	3,216 (18.85%)	418.02	$6.58e^{-93}$
**Nonalcoholic fatty liver disease**	2,606 (16.92%)	2,410 (14.12%)	48.36	$3.54e^{-12}$
**Metabolic dysfunction-associated fatty liver disease**	4,127 (26.80%)	2,907 (17.04%)	454.16	$8.98e^{-101}$
**Metabolic dysfunction-associated steatotic liver disease**	2,547 (16.54%)	2,336 (13.69%)	51.24	$8.16e^{-13}$
**White matter hyperintensities**	5,558.24±7,088.67	4,602.31±6,192.23	12.88	$7.56e^{-38}$

Notes: Means and standard deviations, and numbers and proportions are displayed for continuous and categorical measures, respectively. Test statistics were derived from Welch two sample tests and Pearson's chi-squared test with Yates' continuity correction for continuous and categorical measures, respectively.

Table S7: Descriptive statistics for the total sample and the cognitive subsample

Variables	Sample (n=32,461)	Subsample (n=23,354)	Test statistic	P-value
**Demographic variables:**				
**Male**	15,398 (47.44%)	11,079 (47.44%)	0.00	$9.99e^{-01}$
**Female**	17,063 (52.56%)	12,275 (52.56%)	0.00	$1.00e^{+00}$
**Age baseline**	54.86±7.51	54.48±7.48	5.90	$3.73e^{-09}$
**Age imaging**	64.21±7.70	64.58±7.61	-5.74	$9.33e^{-09}$
**Follow-up time**	9.35±1.98	10.11±1.47	-52.00	$0.00e^{+00}$
**Higher education**	15,080 (46.46%)	11,251 (48.18%)	16.06	$6.14e^{-05}$
**Intermediate education**	10,631 (32.75%)	7,509 (32.15%)	2.18	$1.40e^{-01}$
**Asian**	422 (1.30%)	269 (1.15%)	2.32	$1.28e^{-01}$
**Black**	182 (0.56%)	129 (0.55%)	0.01	$9.42e^{-01}$
**Mixed**	152 (0.47%)	109 (0.47%)	0.00	$1.00e^{+00}$
**Other ethnicity**	165 (0.51%)	118 (0.51%)	0.00	$1.00e^{+00}$
**White**	31,450 (96.89%)	22,670 (97.07%)	1.53	$2.16e^{-01}$
**Cheadle**	18,135 (55.87%)	10,004 (42.84%)	922.04	$1.58e^{-202}$
**Newcastle**	8,893 (27.40%)	8,242 (35.29%)	397.63	$1.81e^{-88}$
**Reading**	5,385 (16.59%)	5,064 (21.68%)	231.34	$3.05e^{-52}$
**Bristol**	48 (0.15%)	44 (0.19%)	1.12	$2.90e^{-01}$
**Behavioral risk factors:**				
**Current smoker**	1,058 (3.26%)	703 (3.01%)	2.68	$1.02e^{-01}$
**Former smoker**	10,958 (33.76%)	7,791 (33.36%)	0.94	$3.32e^{-01}$
**Alcohol consumption at baseline**	122.26±131.71	121.93±129.28	0.30	$7.68e^{-01}$
**Alcohol consumption at imaging**	105.39±120.33	104.62±118.68	0.74	$4.57e^{-01}$
**Cardiometabolic variables:**				
**Body mass index**	26.53±4.16	26.46±4.13	1.99	$4.65e^{-02}$
**Waist circumference**	87.77±12.39	87.59±12.31	1.72	$8.57e^{-02}$
**Systolic blood pressure**	134.85±17.61	134.47±17.49	2.57	$1.03e^{-02}$
**Diastolic blood pressure**	81.42±9.88	81.37±9.90	0.57	$5.68e^{-01}$
**Pulse pressure**	53.43±12.28	53.09±12.11	3.23	$1.23e^{-03}$
**C-reactive protein**	2.04±3.57	1.99±3.49	1.72	$8.58e^{-02}$
**Glycated hemoglobin**	34.94±5.03	34.87±5.02	1.78	$7.57e^{-02}$
**High-density lipoprotein cholesterol**	1.48±0.38	1.48±0.38	-1.08	$2.80e^{-01}$
**Low-density lipoprotein cholesterol**	3.58±0.82	3.58±0.82	0.65	$5.13e^{-01}$
**Total cholesterol**	5.73±1.07	5.72±1.07	0.52	$6.02e^{-01}$
**Triglycerides**	1.63±0.94	1.62±0.93	1.68	$9.39e^{-02}$
**Cardiometabolic diagnoses:**				

Variables	Sample (n=32,461)	Subsample (n=23,354)	Test statistic	P-value
**Hypertension**	14,625 (45.05%)	10,320 (44.19%)	4.07	$4.36e^{-02}$
**Diabetes**	1,105 (3.40%)	758 (3.25%)	1.01	$3.15e^{-01}$
**Dyslipidemia**	17,517 (53.96%)	12,390 (53.05%)	4.49	$3.41e^{-02}$
**Liver fat and brain variables:**				
**Liver fat**	4.26±4.10	4.25±3.97	0.40	$6.86e^{-01}$
**Steatosis**	7,601 (23.42%)	5,404 (23.14%)	0.56	$4.52e^{-01}$
**Nonalcoholic fatty liver disease**	5,016 (15.45%)	3,558 (15.24%)	0.48	$4.90e^{-01}$
**Metabolic dysfunction-associated fatty liver disease**	7,034 (21.67%)	4,954 (21.21%)	1.65	$1.99e^{-01}$
**Metabolic dysfunction-associated steatotic liver disease**	4,883 (15.04%)	3,451 (14.78%)	0.73	$3.91e^{-01}$
**White matter hyperintensities**	5,055.76±6,649.63	5,278.77±6,814.39	-3.85	$1.17e^{-04}$

Notes: Means and standard deviations, and numbers and proportions are displayed for continuous and categorical measures, respectively. Test statistics were derived from Welch two sample tests and Pearson's chi-squared test with Yates' continuity correction for continuous and categorical measures, respectively.

Table S8: Liver fat regression analyses

	β	SE	T	P	R	LCI	UCI
**Cardiometabolic variables:**							
**Body mass index**	0.221	0.003	78.405	$0.00e^{+00}$	0.399	0.390	0.408
**Waist circumference**	0.262	0.003	80.153	$0.00e^{+00}$	0.407	0.397	0.416
**Systolic blood pressure**	0.076	0.003	23.316	$2.95e^{-119}$	0.128	0.118	0.139
**Diastolic blood pressure**	0.103	0.003	33.558	$9.77e^{-243}$	0.183	0.173	0.194
**Pulse pressure**	0.018	0.003	5.521	$3.39e^{-08}$	0.031	0.020	0.042
**C-reactive protein**	0.239	0.005	47.759	$0.00e^{+00}$	0.256	0.246	0.266
**Glycated hemoglobin**	0.085	0.003	27.554	$3.22e^{-165}$	0.151	0.141	0.162
**High-density lipoprotein cholesterol**	-0.169	0.003	-50.485	$0.00e^{+00}$	-0.270	-0.280	-0.260
**Low-density lipoprotein cholesterol**	0.045	0.003	14.675	$1.34e^{-48}$	0.081	0.070	0.092
**Total cholesterol**	0.022	0.003	6.905	$5.11e^{-12}$	0.038	0.027	0.049
**Triglycerides**	0.605	0.010	61.503	$0.00e^{+00}$	0.323	0.313	0.333
**Cardiometabolic principal components:**							
**Principal component 1**	0.136	0.002	70.454	$0.00e^{+00}$	0.364	0.355	0.374
**Principal component 2**	0.053	0.002	23.219	$2.72e^{-118}$	0.128	0.117	0.139
**Principal component 3**	-0.102	0.003	-40.812	$0.00e^{+00}$	-0.221	-0.231	-0.211
**Cardiometabolic principal components without imputed data:**							
**Principal component 1**	0.130	0.002	64.421	$0.00e^{+00}$	0.337	0.327	0.346
**Principal component 2**	-0.049	0.002	-20.577	$1.93e^{-93}$	-0.113	-0.124	-0.103
**Principal component 3**	-0.102	0.003	-39.689	$0.00e^{+00}$	-0.215	-0.226	-0.205

Notes: Results derived from multiple linear regression analyses adjusted for age, age^2, sex, interactions between age and sex, interactions between age^2 and sex, site, smoking status, and alcohol consumption.

Abbreviations: β, Standardized beta; SE, Standard error; T, T-statistic; P, P-value; R, Partial correlation coefficient; LCI, Lower confidence interval; UCI, Upper confidence interval

Table S9: Sex-interactions in liver fat regression analyses

	β	SE	T	P	R	LCI	UCI
**Cardiometabolic variables:**							
**Body mass index**	0.028	0.006	4.734	$2.21e^{-06}$	0.026	0.015	0.037
**Waist circumference**	-0.011	0.007	-1.693	$9.05e^{-02}$	-0.009	-0.020	0.001
**Systolic blood pressure**	-0.020	0.006	-3.091	$2.00e^{-03}$	-0.017	-0.028	-0.006
**Diastolic blood pressure**	-0.025	0.006	-4.125	$3.72e^{-05}$	-0.023	-0.034	-0.012
**Pulse pressure**	-0.017	0.007	-2.573	$1.01e^{-02}$	-0.014	-0.025	-0.003
**C-reactive protein**	-0.087	0.010	-8.613	$7.41e^{-18}$	-0.048	-0.059	-0.037
**Glycated hemoglobin**	-0.021	0.006	-3.398	$6.79e^{-04}$	-0.019	-0.030	-0.008
**High-density lipoprotein cholesterol**	-0.014	0.007	-2.026	$4.27e^{-02}$	-0.011	-0.022	-0.000
**Low-density lipoprotein cholesterol**	-0.034	0.006	-5.439	$5.40e^{-08}$	-0.030	-0.041	-0.019
**Total cholesterol**	-0.011	0.006	-1.799	$7.21e^{-02}$	-0.010	-0.021	0.001
**Triglycerides**	-0.252	0.020	-12.706	$6.73e^{-37}$	-0.070	-0.081	-0.060
**Cardiometabolic principal components:**							
**Principal component 1**	-0.007	0.004	-1.789	$7.37e^{-02}$	-0.010	-0.021	0.001
**Principal component 2**	-0.013	0.005	-2.924	$3.46e^{-03}$	-0.016	-0.027	-0.005
**Principal component 3**	0.011	0.005	2.178	$2.94e^{-02}$	0.012	0.001	0.023
**Cardiometabolic principal components without imputed data:**							
**Principal component 1**	-0.005	0.004	-1.203	$2.29e^{-01}$	-0.007	-0.018	0.004
**Principal component 2**	0.009	0.005	1.938	$5.26e^{-02}$	0.011	-0.000	0.022
**Principal component 3**	0.006	0.005	1.114	$2.65e^{-01}$	0.006	-0.005	0.017

Notes: Results derived from multiple linear regression analyses adjusted for age, age^2, sex, interactions between age and sex, interactions between age^2 and sex, site, smoking status, and alcohol consumption.

Abbreviations: β, Standardized beta; SE, Standard error; T, T-statistic; P, P-value; R, Partial correlation coefficient; LCI, Lower confidence interval; UCI, Upper confidence interval

Table S10: Liver fat regression analyses adjusted for cardiometabolic diagnoses

	β	SE	T	P	R	LCI	UCI
**Cardiometabolic variables:**							
**Body mass index**	0.212	0.003	73.258	$0.00e^{+00}$	0.377	0.367	0.386
**Waist circumference**	0.251	0.003	75.099	$0.00e^{+00}$	0.385	0.376	0.394
**Systolic blood pressure**	0.066	0.003	20.301	$4.62e^{-91}$	0.112	0.101	0.123
**Diastolic blood pressure**	0.096	0.003	31.195	$1.60e^{-210}$	0.171	0.160	0.181
**Pulse pressure**	0.010	0.003	3.092	$1.99e^{-03}$	0.017	0.006	0.028
**C-reactive protein**	0.227	0.005	45.699	$0.00e^{+00}$	0.246	0.236	0.256
**Glycated hemoglobin**	0.063	0.003	17.950	$1.06e^{-71}$	0.099	0.088	0.110
**High-density lipoprotein cholesterol**	-0.158	0.003	-47.163	$0.00e^{+00}$	-0.253	-0.263	-0.243
**Low-density lipoprotein cholesterol**	0.070	0.003	22.028	$9.36e^{-107}$	0.121	0.111	0.132
**Total cholesterol**	0.045	0.003	14.049	$1.06e^{-44}$	0.078	0.067	0.089
**Triglycerides**	0.579	0.010	59.074	$0.00e^{+00}$	0.312	0.302	0.321
**Cardiometabolic principal components:**							
**Principal component 1**	0.131	0.002	66.815	$0.00e^{+00}$	0.348	0.338	0.357
**Principal component 2**	0.039	0.002	16.026	$1.39e^{-57}$	0.089	0.078	0.099
**Principal component 3**	-0.105	0.002	-42.521	$0.00e^{+00}$	-0.230	-0.240	-0.219
**Cardiometabolic principal components without imputed data:**							
**Principal component 1**	0.125	0.002	61.514	$0.00e^{+00}$	0.323	0.313	0.333
**Principal component 2**	-0.037	0.003	-14.642	$2.21e^{-48}$	-0.081	-0.092	-0.070
**Principal component 3**	-0.105	0.003	-41.105	$0.00e^{+00}$	-0.222	-0.233	-0.212

Notes: Results derived from multiple linear regression analyses adjusted for age, age^2, sex, interactions between age and sex, interactions between age^2 and sex, site, smoking status, alcohol consumption, hypertension, diabetes, and dyslipidemia.

Abbreviations: β, Standardized beta; SE, Standard error; T, T-statistic; P, P-value; R, Partial correlation coefficient; LCI, Lower confidence interval; UCI, Upper confidence interval

Table S11: Sex-interactions in liver fat regression analyses adjusted for cardiometabolic diagnoses

	β	SE	T	P	R	LCI	UCI
**Cardiometabolic variables:**							
**Body mass index**	0.021	0.006	3.640	$2.73e^{-04}$	0.020	0.009	0.031
**Waist circumference**	-0.016	0.007	-2.412	$1.59e^{-02}$	-0.013	-0.024	-0.003
**Systolic blood pressure**	-0.020	0.006	-3.119	$1.82e^{-03}$	-0.017	-0.028	-0.006
**Diastolic blood pressure**	-0.024	0.006	-3.874	$1.07e^{-04}$	-0.022	-0.032	-0.011
**Pulse pressure**	-0.018	0.007	-2.697	$7.01e^{-03}$	-0.015	-0.026	-0.004
**C-reactive protein**	-0.083	0.010	-8.355	$6.83e^{-17}$	-0.046	-0.057	-0.035
**Glycated hemoglobin**	-0.026	0.006	-4.169	$3.06e^{-05}$	-0.023	-0.034	-0.012
**High-density lipoprotein cholesterol**	-0.008	0.007	-1.192	$2.33e^{-01}$	-0.007	-0.017	0.004
**Low-density lipoprotein cholesterol**	-0.013	0.006	-2.077	$3.78e^{-02}$	-0.012	-0.022	-0.001
**Total cholesterol**	0.007	0.006	1.109	$2.67e^{-01}$	0.006	-0.005	0.017
**Triglycerides**	-0.239	0.020	-12.173	$5.13e^{-34}$	-0.067	-0.078	-0.057
**Cardiometabolic principal components:**							
**Principal component 1**	-0.008	0.004	-2.013	$4.41e^{-02}$	-0.011	-0.022	-0.000
**Principal component 2**	-0.022	0.005	-4.770	$1.85e^{-06}$	-0.026	-0.037	-0.016
**Principal component 3**	0.007	0.005	1.408	$1.59e^{-01}$	0.008	-0.003	0.019
**Cardiometabolic principal components without imputed data:**							
**Principal component 1**	-0.005	0.004	-1.309	$1.91e^{-01}$	-0.007	-0.018	0.004
**Principal component 2**	0.017	0.005	3.546	$3.92e^{-04}$	0.020	0.009	0.031
**Principal component 3**	0.003	0.005	0.544	$5.87e^{-01}$	0.003	-0.008	0.014

Notes: Results derived from multiple linear regression analyses adjusted for age, age^2, sex, interactions between age and sex, interactions between age^2 and sex, site, smoking status, alcohol consumption, hypertension, diabetes, and dyslipidemia.

Abbreviations: β, Standardized beta; SE, Standard error; T, T-statistic; P, P-value; R, Partial correlation coefficient; LCI, Lower confidence interval; UCI, Upper confidence interval

Table S12: White matter hyperintensities regression analyses

	β	SE	T	P	R	LCI	UCI
**Cardiometabolic variables:**							
**Body mass index**	0.100	0.005	21.242	$1.88e^{-99}$	0.117	0.106	0.128
**Waist circumference**	0.114	0.005	20.839	$8.07e^{-96}$	0.115	0.104	0.126
**Systolic blood pressure**	0.108	0.005	21.585	$1.31e^{-102}$	0.119	0.108	0.130
**Diastolic blood pressure**	0.119	0.005	24.890	$1.79e^{-135}$	0.137	0.126	0.148
**Pulse pressure**	0.052	0.005	10.175	$2.79e^{-24}$	0.056	0.046	0.067
**C-reactive protein**	0.082	0.008	10.306	$7.25e^{-25}$	0.057	0.046	0.068
**Glycated hemoglobin**	0.052	0.005	10.923	$1.00e^{-27}$	0.061	0.050	0.071
**High-density lipoprotein cholesterol**	-0.051	0.005	-9.458	$3.34e^{-21}$	-0.052	-0.063	-0.042
**Low-density lipoprotein cholesterol**	0.002	0.005	0.400	$6.89e^{-01}$	0.002	-0.009	0.013
**Total cholesterol**	-0.004	0.005	-0.897	$3.70e^{-01}$	-0.005	-0.016	0.006
**Triglycerides**	0.147	0.016	9.175	$4.78e^{-20}$	0.051	0.040	0.062
**Cardiometabolic principal components:**							
**Principal component 1**	0.085	0.003	27.007	$6.95e^{-159}$	0.148	0.138	0.159
**Principal component 2**	0.026	0.004	7.207	$5.85e^{-13}$	0.040	0.029	0.051
**Principal component 3**	0.009	0.004	2.322	$2.02e^{-02}$	0.013	0.002	0.024
**Cardiometabolic principal components without imputed data:**							
**Principal component 1**	0.082	0.003	24.457	$8.10e^{-131}$	0.135	0.124	0.145
**Principal component 2**	-0.027	0.004	-7.185	$6.90e^{-13}$	-0.040	-0.051	-0.029
**Principal component 3**	0.008	0.004	2.047	$4.07e^{-02}$	0.011	0.000	0.022
**Liver fat variables:**							
**Liver fat**	0.164	0.009	19.246	$4.34e^{-82}$	0.106	0.095	0.117
**Nonalcoholic fatty liver disease**	0.161	0.013	12.129	$8.78e^{-34}$	0.067	0.056	0.078
**Metabolic dysfunction-associated fatty liver disease**	0.180	0.011	15.720	$1.77e^{-55}$	0.087	0.076	0.098
**Metabolic dysfunction-associated steatotic liver disease**	0.165	0.013	12.306	$1.00e^{-34}$	0.068	0.057	0.079

Notes: Results derived from multiple linear regression analyses adjusted for age, age^2, sex, interactions between age and sex, interactions between age^2 and sex, site, smoking status, alcohol consumption, and intracranial volume.

Abbreviations: β, Standardized beta; SE, Standard error; T, T-statistic; P, P-value; R, Partial correlation coefficient; LCI, Lower confidence interval; UCI, Upper confidence interval

Table S13: Sex-interactions in white matter hyperintensities regression analyses

	β	SE	T	P	R	LCI	UCI
**Cardiometabolic variables:**							
**Body mass index**	0.080	0.010	8.203	$2.43e^{-16}$	0.045	0.035	0.056
**Waist circumference**	0.071	0.011	6.422	$1.36e^{-10}$	0.036	0.025	0.046
**Systolic blood pressure**	-0.017	0.010	-1.733	$8.31e^{-02}$	-0.010	-0.020	0.001
**Diastolic blood pressure**	-0.020	0.010	-2.097	$3.60e^{-02}$	-0.012	-0.023	-0.001
**Pulse pressure**	-0.019	0.010	-1.870	$6.15e^{-02}$	-0.010	-0.021	0.000
**C-reactive protein**	0.025	0.016	1.546	$1.22e^{-01}$	0.009	-0.002	0.019
**Glycated hemoglobin**	0.001	0.010	0.150	$8.81e^{-01}$	0.001	-0.010	0.012
**High-density lipoprotein cholesterol**	-0.037	0.011	-3.441	$5.81e^{-04}$	-0.019	-0.030	-0.008
**Low-density lipoprotein cholesterol**	-0.028	0.010	-2.880	$3.97e^{-03}$	-0.016	-0.027	-0.005
**Total cholesterol**	-0.025	0.010	-2.557	$1.06e^{-02}$	-0.014	-0.025	-0.003
**Triglycerides**	-0.036	0.032	-1.109	$2.67e^{-01}$	-0.006	-0.017	0.005
**Cardiometabolic principal components:**							
**Principal component 1**	0.018	0.006	2.850	$4.37e^{-03}$	0.016	0.005	0.027
**Principal component 2**	0.029	0.007	4.128	$3.67e^{-05}$	0.023	0.012	0.034
**Principal component 3**	-0.029	0.008	-3.627	$2.87e^{-04}$	-0.020	-0.031	-0.009
**Cardiometabolic principal components without imputed data:**							
**Principal component 1**	0.015	0.007	2.195	$2.82e^{-02}$	0.012	0.001	0.023
**Principal component 2**	-0.033	0.008	-4.348	$1.38e^{-05}$	-0.024	-0.035	-0.013
**Principal component 3**	-0.036	0.008	-4.350	$1.37e^{-05}$	-0.024	-0.035	-0.013
**Liver fat variables:**							
**Liver fat**	0.038	0.017	2.255	$2.41e^{-02}$	0.013	0.002	0.023
**Nonalcoholic fatty liver disease**	0.039	0.026	1.521	$1.28e^{-01}$	0.008	-0.002	0.019
**Metabolic dysfunction-associated fatty liver disease**	0.037	0.023	1.610	$1.07e^{-01}$	0.009	-0.002	0.020
**Metabolic dysfunction-associated steatotic liver disease**	0.044	0.026	1.699	$8.93e^{-02}$	0.009	-0.001	0.020

Notes: Results derived from multiple linear regression analyses adjusted for age, age^2, sex, interactions between age and sex, interactions between age^2 and sex, site, smoking status, alcohol consumption, and intracranial volume.

Abbreviations: β, Standardized beta; SE, Standard error; T, T-statistic; P, P-value; R, Partial correlation coefficient; LCI, Lower confidence interval; UCI, Upper confidence interval

Table S14: White matter hyperintensities regression analyses adjusted for cardiometabolic diagnoses

	β	SE	T	P	R	LCI	UCI
**Cardiometabolic variables:**							
**Body mass index**	0.080	0.005	16.711	$1.99e^{-62}$	0.092	0.082	0.103
**Waist circumference**	0.092	0.006	16.369	$5.55e^{-60}$	0.091	0.080	0.101
**Systolic blood pressure**	0.094	0.005	18.773	$3.28e^{-78}$	0.104	0.093	0.114
**Diastolic blood pressure**	0.108	0.005	22.469	$5.84e^{-111}$	0.124	0.113	0.134
**Pulse pressure**	0.041	0.005	8.082	$6.58e^{-16}$	0.045	0.034	0.056
**C-reactive protein**	0.067	0.008	8.452	$2.97e^{-17}$	0.047	0.036	0.058
**Glycated hemoglobin**	0.029	0.005	5.350	$8.86e^{-08}$	0.030	0.019	0.041
**High-density lipoprotein cholesterol**	-0.034	0.005	-6.388	$1.70e^{-10}$	-0.035	-0.046	-0.025
**Low-density lipoprotein cholesterol**	0.027	0.005	5.483	$4.21e^{-08}$	0.030	0.020	0.041
**Total cholesterol**	0.022	0.005	4.354	$1.34e^{-05}$	0.024	0.013	0.035
**Triglycerides**	0.111	0.016	6.976	$3.09e^{-12}$	0.039	0.028	0.050
**Cardiometabolic principal components:**							
**Principal component 1**	0.075	0.003	23.536	$1.83e^{-121}$	0.130	0.119	0.140
**Principal component 2**	0.003	0.004	0.851	$3.95e^{-01}$	0.005	-0.006	0.016
**Principal component 3**	0.005	0.004	1.150	$2.50e^{-01}$	0.006	-0.004	0.017
**Cardiometabolic principal components without imputed data:**							
**Principal component 1**	0.073	0.003	21.629	$5.82e^{-103}$	0.119	0.108	0.130
**Principal component 2**	-0.008	0.004	-1.996	$4.60e^{-02}$	-0.011	-0.022	-0.000
**Principal component 3**	0.004	0.004	0.965	$3.35e^{-01}$	0.005	-0.006	0.016
**Liver fat variables:**							
**Liver fat**	0.138	0.009	16.158	$1.68e^{-58}$	0.089	0.079	0.100
**Nonalcoholic fatty liver disease**	0.126	0.013	9.512	$1.99e^{-21}$	0.053	0.042	0.064
**Metabolic dysfunction-associated fatty liver disease**	0.143	0.012	12.389	$3.61e^{-35}$	0.069	0.058	0.079
**Metabolic dysfunction-associated steatotic liver disease**	0.129	0.013	9.590	$9.39e^{-22}$	0.053	0.042	0.064

Notes: Results derived from multiple linear regression analyses adjusted for age, age^2, sex, interactions between age and sex, interactions between age^2 and sex, site, smoking status, alcohol consumption, intracranial volume, hypertension, diabetes, and dyslipidemia.

Abbreviations: β, Standardized beta; SE, Standard error; T, T-statistic; P, P-value; R, Partial correlation coefficient; LCI, Lower confidence interval; UCI, Upper confidence interval

Table S15: Sex-interaction in white matter hyperintensities regression analyses adjusted for cardiometabolic diagnoses

	β	SE	T	P	R	LCI	UCI
**Cardiometabolic variables:**							
**Body mass index**	0.069	0.010	7.084	1.43e^{-12}	0.039	0.028	0.050
**Waist circumference**	0.062	0.011	5.649	1.63e^{-08}	0.031	0.020	0.042
**Systolic blood pressure**	-0.016	0.010	-1.610	1.07e^{-01}	-0.009	-0.020	0.002
**Diastolic blood pressure**	-0.018	0.009	-1.876	6.07e^{-02}	-0.010	-0.021	0.000
**Pulse pressure**	-0.018	0.010	-1.752	7.99e^{-02}	-0.010	-0.021	0.001
**C-reactive protein**	0.030	0.016	1.917	5.53e^{-02}	0.011	-0.000	0.022
**Glycated hemoglobin**	-0.003	0.010	-0.293	7.70e^{-01}	-0.002	-0.013	0.009
**High-density lipoprotein cholesterol**	-0.029	0.011	-2.648	8.09e^{-03}	-0.015	-0.026	-0.004
**Low-density lipoprotein cholesterol**	-0.004	0.010	-0.387	6.99e^{-01}	-0.002	-0.013	0.009
**Total cholesterol**	-0.003	0.010	-0.304	7.61e^{-01}	-0.002	-0.013	0.009
**Triglycerides**	-0.014	0.032	-0.447	6.55e^{-01}	-0.002	-0.013	0.008
**Cardiometabolic principal components:**							
**Principal component 1**	0.019	0.006	2.906	3.66e^{-03}	0.016	0.005	0.027
**Principal component 2**	0.015	0.007	2.170	3.00e^{-02}	0.012	0.001	0.023
**Principal component 3**	-0.034	0.008	-4.274	1.92e^{-05}	-0.024	-0.035	-0.013
**Cardiometabolic principal components without imputed data:**							
**Principal component 1**	0.015	0.007	2.283	2.24e^{-02}	0.013	0.002	0.024
**Principal component 2**	-0.020	0.008	-2.608	9.12e^{-03}	-0.014	-0.025	-0.004
**Principal component 3**	-0.040	0.008	-4.804	1.56e^{-06}	-0.027	-0.038	-0.016
**Liver fat variables:**							
**Liver fat**	0.036	0.017	2.161	3.07e^{-02}	0.012	0.001	0.023
**Nonalcoholic fatty liver disease**	0.043	0.026	1.672	9.45e^{-02}	0.009	-0.002	0.020
**Metabolic dysfunction-associated fatty liver disease**	0.038	0.023	1.687	9.17e^{-02}	0.009	-0.002	0.020
**Metabolic dysfunction-associated steatotic liver disease**	0.049	0.026	1.869	6.17e^{-02}	0.010	-0.001	0.021

Notes: Results derived from multiple linear regression analyses adjusted for age, age^2, sex, interactions between age and sex, interactions between age^2 and sex, site, smoking status, alcohol consumption, intracranial volume, hypertension, diabetes, and dyslipidemia.

Abbreviations: β, Standardized beta; SE, Standard error; T, T-statistic; P, P-value; R, Partial correlation coefficient; LCI, Lower confidence interval; UCI, Upper confidence interval

Table S16: General cognitive performance regression analyses

	β	SE	T	P	R	LCI	UCI
**Cardiometabolic variables:**							
**Body mass index**	-0.045	0.009	-4.855	$1.21e^{-06}$	-0.032	-0.045	-0.019
**Waist circumference**	-0.033	0.011	-3.105	$1.91e^{-03}$	-0.020	-0.033	-0.007
**Systolic blood pressure**	-0.040	0.010	-4.056	$5.00e^{-05}$	-0.027	-0.039	-0.014
**Diastolic blood pressure**	-0.038	0.009	-4.096	$4.22e^{-05}$	-0.027	-0.040	-0.014
**Pulse pressure**	-0.024	0.010	-2.395	$1.66e^{-02}$	-0.016	-0.028	-0.003
**C-reactive protein**	-0.039	0.016	-2.507	$1.22e^{-02}$	-0.016	-0.029	-0.004
**Glycated hemoglobin**	-0.053	0.009	-5.727	$1.03e^{-08}$	-0.037	-0.050	-0.025
**High-density lipoprotein cholesterol**	0.020	0.010	1.931	$5.34e^{-02}$	0.013	-0.000	0.025
**Low-density lipoprotein cholesterol**	0.006	0.009	0.618	$5.36e^{-01}$	0.004	-0.009	0.017
**Total cholesterol**	0.007	0.009	0.698	$4.85e^{-01}$	0.005	-0.008	0.017
**Triglycerides**	-0.093	0.031	-2.985	$2.84e^{-03}$	-0.020	-0.032	-0.007
**Cardiometabolic principal components:**							
**Principal component 1**	-0.033	0.006	-5.268	$1.39e^{-07}$	-0.034	-0.047	-0.022
**Principal component 2**	-0.013	0.007	-1.939	$5.25e^{-02}$	-0.013	-0.026	0.000
**Principal component 3**	-0.004	0.008	-0.551	$5.82e^{-01}$	-0.004	-0.016	0.009
**Cardiometabolic principal components without imputed data:**							
**Principal component 1**	-0.034	0.007	-5.138	$2.80e^{-07}$	-0.035	-0.048	-0.022
**Principal component 2**	0.011	0.007	1.511	$1.31e^{-01}$	0.010	-0.003	0.023
**Principal component 3**	-0.008	0.008	-0.984	$3.25e^{-01}$	-0.007	-0.020	0.006
**Liver fat variables:**							
**Liver fat**	-0.090	0.017	-5.244	$1.59e^{-07}$	-0.034	-0.047	-0.021
**Nonalcoholic fatty liver disease**	-0.099	0.026	-3.836	$1.25e^{-04}$	-0.025	-0.038	-0.012
**Metabolic dysfunction-associated fatty liver disease**	-0.109	0.022	-4.891	$1.01e^{-06}$	-0.032	-0.045	-0.019
**Metabolic dysfunction-associated steatotic liver disease**	-0.099	0.026	-3.797	$1.47e^{-04}$	-0.025	-0.038	-0.012
**White matter hyperintensities:**							
**White matter hyperintensities**	-0.116	0.011	-10.905	$1.27e^{-27}$	-0.071	-0.084	-0.058

Notes: Results derived from multiple linear regression analyses adjusted for age, age^2, sex, interactions between age and sex, interactions between age^2 and sex, site, smoking status, alcohol consumption, intracranial volume, and education.

Abbreviations: β, Standardized beta; SE, Standard error; T, T-statistic; P, P-value; R, Partial correlation coefficient; LCI, Lower confidence interval; UCI, Upper confidence interval

Table S17: Sex-interactions in general cognitive performance regression analyses

	β	SE	T	P	R	LCI	UCI
**Cardiometabolic variables:**							
**Body mass index**	-0.062	0.019	-3.242	$1.19e^{-03}$	-0.021	-0.034	-0.008
**Waist circumference**	-0.031	0.022	-1.431	$1.52e^{-01}$	-0.009	-0.022	0.003
**Systolic blood pressure**	-0.010	0.020	-0.500	$6.17e^{-01}$	-0.003	-0.016	0.010
**Diastolic blood pressure**	-0.009	0.018	-0.476	$6.34e^{-01}$	-0.003	-0.016	0.010
**Pulse pressure**	-0.002	0.020	-0.114	$9.09e^{-01}$	-0.001	-0.014	0.012
**C-reactive protein**	-0.003	0.031	-0.100	$9.20e^{-01}$	-0.001	-0.013	0.012
**Glycated hemoglobin**	-0.019	0.019	-1.037	$3.00e^{-01}$	-0.007	-0.020	0.006
**High-density lipoprotein cholesterol**	0.010	0.021	0.490	$6.24e^{-01}$	0.003	-0.010	0.016
**Low-density lipoprotein cholesterol**	0.025	0.019	1.358	$1.74e^{-01}$	0.009	-0.004	0.022
**Total cholesterol**	0.024	0.019	1.267	$2.05e^{-01}$	0.008	-0.005	0.021
**Triglycerides**	0.054	0.063	0.865	$3.87e^{-01}$	0.006	-0.007	0.018
**Cardiometabolic principal components:**							
**Principal component 1**	-0.013	0.013	-1.035	$3.01e^{-01}$	-0.007	-0.020	0.006
**Principal component 2**	-0.020	0.014	-1.431	$1.52e^{-01}$	-0.009	-0.022	0.003
**Principal component 3**	0.003	0.015	0.196	$8.45e^{-01}$	0.001	-0.012	0.014
**Cardiometabolic principal components without imputed data:**							
**Principal component 1**	-0.010	0.013	-0.789	$4.30e^{-01}$	-0.005	-0.018	0.007
**Principal component 2**	0.017	0.015	1.195	$2.32e^{-01}$	0.008	-0.005	0.021
**Principal component 3**	0.008	0.016	0.525	$6.00e^{-01}$	0.004	-0.009	0.016
**Liver fat variables:**							
**Liver fat**	0.036	0.034	1.069	$2.85e^{-01}$	0.007	-0.006	0.020
**Nonalcoholic fatty liver disease**	0.036	0.050	0.710	$4.78e^{-01}$	0.005	-0.008	0.017
**Metabolic dysfunction-associated fatty liver disease**	-0.023	0.045	-0.513	$6.08e^{-01}$	-0.003	-0.016	0.009
**Metabolic dysfunction-associated steatotic liver disease**	0.022	0.051	0.426	$6.70e^{-01}$	0.003	-0.010	0.016
**White matter hyperintensities:**							
**White matter hyperintensities**	-0.027	0.021	-1.299	$1.94e^{-01}$	-0.009	-0.021	0.004

Notes: Results derived from multiple linear regression analyses adjusted for age, age^2, sex, interactions between age and sex, interactions between age^2 and sex, site, smoking status, alcohol consumption, intracranial volume, and education.

Abbreviations: β, Standardized beta; SE, Standard error; T, T-statistic; P, P-value; R, Partial correlation coefficient; LCI, Lower confidence interval; UCI, Upper confidence interval

Table S18: General cognitive performance regression analyses adjusted for cardiometabolic diagnoses

	β	SE	T	P	R	LCI	UCI
**Cardiometabolic variables:**							
**Body mass index**	-0.030	0.009	-3.191	$1.42e^{-03}$	-0.021	-0.034	-0.008
**Waist circumference**	-0.016	0.011	-1.417	$1.57e^{-01}$	-0.009	-0.022	0.004
**Systolic blood pressure**	-0.031	0.010	-3.106	$1.90e^{-03}$	-0.020	-0.033	-0.008
**Diastolic blood pressure**	-0.031	0.009	-3.303	$9.60e^{-04}$	-0.022	-0.034	-0.009
**Pulse pressure**	-0.017	0.010	-1.677	$9.35e^{-02}$	-0.011	-0.024	0.002
**C-reactive protein**	-0.029	0.016	-1.872	$6.13e^{-02}$	-0.012	-0.025	0.001
**Glycated hemoglobin**	-0.040	0.011	-3.821	$1.33e^{-04}$	-0.025	-0.038	-0.012
**High-density lipoprotein cholesterol**	0.008	0.010	0.776	$4.38e^{-01}$	0.005	-0.008	0.018
**Low-density lipoprotein cholesterol**	-0.014	0.010	-1.500	$1.34e^{-01}$	-0.010	-0.023	0.003
**Total cholesterol**	-0.014	0.010	-1.480	$1.39e^{-01}$	-0.010	-0.023	0.003
**Triglycerides**	-0.068	0.031	-2.187	$2.88e^{-02}$	-0.014	-0.027	-0.001
**Cardiometabolic principal components:**							
**Principal component 1**	-0.026	0.006	-4.093	$4.27e^{-05}$	-0.027	-0.040	-0.014
**Principal component 2**	0.005	0.007	0.691	$4.90e^{-01}$	0.005	-0.008	0.017
**Principal component 3**	-0.000	0.008	-0.050	$9.60e^{-01}$	-0.000	-0.013	0.012
**Cardiometabolic principal components without imputed data:**							
**Principal component 1**	-0.028	0.007	-4.229	$2.36e^{-05}$	-0.029	-0.041	-0.016
**Principal component 2**	-0.006	0.008	-0.789	$4.30e^{-01}$	-0.005	-0.018	0.007
**Principal component 3**	-0.004	0.008	-0.541	$5.88e^{-01}$	-0.004	-0.016	0.009
**Liver fat variables:**							
**Liver fat**	-0.072	0.017	-4.195	$2.73e^{-05}$	-0.027	-0.040	-0.015
**Nonalcoholic fatty liver disease**	-0.077	0.026	-2.968	$3.00e^{-03}$	-0.019	-0.032	-0.007
**Metabolic dysfunction-associated fatty liver disease**	-0.084	0.023	-3.726	$1.95e^{-04}$	-0.024	-0.037	-0.012
**Metabolic dysfunction-associated steatotic liver disease**	-0.076	0.026	-2.889	$3.87e^{-03}$	-0.019	-0.032	-0.006
**White matter hyperintensities:**							
**White matter hyperintensities**	-0.108	0.011	-10.082	$7.45e^{-24}$	-0.066	-0.079	-0.053

Notes: Results derived from multiple linear regression analyses adjusted for age, age^2, sex, interactions between age and sex, interactions between age^2 and sex, site, smoking status, alcohol consumption, intracranial volume, education, hypertension, diabetes, and dyslipidemia.

Abbreviations: β, Standardized beta; SE, Standard error; T, T-statistic; P, P-value; R, Partial correlation coefficient; LCI, Lower confidence interval; UCI, Upper confidence interval

Table S19: Sex-interactions in general cognitive performance regression analyses adjusted for cardiometabolic diagnoses

	β	SE	T	P	R	LCI	UCI
**Cardiometabolic variables:**							
**Body mass index**	-0.053	0.019	-2.763	$5.74e^{-03}$	-0.018	-0.031	-0.005
**Waist circumference**	-0.023	0.022	-1.076	$2.82e^{-01}$	-0.007	-0.020	0.006
**Systolic blood pressure**	-0.010	0.019	-0.535	$5.92e^{-01}$	-0.004	-0.016	0.009
**Diastolic blood pressure**	-0.010	0.018	-0.541	$5.88e^{-01}$	-0.004	-0.016	0.009
**Pulse pressure**	-0.003	0.020	-0.153	$8.79e^{-01}$	-0.001	-0.014	0.012
**C-reactive protein**	-0.008	0.031	-0.259	$7.96e^{-01}$	-0.002	-0.015	0.011
**Glycated hemoglobin**	-0.016	0.019	-0.865	$3.87e^{-01}$	-0.006	-0.018	0.007
**High-density lipoprotein cholesterol**	0.003	0.021	0.144	$8.85e^{-01}$	0.001	-0.012	0.014
**Low-density lipoprotein cholesterol**	0.007	0.019	0.387	$6.99e^{-01}$	0.003	-0.010	0.015
**Total cholesterol**	0.007	0.019	0.371	$7.11e^{-01}$	0.002	-0.010	0.015
**Triglycerides**	0.041	0.063	0.647	$5.18e^{-01}$	0.004	-0.009	0.017
**Cardiometabolic principal components:**							
**Principal component 1**	-0.013	0.012	-1.074	$2.83e^{-01}$	-0.007	-0.020	0.006
**Principal component 2**	-0.008	0.014	-0.598	$5.50e^{-01}$	-0.004	-0.017	0.009
**Principal component 3**	0.007	0.015	0.466	$6.41e^{-01}$	0.003	-0.010	0.016
**Cardiometabolic principal components without imputed data:**							
**Principal component 1**	-0.011	0.013	-0.845	$3.98e^{-01}$	-0.006	-0.019	0.007
**Principal component 2**	0.006	0.015	0.434	$6.64e^{-01}$	0.003	-0.010	0.016
**Principal component 3**	0.012	0.016	0.742	$4.58e^{-01}$	0.005	-0.008	0.018
**Liver fat variables:**							
**Liver fat**	0.037	0.034	1.107	$2.68e^{-01}$	0.007	-0.006	0.020
**Nonalcoholic fatty liver disease**	0.035	0.050	0.688	$4.92e^{-01}$	0.005	-0.008	0.017
**Metabolic dysfunction-associated fatty liver disease**	-0.024	0.045	-0.529	$5.97e^{-01}$	-0.003	-0.016	0.009
**Metabolic dysfunction-associated steatotic liver disease**	0.020	0.051	0.395	$6.93e^{-01}$	0.003	-0.010	0.015
**White matter hyperintensities:**							
**White matter hyperintensities**	-0.015	0.021	-0.738	$4.61e^{-01}$	-0.005	-0.018	0.008

Notes: Results derived from multiple linear regression analyses adjusted for age, age^2, sex, interactions between age and sex, interactions between age^2 and sex, site, smoking status, alcohol consumption, intracranial volume, education, hypertension, diabetes, and dyslipidemia.

Abbreviations: β, Standardized beta; SE, Standard error; T, T-statistic; P, P-value; R, Partial correlation coefficient; LCI, Lower confidence interval; UCI, Upper confidence interval

Table S20 General cognitive performance regression analyses without imputation

	β	SE	T	P	R	LCI	UCI
**Cardiometabolic variables:**							
**Body mass index**	0.034	0.010	3.387	$7.08e^{-04}$	0.025	0.010	0.039
**Waist circumference**	0.022	0.012	1.893	$5.84e^{-02}$	0.014	-0.000	0.028
**Systolic blood pressure**	0.039	0.011	3.696	$2.20e^{-04}$	0.027	0.013	0.041
**Diastolic blood pressure**	0.036	0.010	3.636	$2.77e^{-04}$	0.026	0.012	0.041
**Pulse pressure**	0.024	0.011	2.252	$2.43e^{-02}$	0.016	0.002	0.031
**C-reactive protein**	0.036	0.017	2.162	$3.06e^{-02}$	0.016	0.001	0.030
**Glycated hemoglobin**	0.042	0.010	4.056	$5.00e^{-05}$	0.029	0.015	0.044
**High-density lipoprotein cholesterol**	-0.009	0.011	-0.818	$4.13e^{-01}$	-0.006	-0.020	0.008
**Low-density lipoprotein cholesterol**	0.005	0.010	0.518	$6.04e^{-01}$	0.004	-0.010	0.018
**Total cholesterol**	0.007	0.010	0.672	$5.02e^{-01}$	0.005	-0.009	0.019
**Triglycerides**	0.094	0.034	2.799	$5.13e^{-03}$	0.020	0.006	0.034
**Cardiometabolic principal components:**							
**Principal component 1**	0.030	0.007	4.550	$5.40e^{-06}$	0.033	0.019	0.047
**Principal component 2**	0.001	0.007	0.101	$9.20e^{-01}$	0.001	-0.013	0.015
**Principal component 3**	0.007	0.008	0.794	$4.27e^{-01}$	0.006	-0.008	0.020
**Cardiometabolic principal components without imputed data:**							
**Principal component 1**	0.033	0.007	4.684	$2.83e^{-06}$	0.035	0.021	0.049
**Principal component 2**	0.000	0.008	0.009	$9.93e^{-01}$	0.000	-0.014	0.014
**Principal component 3**	0.010	0.009	1.106	$2.69e^{-01}$	0.008	-0.006	0.022
**Liver fat variables:**							
**Liver fat**	0.076	0.018	4.106	$4.04e^{-05}$	0.030	0.016	0.044
**Nonalcoholic fatty liver disease**	0.084	0.028	2.997	$2.73e^{-03}$	0.022	0.008	0.036
**Metabolic dysfunction-associated fatty liver disease**	0.078	0.024	3.206	$1.35e^{-03}$	0.023	0.009	0.037
**Metabolic dysfunction-associated steatotic liver disease**	0.078	0.028	2.761	$5.76e^{-03}$	0.020	0.006	0.034
**White matter hyperintensities:**							
**White matter hyperintensities**	0.112	0.011	9.731	$2.52e^{-22}$	0.070	0.056	0.084

Notes: Results derived from multiple linear regression analyses adjusted for age, age^2, sex, interactions between age and sex, interactions between age^2 and sex, site, smoking status, alcohol consumption, intracranial volume, and education.

Abbreviations: β, Standardized beta; SE, Standard error; T, T-statistic; P, P-value; R, Partial correlation coefficient; LCI, Lower confidence interval; UCI, Upper confidence interval

Table S21 Sex-interactions in general cognitive performance regression analyses without imputation

	β	SE	T	P	R	LCI	UCI
**Cardiometabolic variables:**							
**Body mass index**	0.055	0.021	2.688	$7.18e^{-03}$	0.019	0.005	0.034
**Waist circumference**	0.038	0.023	1.633	$1.03e^{-01}$	0.012	-0.002	0.026
**Systolic blood pressure**	0.017	0.021	0.788	$4.31e^{-01}$	0.006	-0.008	0.020
**Diastolic blood pressure**	0.020	0.020	0.993	$3.21e^{-01}$	0.007	-0.007	0.021
**Pulse pressure**	0.002	0.022	0.081	$9.36e^{-01}$	0.001	-0.014	0.015
**C-reactive protein**	0.038	0.034	1.127	$2.60e^{-01}$	0.008	-0.006	0.022
**Glycated hemoglobin**	0.014	0.021	0.700	$4.84e^{-01}$	0.005	-0.009	0.019
**High-density lipoprotein cholesterol**	-0.022	0.023	-0.974	$3.30e^{-01}$	-0.007	-0.021	0.007
**Low-density lipoprotein cholesterol**	-0.003	0.020	-0.167	$8.67e^{-01}$	-0.001	-0.015	0.013
**Total cholesterol**	-0.009	0.020	-0.435	$6.64e^{-01}$	-0.003	-0.017	0.011
**Triglycerides**	-0.067	0.068	-0.990	$3.22e^{-01}$	-0.007	-0.021	0.007
**Cardiometabolic principal components:**							
**Principal component 1**	0.023	0.013	1.741	$8.18e^{-02}$	0.013	-0.002	0.027
**Principal component 2**	0.013	0.015	0.862	$3.89e^{-01}$	0.006	-0.008	0.020
**Principal component 3**	-0.010	0.017	-0.586	$5.58e^{-01}$	-0.004	-0.018	0.010
**Cardiometabolic principal components without imputed data:**							
**Principal component 1**	0.024	0.014	1.720	$8.55e^{-02}$	0.013	-0.001	0.027
**Principal component 2**	-0.011	0.016	-0.706	$4.80e^{-01}$	-0.005	-0.019	0.009
**Principal component 3**	-0.016	0.018	-0.929	$3.53e^{-01}$	-0.007	-0.021	0.007
**Liver fat variables:**							
**Liver fat**	-0.039	0.037	-1.060	$2.89e^{-01}$	-0.008	-0.022	0.007
**Nonalcoholic fatty liver disease**	-0.029	0.055	-0.526	$5.99e^{-01}$	-0.004	-0.018	0.010
**Metabolic dysfunction-associated fatty liver disease**	0.025	0.049	0.509	$6.11e^{-01}$	0.004	-0.011	0.018
**Metabolic dysfunction-associated steatotic liver disease**	-0.008	0.055	-0.140	$8.88e^{-01}$	-0.001	-0.015	0.013
**White matter hyperintensities:**							
**White matter hyperintensities**	0.014	0.023	0.622	$5.34e^{-01}$	0.005	-0.010	0.019

Notes: Results derived from multiple linear regression analyses adjusted for age, age^2, sex, interactions between age and sex, interactions between age^2 and sex, site, smoking status, alcohol consumption, intracranial volume, and education.

Abbreviations: β, Standardized beta; SE, Standard error; T, T-statistic; P, P-value; R, Partial correlation coefficient; LCI, Lower confidence interval; UCI, Upper confidence interval

Table S22 Cognitive test regression analyses

	β	SE	T	P	R	LCI	UCI
**Numeric memory – Cardiometabolic risk factors:**							
**Body mass index**	-0.053	0.006	-8.158	$3.56e^{-16}$	-0.053	-0.066	-0.040
**Waist circumference**	-0.049	0.007	-6.526	$6.88e^{-11}$	-0.042	-0.055	-0.030
**Systolic blood pressure**	-0.037	0.007	-5.404	$6.60e^{-08}$	-0.035	-0.048	-0.022
**Diastolic blood pressure**	-0.037	0.006	-5.775	$7.81e^{-09}$	-0.038	-0.050	-0.025
**Pulse pressure**	-0.020	0.007	-2.917	$3.54e^{-03}$	-0.019	-0.032	-0.006
**C-reactive protein**	-0.038	0.011	-3.456	$5.49e^{-04}$	-0.022	-0.035	-0.010
**Glycated hemoglobin**	-0.024	0.006	-3.658	$2.55e^{-04}$	-0.024	-0.037	-0.011
**High-density lipoprotein cholesterol**	0.031	0.007	4.315	$1.61e^{-05}$	0.028	0.015	0.041
**Low-density lipoprotein cholesterol**	-0.005	0.006	-0.851	$3.95e^{-01}$	-0.006	-0.018	0.007
**Total cholesterol**	-0.001	0.007	-0.153	$8.79e^{-01}$	-0.001	-0.014	0.012
**Triglycerides**	-0.117	0.022	-5.384	$7.34e^{-08}$	-0.035	-0.048	-0.022
**Numeric memory – Cardiometabolic principal components:**							
**Principal component 1**	-0.036	0.004	-8.409	$4.38e^{-17}$	-0.055	-0.067	-0.042
**Principal component 2**	-0.011	0.005	-2.177	$2.95e^{-02}$	-0.014	-0.027	-0.001
**Principal component 3**	0.007	0.005	1.385	$1.66e^{-01}$	0.009	-0.004	0.022
**Numeric memory – Cardiometabolic principal components without imputed data:**							
**Principal component 1**	-0.037	0.005	-8.032	$1.01e^{-15}$	-0.054	-0.067	-0.041
**Principal component 2**	0.011	0.005	2.121	$3.39e^{-02}$	0.014	0.002	0.027
**Principal component 3**	0.004	0.006	0.764	$4.45e^{-01}$	0.005	-0.008	0.018
**Numeric memory – Liver fat variables:**							
**Liver fat**	-0.085	0.012	-7.090	$1.38e^{-12}$	-0.046	-0.059	-0.033
**Nonalcoholic fatty liver disease**	-0.098	0.018	-5.474	$4.46e^{-08}$	-0.036	-0.048	-0.023
**Metabolic dysfunction-associated fatty liver disease**	-0.096	0.016	-6.131	$8.88e^{-10}$	-0.040	-0.053	-0.027
**Metabolic dysfunction-associated steatotic liver disease**	-0.099	0.018	-5.428	$5.75e^{-08}$	-0.035	-0.048	-0.023
**Numeric memory – White matter hyperintensities:**							
**White matter hyperintensities**	-0.043	0.007	-5.766	$8.20e^{-09}$	-0.037	-0.050	-0.025
**Fluid intelligence – Cardiometabolic risk factors:**							
**Body mass index**	-0.002	0.005	-0.375	$7.08e^{-01}$	-0.002	-0.013	0.009
**Waist circumference**	0.012	0.006	1.929	$5.37e^{-02}$	0.011	-0.000	0.022
**Systolic blood pressure**	-0.011	0.006	-1.978	$4.80e^{-02}$	-0.011	-0.023	-0.000
**Diastolic blood pressure**	-0.019	0.006	-3.417	$6.35e^{-04}$	-0.020	-0.031	-0.008
**Pulse pressure**	0.000	0.006	0.050	$9.60e^{-01}$	0.000	-0.011	0.012
**C-reactive protein**	-0.012	0.009	-1.254	$2.10e^{-01}$	-0.007	-0.019	0.004
**Glycated hemoglobin**	-0.010	0.006	-1.755	$7.92e^{-02}$	-0.010	-0.021	0.001

	β	SE	T	P	R	LCI	UCI
**High-density lipoprotein cholesterol**	0.005	0.006	0.890	3.73e-01	0.005	-0.006	0.016
**Low-density lipoprotein cholesterol**	0.001	0.005	0.226	8.21e-01	0.001	-0.010	0.013
**Total cholesterol**	0.003	0.006	0.455	6.49e-01	0.003	-0.009	0.014
**Triglycerides**	0.013	0.018	0.708	4.79e-01	0.004	-0.007	0.015
**Fluid intelligence – Cardiometabolic principal components:**							
**Principal component 1**	-0.005	0.004	-1.421	1.55e-01	-0.008	-0.020	0.003
**Principal component 2**	-0.002	0.004	-0.407	6.84e-01	-0.002	-0.014	0.009
**Principal component 3**	-0.007	0.005	-1.495	1.35e-01	-0.009	-0.020	0.003
**Fluid intelligence – Cardiometabolic principal components without imputed data:**							
**Principal component 1**	-0.008	0.004	-1.934	5.31e-02	-0.012	-0.023	-0.000
**Principal component 2**	0.004	0.004	0.897	3.70e-01	0.005	-0.006	0.017
**Principal component 3**	-0.006	0.005	-1.350	1.77e-01	-0.008	-0.019	0.003
**Fluid intelligence – Liver fat variables:**							
**Liver fat**	-0.035	0.010	-3.559	3.73e-04	-0.021	-0.032	-0.009
**Nonalcoholic fatty liver disease**	-0.022	0.015	-1.435	1.51e-01	-0.008	-0.020	0.003
**Metabolic dysfunction-associated fatty liver disease**	-0.029	0.013	-2.172	2.98e-02	-0.013	-0.024	-0.001
**Metabolic dysfunction-associated steatotic liver disease**	-0.020	0.016	-1.266	2.05e-01	-0.007	-0.019	0.004
**Fluid intelligence – White matter hyperintensities:**							
**White matter hyperintensities**	-0.048	0.006	-7.590	3.28e-14	-0.044	-0.055	-0.032
**Trail making test part B – Cardiometabolic risk factors:**							
**Body mass index**	0.002	0.002	0.674	5.00e-01	0.005	-0.009	0.019
**Waist circumference**	-0.002	0.003	-0.647	5.18e-01	-0.005	-0.018	0.009
**Systolic blood pressure**	0.002	0.002	0.627	5.31e-01	0.004	-0.009	0.018
**Diastolic blood pressure**	0.002	0.002	1.050	2.94e-01	0.007	-0.006	0.021
**Pulse pressure**	0.000	0.003	0.004	9.97e-01	0.000	-0.014	0.014
**C-reactive protein**	0.007	0.004	1.667	9.56e-02	0.012	-0.002	0.026
**Glycated hemoglobin**	0.009	0.002	3.864	1.12e-04	0.027	0.013	0.041
**High-density lipoprotein cholesterol**	0.001	0.003	0.395	6.93e-01	0.003	-0.011	0.017
**Low-density lipoprotein cholesterol**	-0.002	0.002	-0.732	4.64e-01	-0.005	-0.019	0.009
**Total cholesterol**	-0.001	0.002	-0.422	6.73e-01	-0.003	-0.017	0.011
**Triglycerides**	-0.006	0.008	-0.780	4.35e-01	-0.006	-0.019	0.008
**Trail making test part B – Cardiometabolic principal components:**							
**Principal component 1**	0.001	0.002	0.666	5.05e-01	0.005	-0.009	0.019
**Principal component 2**	0.001	0.002	0.320	7.49e-01	0.002	-0.012	0.016
**Principal component 3**	0.002	0.002	0.923	3.56e-01	0.007	-0.007	0.020
**Trail making test part B – Cardiometabolic principal components without imputed data:**							

	β	SE	T	P	R	LCI	UCI
**Principal component 1**	0.002	0.002	0.981	$3.27e^{-01}$	0.007	-0.007	0.021
**Principal component 2**	0.000	0.002	0.220	$8.26e^{-01}$	0.002	-0.012	0.015
**Principal component 3**	0.002	0.002	1.006	$3.14e^{-01}$	0.007	-0.006	0.021
**Trail making test part B – Liver fat variables:**							
**Liver fat**	0.002	0.004	0.557	$5.77e^{-01}$	0.004	-0.010	0.018
**Nonalcoholic fatty liver disease**	0.000	0.007	0.068	$9.46e^{-01}$	0.000	-0.013	0.014
**Metabolic dysfunction-associated fatty liver disease**	0.005	0.006	0.833	$4.05e^{-01}$	0.006	-0.008	0.020
**Metabolic dysfunction-associated steatotic liver disease**	0.002	0.007	0.319	$7.50e^{-01}$	0.002	-0.012	0.016
**Trail making test part B – White matter hyperintensities:**							
**White matter hyperintensities**	0.020	0.003	7.240	$4.65e^{-13}$	0.051	0.037	0.065
**Matrix test – Cardiometabolic risk factors:**							
**Body mass index**	-0.018	0.007	-2.699	$6.97e^{-03}$	-0.019	-0.033	-0.005
**Waist circumference**	-0.010	0.008	-1.275	$2.02e^{-01}$	-0.009	-0.023	0.005
**Systolic blood pressure**	-0.012	0.007	-1.693	$9.05e^{-02}$	-0.012	-0.026	0.002
**Diastolic blood pressure**	-0.011	0.007	-1.631	$1.03e^{-01}$	-0.011	-0.025	0.002
**Pulse pressure**	-0.008	0.007	-1.065	$2.87e^{-01}$	-0.007	-0.021	0.006
**C-reactive protein**	-0.008	0.011	-0.720	$4.72e^{-01}$	-0.005	-0.019	0.009
**Glycated hemoglobin**	-0.014	0.007	-2.062	$3.93e^{-02}$	-0.014	-0.028	-0.001
**High-density lipoprotein cholesterol**	-0.000	0.007	-0.016	$9.87e^{-01}$	-0.000	-0.014	0.014
**Low-density lipoprotein cholesterol**	0.003	0.007	0.523	$6.01e^{-01}$	0.004	-0.010	0.017
**Total cholesterol**	0.004	0.007	0.641	$5.22e^{-01}$	0.004	-0.009	0.018
**Triglycerides**	-0.006	0.023	-0.263	$7.93e^{-01}$	-0.002	-0.016	0.012
**Matrix test – Cardiometabolic principal components:**							
**Principal component 1**	-0.009	0.004	-2.076	$3.79e^{-02}$	-0.015	-0.028	-0.001
**Principal component 2**	-0.006	0.005	-1.222	$2.22e^{-01}$	-0.009	-0.022	0.005
**Principal component 3**	-0.003	0.006	-0.530	$5.96e^{-01}$	-0.004	-0.017	0.010
**Matrix test – Cardiometabolic principal components without imputed data:**							
**Principal component 1**	-0.010	0.005	-2.110	$3.49e^{-02}$	-0.015	-0.029	-0.002
**Principal component 2**	0.005	0.005	0.950	$3.42e^{-01}$	0.007	-0.007	0.021
**Principal component 3**	-0.006	0.006	-0.985	$3.25e^{-01}$	-0.007	-0.021	0.007
**Matrix test – Liver fat variables:**							
**Liver fat**	-0.052	0.012	-4.241	$2.23e^{-05}$	-0.030	-0.043	-0.016
**Nonalcoholic fatty liver disease**	-0.072	0.019	-3.866	$1.11e^{-04}$	-0.027	-0.041	-0.013
**Metabolic dysfunction-associated fatty liver disease**	-0.053	0.016	-3.296	$9.84e^{-04}$	-0.023	-0.037	-0.009
**Metabolic dysfunction-associated steatotic liver disease**	-0.071	0.019	-3.740	$1.84e^{-04}$	-0.026	-0.040	-0.012
**Matrix test – White matter hyperintensities:**							

	β	SE	T	P	R	LCI	UCI
**White matter hyperintensities**	-0.057	0.008	-7.428	$1.15e^{-13}$	-0.052	-0.066	-0.038
**Symbol digit substitution – Cardiometabolic risk factors:**							
**Body mass index**	-0.036	0.006	-5.627	$1.86e^{-08}$	-0.039	-0.053	-0.026
**Waist circumference**	-0.041	0.007	-5.468	$4.59e^{-08}$	-0.038	-0.052	-0.025
**Systolic blood pressure**	-0.009	0.007	-1.374	$1.69e^{-01}$	-0.010	-0.023	0.004
**Diastolic blood pressure**	-0.019	0.006	-2.968	$3.00e^{-03}$	-0.021	-0.034	-0.007
**Pulse pressure**	0.004	0.007	0.565	$5.72e^{-01}$	0.004	-0.010	0.018
**C-reactive protein**	-0.033	0.011	-3.052	$2.28e^{-03}$	-0.021	-0.035	-0.008
**Glycated hemoglobin**	-0.038	0.006	-5.941	$2.88e^{-09}$	-0.041	-0.055	-0.028
**High-density lipoprotein cholesterol**	0.015	0.007	2.169	$3.01e^{-02}$	0.015	0.001	0.029
**Low-density lipoprotein cholesterol**	0.002	0.006	0.303	$7.62e^{-01}$	0.002	-0.012	0.016
**Total cholesterol**	0.002	0.006	0.305	$7.60e^{-01}$	0.002	-0.012	0.016
**Triglycerides**	-0.078	0.022	-3.640	$2.74e^{-04}$	-0.025	-0.039	-0.012
**Symbol digit substitution – Cardiometabolic principal components:**							
**Principal component 1**	-0.019	0.004	-4.460	$8.24e^{-06}$	-0.031	-0.045	-0.017
**Principal component 2**	-0.011	0.005	-2.213	$2.69e^{-02}$	-0.015	-0.029	-0.002
**Principal component 3**	0.012	0.005	2.346	$1.90e^{-02}$	0.016	0.003	0.030
**Symbol digit substitution – Cardiometabolic principal components without imputed data:**							
**Principal component 1**	-0.018	0.005	-4.057	$4.99e^{-05}$	-0.029	-0.043	-0.016
**Principal component 2**	0.006	0.005	1.263	$2.07e^{-01}$	0.009	-0.005	0.023
**Principal component 3**	0.012	0.006	2.073	$3.82e^{-02}$	0.015	0.001	0.029
**Symbol digit substitution – Liver fat variables:**							
**Liver fat**	-0.052	0.012	-4.383	$1.17e^{-05}$	-0.031	-0.044	-0.017
**Nonalcoholic fatty liver disease**	-0.048	0.018	-2.709	$6.76e^{-03}$	-0.019	-0.033	-0.005
**Metabolic dysfunction-associated fatty liver disease**	-0.074	0.016	-4.767	$1.88e^{-06}$	-0.033	-0.047	-0.020
**Metabolic dysfunction-associated steatotic liver disease**	-0.049	0.018	-2.714	$6.66e^{-03}$	-0.019	-0.033	-0.005
**Symbol digit substitution – White matter hyperintensities:**							
**White matter hyperintensities**	-0.065	0.007	-8.699	$3.60e^{-18}$	-0.061	-0.074	-0.047
**Tower rearranging – Cardiometabolic risk factors:**							
**Body mass index**	0.029	0.007	4.226	$2.39e^{-05}$	0.030	0.016	0.043
**Waist circumference**	0.039	0.008	4.886	$1.03e^{-06}$	0.034	0.021	0.048
**Systolic blood pressure**	-0.000	0.007	-0.036	$9.72e^{-01}$	-0.000	-0.014	0.014
**Diastolic blood pressure**	0.012	0.007	1.702	$8.87e^{-02}$	0.012	-0.002	0.026
**Pulse pressure**	-0.012	0.008	-1.535	$1.25e^{-01}$	-0.011	-0.025	0.003
**C-reactive protein**	0.036	0.012	3.085	$2.04e^{-03}$	0.022	0.008	0.035
**Glycated hemoglobin**	-0.019	0.007	-2.723	$6.48e^{-03}$	-0.019	-0.033	-0.005

	β	SE	T	P	R	LCI	UCI
**High-density lipoprotein cholesterol**	-0.017	0.008	-2.202	$2.77e^{-02}$	-0.015	-0.029	-0.002
**Low-density lipoprotein cholesterol**	0.006	0.007	0.916	$3.60e^{-01}$	0.006	-0.007	0.020
**Total cholesterol**	0.002	0.007	0.289	$7.72e^{-01}$	0.002	-0.012	0.016
**Triglycerides**	0.036	0.024	1.542	$1.23e^{-01}$	0.011	-0.003	0.025
**Tower rearranging – Cardiometabolic principal components:**							
**Principal component 1**	0.014	0.005	2.937	$3.32e^{-03}$	0.021	0.007	0.034
**Principal component 2**	0.008	0.005	1.601	$1.09e^{-01}$	0.011	-0.003	0.025
**Principal component 3**	-0.020	0.006	-3.386	$7.10e^{-04}$	-0.024	-0.038	-0.010
**Tower rearranging – Cardiometabolic principal components without imputed data:**							
**Principal component 1**	0.013	0.005	2.683	$7.31e^{-03}$	0.020	0.006	0.033
**Principal component 2**	-0.010	0.005	-1.777	$7.56e^{-02}$	-0.013	-0.027	0.001
**Principal component 3**	-0.023	0.006	-3.769	$1.64e^{-04}$	-0.027	-0.041	-0.014
**Tower rearranging – Liver fat variables:**							
**Liver fat**	0.042	0.013	3.246	$1.17e^{-03}$	0.023	0.009	0.037
**Nonalcoholic fatty liver disease**	0.039	0.019	2.003	$4.52e^{-02}$	0.014	0.000	0.028
**Metabolic dysfunction-associated fatty liver disease**	0.051	0.017	3.016	$2.56e^{-03}$	0.021	0.007	0.035
**Metabolic dysfunction-associated steatotic liver disease**	0.043	0.020	2.161	$3.07e^{-02}$	0.015	0.001	0.029
**Tower rearranging – White matter hyperintensities:**							
**White matter hyperintensities**	-0.039	0.008	-4.871	$1.12e^{-06}$	-0.034	-0.048	-0.020
**Paired associate learning – Cardiometabolic risk factors:**							
**Body mass index**	-0.053	0.006	-8.391	$5.09e^{-17}$	-0.055	-0.067	-0.042
**Waist circumference**	-0.055	0.007	-7.555	$4.33e^{-14}$	-0.049	-0.062	-0.036
**Systolic blood pressure**	-0.015	0.007	-2.219	$2.65e^{-02}$	-0.014	-0.027	-0.002
**Diastolic blood pressure**	-0.018	0.006	-2.878	$4.01e^{-03}$	-0.019	-0.032	-0.006
**Pulse pressure**	-0.005	0.007	-0.763	$4.46e^{-01}$	-0.005	-0.018	0.008
**C-reactive protein**	-0.041	0.011	-3.825	$1.31e^{-04}$	-0.025	-0.038	-0.012
**Glycated hemoglobin**	-0.025	0.006	-4.001	$6.32e^{-05}$	-0.026	-0.039	-0.013
**High-density lipoprotein cholesterol**	0.020	0.007	2.902	$3.71e^{-03}$	0.019	0.006	0.032
**Low-density lipoprotein cholesterol**	-0.005	0.006	-0.797	$4.26e^{-01}$	-0.005	-0.018	0.008
**Total cholesterol**	-0.003	0.006	-0.537	$5.91e^{-01}$	-0.004	-0.016	0.009
**Triglycerides**	-0.098	0.021	-4.618	$3.89e^{-06}$	-0.030	-0.043	-0.017
**Paired associate learning – Cardiometabolic principal components:**							
**Principal component 1**	-0.028	0.004	-6.680	$2.45e^{-11}$	-0.044	-0.056	-0.031
**Principal component 2**	-0.011	0.005	-2.282	$2.25e^{-02}$	-0.015	-0.028	-0.002
**Principal component 3**	0.020	0.005	3.826	$1.31e^{-04}$	0.025	0.012	0.038
**Paired associate learning – Cardiometabolic principal components without imputed data:**							

	β	SE	T	P	R	LCI	UCI
**Principal component 1**	-0.030	0.004	-6.603	$4.13e^{-11}$	-0.045	-0.057	-0.032
**Principal component 2**	0.008	0.005	1.693	$9.05e^{-02}$	0.011	-0.001	0.024
**Principal component 3**	0.021	0.006	3.763	$1.68e^{-04}$	0.025	0.013	0.038
**Paired associate learning – Liver fat variables:**							
**Liver fat**	-0.075	0.012	-6.378	$1.83e^{-10}$	-0.042	-0.054	-0.029
**Nonalcoholic fatty liver disease**	-0.083	0.018	-4.726	$2.30e^{-06}$	-0.031	-0.044	-0.018
**Metabolic dysfunction-associated fatty liver disease**	-0.101	0.015	-6.590	$4.48e^{-11}$	-0.043	-0.056	-0.030
**Metabolic dysfunction-associated steatotic liver disease**	-0.090	0.018	-5.050	$4.45e^{-07}$	-0.033	-0.046	-0.020
**Paired associate learning – White matter hyperintensities:**							
**White matter hyperintensities**	-0.052	0.007	-7.091	$1.37e^{-12}$	-0.046	-0.059	-0.033
**Pairs matching – Cardiometabolic risk factors:**							
**Body mass index**	-0.020	0.004	-5.447	$5.17e^{-08}$	-0.031	-0.042	-0.020
**Waist circumference**	-0.024	0.004	-5.765	$8.22e^{-09}$	-0.033	-0.044	-0.022
**Systolic blood pressure**	-0.004	0.004	-1.072	$2.84e^{-01}$	-0.006	-0.017	0.005
**Diastolic blood pressure**	-0.006	0.004	-1.676	$9.38e^{-02}$	-0.010	-0.021	0.002
**Pulse pressure**	-0.000	0.004	-0.126	$9.00e^{-01}$	-0.001	-0.012	0.010
**C-reactive protein**	-0.012	0.006	-2.046	$4.08e^{-02}$	-0.012	-0.023	-0.000
**Glycated hemoglobin**	0.003	0.004	0.688	$4.92e^{-01}$	0.004	-0.007	0.015
**High-density lipoprotein cholesterol**	0.007	0.004	1.833	$6.68e^{-02}$	0.010	-0.001	0.022
**Low-density lipoprotein cholesterol**	-0.014	0.004	-3.759	$1.71e^{-04}$	-0.021	-0.033	-0.010
**Total cholesterol**	-0.011	0.004	-3.090	$2.00e^{-03}$	-0.018	-0.029	-0.006
**Triglycerides**	-0.029	0.012	-2.340	$1.93e^{-02}$	-0.013	-0.025	-0.002
**Pairs matching – Cardiometabolic principal components:**							
**Principal component 1**	-0.012	0.002	-4.758	$1.97e^{-06}$	-0.027	-0.038	-0.016
**Principal component 2**	0.003	0.003	1.078	$2.81e^{-01}$	0.006	-0.005	0.017
**Principal component 3**	0.013	0.003	4.420	$9.92e^{-06}$	0.025	0.014	0.036
**Pairs matching – Cardiometabolic principal components without imputed data:**							
**Principal component 1**	-0.011	0.003	-4.441	$9.00e^{-06}$	-0.026	-0.038	-0.015
**Principal component 2**	-0.002	0.003	-0.609	$5.42e^{-01}$	-0.004	-0.015	0.008
**Principal component 3**	0.012	0.003	3.865	$1.11e^{-04}$	0.023	0.012	0.034
**Pairs matching – Liver fat variables:**							
**Liver fat**	-0.020	0.007	-3.110	$1.87e^{-03}$	-0.018	-0.029	-0.007
**Nonalcoholic fatty liver disease**	-0.034	0.010	-3.387	$7.07e^{-04}$	-0.019	-0.031	-0.008
**Metabolic dysfunction-associated fatty liver disease**	-0.032	0.009	-3.614	$3.02e^{-04}$	-0.021	-0.032	-0.009
**Metabolic dysfunction-associated steatotic liver disease**	-0.038	0.010	-3.713	$2.05e^{-04}$	-0.021	-0.032	-0.010
**Pairs matching – White matter hyperintensities:**							

	β	SE	T	P	R	LCI	UCI
**White matter hyperintensities**	0.014	0.004	3.259	$1.12e^{-03}$	0.019	0.007	0.030

Notes: Results derived from multiple linear regression analyses adjusted for age, age^2, sex, interactions between age and sex, interactions between age^2 and sex, site, smoking status, alcohol consumption, intracranial volume, and education.

Abbreviations: β, Standardized beta; SE, Standard error; T, T-statistic; P, P-value; R, Partial correlation coefficient; LCI, Lower confidence interval; UCI, Upper confidence interval

Table S23 Sex-interactions in cognitive test regression analyses

	β	SE	T	P	R	LCI	UCI
**Numeric memory – Cardiometabolic risk factors:**							
**Body mass index**	-0.028	0.013	-2.124	$3.37e^{-02}$	-0.014	-0.027	-0.001
**Waist circumference**	-0.016	0.015	-1.081	$2.80e^{-01}$	-0.007	-0.020	0.006
**Systolic blood pressure**	-0.009	0.014	-0.665	$5.06e^{-01}$	-0.004	-0.017	0.008
**Diastolic blood pressure**	-0.013	0.013	-1.046	$2.96e^{-01}$	-0.007	-0.020	0.006
**Pulse pressure**	0.003	0.014	0.232	$8.17e^{-01}$	0.002	-0.011	0.014
**C-reactive protein**	-0.025	0.022	-1.154	$2.48e^{-01}$	-0.008	-0.020	0.005
**Glycated hemoglobin**	-0.014	0.013	-1.098	$2.72e^{-01}$	-0.007	-0.020	0.006
**High-density lipoprotein cholesterol**	-0.001	0.015	-0.041	$9.67e^{-01}$	-0.000	-0.013	0.012
**Low-density lipoprotein cholesterol**	-0.015	0.013	-1.182	$2.37e^{-01}$	-0.008	-0.020	0.005
**Total cholesterol**	-0.020	0.013	-1.488	$1.37e^{-01}$	-0.010	-0.022	0.003
**Triglycerides**	0.021	0.044	0.490	$6.24e^{-01}$	0.003	-0.010	0.016
**Numeric memory – Cardiometabolic principal components:**							
**Principal component 1**	-0.012	0.009	-1.384	$1.66e^{-01}$	-0.009	-0.022	0.004
**Principal component 2**	0.012	0.010	1.276	$2.02e^{-01}$	0.008	-0.004	0.021
**Principal component 3**	0.011	0.011	1.005	$3.15e^{-01}$	0.007	-0.006	0.019
**Numeric memory – Cardiometabolic principal components without imputed data:**							
**Principal component 1**	-0.015	0.009	-1.597	$1.10e^{-01}$	-0.011	-0.024	0.002
**Principal component 2**	-0.014	0.010	-1.409	$1.59e^{-01}$	-0.009	-0.022	0.003
**Principal component 3**	0.013	0.011	1.191	$2.34e^{-01}$	0.008	-0.005	0.021
**Numeric memory – Liver fat variables:**							
**Liver fat**	-0.008	0.024	-0.321	$7.48e^{-01}$	-0.002	-0.015	0.011
**Nonalcoholic fatty liver disease**	-0.015	0.035	-0.439	$6.61e^{-01}$	-0.003	-0.016	0.010
**Metabolic dysfunction-associated fatty liver disease**	-0.036	0.031	-1.140	$2.54e^{-01}$	-0.007	-0.020	0.005
**Metabolic dysfunction-associated steatotic liver disease**	-0.025	0.036	-0.693	$4.88e^{-01}$	-0.005	-0.017	0.008
**Numeric memory – White matter hyperintensities:**							
**White matter hyperintensities**	0.005	0.015	0.329	$7.42e^{-01}$	0.002	-0.011	0.015
**Fluid intelligence – Cardiometabolic risk factors:**							
**Body mass index**	-0.033	0.011	-2.901	$3.72e^{-03}$	-0.017	-0.028	-0.005
**Waist circumference**	-0.023	0.013	-1.815	$6.96e^{-02}$	-0.010	-0.022	0.001
**Systolic blood pressure**	-0.010	0.012	-0.823	$4.11e^{-01}$	-0.005	-0.016	0.007
**Diastolic blood pressure**	-0.011	0.011	-1.000	$3.17e^{-01}$	-0.006	-0.017	0.006
**Pulse pressure**	-0.002	0.012	-0.161	$8.72e^{-01}$	-0.001	-0.012	0.010
**C-reactive protein**	-0.015	0.019	-0.836	$4.03e^{-01}$	-0.005	-0.016	0.006
**Glycated hemoglobin**	-0.026	0.011	-2.301	$2.14e^{-02}$	-0.013	-0.025	-0.002

	β	SE	T	P	R	LCI	UCI
**High-density lipoprotein cholesterol**	-0.002	0.012	-0.148	$8.83e^{-01}$	-0.001	-0.012	0.010
**Low-density lipoprotein cholesterol**	0.010	0.011	0.901	$3.68e^{-01}$	0.005	-0.006	0.017
**Total cholesterol**	0.006	0.011	0.508	$6.11e^{-01}$	0.003	-0.008	0.014
**Triglycerides**	0.001	0.037	0.020	$9.84e^{-01}$	0.000	-0.011	0.011
**Fluid intelligence – Cardiometabolic principal components:**							
**Principal component 1**	-0.012	0.007	-1.638	$1.01e^{-01}$	-0.009	-0.021	0.002
**Principal component 2**	-0.009	0.008	-1.136	$2.56e^{-01}$	-0.007	-0.018	0.005
**Principal component 3**	0.004	0.009	0.428	$6.68e^{-01}$	0.002	-0.009	0.014
**Fluid intelligence – Cardiometabolic principal components without imputed data:**							
**Principal component 1**	-0.012	0.008	-1.585	$1.13e^{-01}$	-0.009	-0.021	0.002
**Principal component 2**	0.011	0.009	1.287	$1.98e^{-01}$	0.008	-0.004	0.019
**Principal component 3**	0.006	0.010	0.671	$5.03e^{-01}$	0.004	-0.007	0.015
**Fluid intelligence – Liver fat variables:**							
**Liver fat**	0.034	0.020	1.765	$7.76e^{-02}$	0.010	-0.001	0.021
**Nonalcoholic fatty liver disease**	0.029	0.030	0.956	$3.39e^{-01}$	0.006	-0.006	0.017
**Metabolic dysfunction-associated fatty liver disease**	0.008	0.027	0.315	$7.52e^{-01}$	0.002	-0.009	0.013
**Metabolic dysfunction-associated steatotic liver disease**	0.021	0.030	0.694	$4.88e^{-01}$	0.004	-0.007	0.015
**Fluid intelligence – White matter hyperintensities:**							
**White matter hyperintensities**	-0.025	0.012	-1.990	$4.66e^{-02}$	-0.011	-0.023	-0.000
**Trail making test part B – Cardiometabolic risk factors:**							
**Body mass index**	0.007	0.005	1.440	$1.50e^{-01}$	0.010	-0.004	0.024
**Waist circumference**	0.006	0.005	1.122	$2.62e^{-01}$	0.008	-0.006	0.022
**Systolic blood pressure**	-0.001	0.005	-0.242	$8.08e^{-01}$	-0.002	-0.016	0.012
**Diastolic blood pressure**	-0.002	0.005	-0.444	$6.57e^{-01}$	-0.003	-0.017	0.011
**Pulse pressure**	-0.000	0.005	-0.021	$9.83e^{-01}$	-0.000	-0.014	0.014
**C-reactive protein**	0.009	0.008	1.166	$2.44e^{-01}$	0.008	-0.006	0.022
**Glycated hemoglobin**	0.005	0.005	1.014	$3.11e^{-01}$	0.007	-0.007	0.021
**High-density lipoprotein cholesterol**	-0.001	0.005	-0.171	$8.64e^{-01}$	-0.001	-0.015	0.013
**Low-density lipoprotein cholesterol**	-0.000	0.005	-0.092	$9.26e^{-01}$	-0.001	-0.015	0.013
**Total cholesterol**	-0.001	0.005	-0.152	$8.80e^{-01}$	-0.001	-0.015	0.013
**Triglycerides**	0.003	0.016	0.204	$8.38e^{-01}$	0.001	-0.012	0.015
**Trail making test part B – Cardiometabolic principal components:**							
**Principal component 1**	0.002	0.003	0.481	$6.30e^{-01}$	0.003	-0.010	0.017
**Principal component 2**	0.001	0.003	0.317	$7.51e^{-01}$	0.002	-0.012	0.016
**Principal component 3**	-0.002	0.004	-0.424	$6.71e^{-01}$	-0.003	-0.017	0.011
**Trail making test part B – Cardiometabolic principal components without imputed data:**							

	β	SE	T	P	R	LCI	UCI
**Principal component 1**	-0.000	0.003	-0.026	$9.79e^{-01}$	-0.000	-0.014	0.014
**Principal component 2**	0.000	0.004	0.038	$9.70e^{-01}$	0.000	-0.014	0.014
**Principal component 3**	-0.001	0.004	-0.335	$7.38e^{-01}$	-0.002	-0.016	0.011
**Trail making test part B – Liver fat variables:**							
**Liver fat**	0.002	0.009	0.221	$8.25e^{-01}$	0.002	-0.012	0.015
**Nonalcoholic fatty liver disease**	0.000	0.013	0.029	$9.77e^{-01}$	0.000	-0.014	0.014
**Metabolic dysfunction-associated fatty liver disease**	0.015	0.011	1.342	$1.80e^{-01}$	0.009	-0.004	0.023
**Metabolic dysfunction-associated steatotic liver disease**	0.004	0.013	0.317	$7.51e^{-01}$	0.002	-0.012	0.016
**Trail making test part B – White matter hyperintensities:**							
**White matter hyperintensities**	0.002	0.005	0.296	$7.68e^{-01}$	0.002	-0.012	0.016
**Matrix test – Cardiometabolic risk factors:**							
**Body mass index**	-0.026	0.014	-1.897	$5.78e^{-02}$	-0.013	-0.027	0.000
**Waist circumference**	-0.018	0.016	-1.152	$2.49e^{-01}$	-0.008	-0.022	0.006
**Systolic blood pressure**	0.003	0.014	0.182	$8.56e^{-01}$	0.001	-0.012	0.015
**Diastolic blood pressure**	0.002	0.013	0.167	$8.67e^{-01}$	0.001	-0.013	0.015
**Pulse pressure**	0.003	0.014	0.207	$8.36e^{-01}$	0.001	-0.012	0.015
**C-reactive protein**	0.022	0.023	0.964	$3.35e^{-01}$	0.007	-0.007	0.020
**Glycated hemoglobin**	-0.015	0.013	-1.104	$2.70e^{-01}$	-0.008	-0.021	0.006
**High-density lipoprotein cholesterol**	0.013	0.015	0.851	$3.95e^{-01}$	0.006	-0.008	0.020
**Low-density lipoprotein cholesterol**	0.010	0.013	0.784	$4.33e^{-01}$	0.005	-0.008	0.019
**Total cholesterol**	0.016	0.014	1.163	$2.45e^{-01}$	0.008	-0.006	0.022
**Triglycerides**	0.037	0.045	0.824	$4.10e^{-01}$	0.006	-0.008	0.019
**Matrix test – Cardiometabolic principal components:**							
**Principal component 1**	-0.002	0.009	-0.229	$8.19e^{-01}$	-0.002	-0.015	0.012
**Principal component 2**	-0.011	0.010	-1.154	$2.49e^{-01}$	-0.008	-0.022	0.006
**Principal component 3**	0.007	0.011	0.664	$5.07e^{-01}$	0.005	-0.009	0.018
**Matrix test – Cardiometabolic principal components without imputed data:**							
**Principal component 1**	0.002	0.009	0.197	$8.44e^{-01}$	0.001	-0.012	0.015
**Principal component 2**	0.008	0.010	0.779	$4.36e^{-01}$	0.006	-0.008	0.019
**Principal component 3**	0.010	0.012	0.816	$4.14e^{-01}$	0.006	-0.008	0.020
**Matrix test – Liver fat variables:**							
**Liver fat**	0.014	0.024	0.576	$5.65e^{-01}$	0.004	-0.010	0.018
**Nonalcoholic fatty liver disease**	-0.022	0.036	-0.601	$5.48e^{-01}$	-0.004	-0.018	0.009
**Metabolic dysfunction-associated fatty liver disease**	-0.018	0.032	-0.569	$5.70e^{-01}$	-0.004	-0.018	0.010
**Metabolic dysfunction-associated steatotic liver disease**	-0.035	0.037	-0.948	$3.43e^{-01}$	-0.007	-0.020	0.007
**Matrix test – White matter hyperintensities:**							

	β	SE	T	P	R	LCI	UCI
**White matter hyperintensities**	-0.013	0.015	-0.872	$3.83e^{-01}$	-0.006	-0.020	0.008
**Symbol digit substitution – Cardiometabolic risk factors:**							
**Body mass index**	-0.031	0.013	-2.376	$1.75e^{-02}$	-0.017	-0.030	-0.003
**Waist circumference**	-0.020	0.015	-1.306	$1.91e^{-01}$	-0.009	-0.023	0.005
**Systolic blood pressure**	0.008	0.014	0.626	$5.31e^{-01}$	0.004	-0.009	0.018
**Diastolic blood pressure**	0.002	0.013	0.137	$8.91e^{-01}$	0.001	-0.013	0.015
**Pulse pressure**	0.013	0.014	0.966	$3.34e^{-01}$	0.007	-0.007	0.020
**C-reactive protein**	-0.001	0.022	-0.035	$9.72e^{-01}$	-0.000	-0.014	0.013
**Glycated hemoglobin**	-0.007	0.013	-0.576	$5.65e^{-01}$	-0.004	-0.018	0.010
**High-density lipoprotein cholesterol**	0.012	0.014	0.833	$4.05e^{-01}$	0.006	-0.008	0.020
**Low-density lipoprotein cholesterol**	0.021	0.013	1.673	$9.44e^{-02}$	0.012	-0.002	0.025
**Total cholesterol**	0.021	0.013	1.638	$1.01e^{-01}$	0.011	-0.002	0.025
**Triglycerides**	0.032	0.043	0.733	$4.64e^{-01}$	0.005	-0.009	0.019
**Symbol digit substitution – Cardiometabolic principal components:**							
**Principal component 1**	-0.002	0.009	-0.226	$8.21e^{-01}$	-0.002	-0.015	0.012
**Principal component 2**	-0.016	0.010	-1.642	$1.01e^{-01}$	-0.011	-0.025	0.002
**Principal component 3**	0.006	0.011	0.519	$6.04e^{-01}$	0.004	-0.010	0.017
**Symbol digit substitution – Cardiometabolic principal components without imputed data:**							
**Principal component 1**	-0.001	0.009	-0.059	$9.53e^{-01}$	-0.000	-0.014	0.013
**Principal component 2**	0.013	0.010	1.241	$2.15e^{-01}$	0.009	-0.005	0.023
**Principal component 3**	0.005	0.011	0.470	$6.38e^{-01}$	0.003	-0.010	0.017
**Symbol digit substitution – Liver fat variables:**							
**Liver fat**	-0.013	0.023	-0.575	$5.65e^{-01}$	-0.004	-0.018	0.010
**Nonalcoholic fatty liver disease**	0.028	0.035	0.801	$4.23e^{-01}$	0.006	-0.008	0.019
**Metabolic dysfunction-associated fatty liver disease**	-0.002	0.031	-0.052	$9.59e^{-01}$	-0.000	-0.014	0.013
**Metabolic dysfunction-associated steatotic liver disease**	0.028	0.035	0.786	$4.32e^{-01}$	0.005	-0.008	0.019
**Symbol digit substitution – White matter hyperintensities:**							
**White matter hyperintensities**	-0.015	0.015	-1.025	$3.05e^{-01}$	-0.007	-0.021	0.007
**Tower rearranging – Cardiometabolic risk factors:**							
**Body mass index**	-0.004	0.014	-0.267	$7.89e^{-01}$	-0.002	-0.016	0.012
**Waist circumference**	0.003	0.016	0.211	$8.33e^{-01}$	0.001	-0.012	0.015
**Systolic blood pressure**	-0.002	0.015	-0.133	$8.94e^{-01}$	-0.001	-0.015	0.013
**Diastolic blood pressure**	0.009	0.014	0.617	$5.37e^{-01}$	0.004	-0.009	0.018
**Pulse pressure**	-0.013	0.015	-0.829	$4.07e^{-01}$	-0.006	-0.020	0.008
**C-reactive protein**	0.012	0.024	0.525	$6.00e^{-01}$	0.004	-0.010	0.017
**Glycated hemoglobin**	0.007	0.014	0.485	$6.28e^{-01}$	0.003	-0.010	0.017

	β	SE	T	P	R	LCI	UCI
**High-density lipoprotein cholesterol**	-0.003	0.016	-0.167	$8.67e^{-01}$	-0.001	-0.015	0.013
**Low-density lipoprotein cholesterol**	0.020	0.014	1.404	$1.60e^{-01}$	0.010	-0.004	0.024
**Total cholesterol**	0.021	0.014	1.486	$1.37e^{-01}$	0.010	-0.003	0.024
**Triglycerides**	-0.001	0.047	-0.029	$9.77e^{-01}$	-0.000	-0.014	0.014
**Tower rearranging – Cardiometabolic principal components:**							
**Principal component 1**	0.002	0.009	0.226	$8.21e^{-01}$	0.002	-0.012	0.015
**Principal component 2**	-0.016	0.010	-1.535	$1.25e^{-01}$	-0.011	-0.025	0.003
**Principal component 3**	-0.009	0.012	-0.753	$4.51e^{-01}$	-0.005	-0.019	0.008
**Tower rearranging – Cardiometabolic principal components without imputed data:**							
**Principal component 1**	-0.003	0.010	-0.263	$7.92e^{-01}$	-0.002	-0.016	0.012
**Principal component 2**	0.016	0.011	1.418	$1.56e^{-01}$	0.010	-0.003	0.024
**Principal component 3**	-0.007	0.012	-0.575	$5.66e^{-01}$	-0.004	-0.018	0.010
**Tower rearranging – Liver fat variables:**							
**Liver fat**	0.032	0.026	1.267	$2.05e^{-01}$	0.009	-0.005	0.023
**Nonalcoholic fatty liver disease**	0.016	0.038	0.430	$6.67e^{-01}$	0.003	-0.011	0.017
**Metabolic dysfunction-associated fatty liver disease**	0.001	0.034	0.023	$9.82e^{-01}$	0.000	-0.014	0.014
**Metabolic dysfunction-associated steatotic liver disease**	0.013	0.039	0.332	$7.40e^{-01}$	0.002	-0.011	0.016
**Tower rearranging – White matter hyperintensities:**							
**White matter hyperintensities**	-0.002	0.016	-0.112	$9.11e^{-01}$	-0.001	-0.015	0.013
**Paired associate learning – Cardiometabolic risk factors:**							
**Body mass index**	-0.031	0.013	-2.408	$1.60e^{-02}$	-0.016	-0.028	-0.003
**Waist circumference**	-0.008	0.015	-0.569	$5.69e^{-01}$	-0.004	-0.016	0.009
**Systolic blood pressure**	-0.014	0.013	-1.033	$3.02e^{-01}$	-0.007	-0.020	0.006
**Diastolic blood pressure**	-0.021	0.013	-1.644	$1.00e^{-01}$	-0.011	-0.024	0.002
**Pulse pressure**	0.001	0.014	0.061	$9.51e^{-01}$	0.000	-0.012	0.013
**C-reactive protein**	-0.010	0.021	-0.456	$6.48e^{-01}$	-0.003	-0.016	0.010
**Glycated hemoglobin**	0.013	0.013	1.038	$2.99e^{-01}$	0.007	-0.006	0.020
**High-density lipoprotein cholesterol**	-0.002	0.014	-0.150	$8.81e^{-01}$	-0.001	-0.014	0.012
**Low-density lipoprotein cholesterol**	0.012	0.013	0.931	$3.52e^{-01}$	0.006	-0.007	0.019
**Total cholesterol**	0.009	0.013	0.710	$4.78e^{-01}$	0.005	-0.008	0.017
**Triglycerides**	0.067	0.043	1.558	$1.19e^{-01}$	0.010	-0.003	0.023
**Paired associate learning – Cardiometabolic principal components:**							
**Principal component 1**	-0.010	0.009	-1.178	$2.39e^{-01}$	-0.008	-0.020	0.005
**Principal component 2**	-0.004	0.009	-0.381	$7.03e^{-01}$	-0.002	-0.015	0.010
**Principal component 3**	-0.007	0.011	-0.662	$5.08e^{-01}$	-0.004	-0.017	0.008
**Paired associate learning – Cardiometabolic principal components without imputed data:**							

	β	SE	T	P	R	LCI	UCI
**Principal component 1**	-0.009	0.009	-1.000	$3.17e^{-01}$	-0.007	-0.020	0.006
**Principal component 2**	0.005	0.010	0.486	$6.27e^{-01}$	0.003	-0.009	0.016
**Principal component 3**	-0.009	0.011	-0.807	$4.19e^{-01}$	-0.005	-0.018	0.007
**Paired associate learning – Liver fat variables:**							
**Liver fat**	0.036	0.023	1.568	$1.17e^{-01}$	0.010	-0.003	0.023
**Nonalcoholic fatty liver disease**	0.054	0.034	1.583	$1.13e^{-01}$	0.010	-0.002	0.023
**Metabolic dysfunction-associated fatty liver disease**	0.013	0.031	0.433	$6.65e^{-01}$	0.003	-0.010	0.016
**Metabolic dysfunction-associated steatotic liver disease**	0.054	0.035	1.552	$1.21e^{-01}$	0.010	-0.003	0.023
**Paired associate learning – White matter hyperintensities:**							
**White matter hyperintensities**	-0.018	0.014	-1.242	$2.14e^{-01}$	-0.008	-0.021	0.005
**Pairs matching – Cardiometabolic risk factors:**							
**Body mass index**	0.003	0.007	0.460	$6.45e^{-01}$	0.003	-0.009	0.014
**Waist circumference**	0.007	0.008	0.779	$4.36e^{-01}$	0.004	-0.007	0.016
**Systolic blood pressure**	-0.008	0.008	-1.003	$3.16e^{-01}$	-0.006	-0.017	0.005
**Diastolic blood pressure**	-0.006	0.007	-0.758	$4.49e^{-01}$	-0.004	-0.016	0.007
**Pulse pressure**	-0.006	0.008	-0.728	$4.67e^{-01}$	-0.004	-0.015	0.007
**C-reactive protein**	-0.005	0.012	-0.398	$6.90e^{-01}$	-0.002	-0.013	0.009
**Glycated hemoglobin**	0.017	0.007	2.255	$2.42e^{-02}$	0.013	0.002	0.024
**High-density lipoprotein cholesterol**	0.006	0.008	0.704	$4.81e^{-01}$	0.004	-0.007	0.015
**Low-density lipoprotein cholesterol**	-0.003	0.007	-0.378	$7.05e^{-01}$	-0.002	-0.013	0.009
**Total cholesterol**	-0.001	0.007	-0.125	$9.01e^{-01}$	-0.001	-0.012	0.010
**Triglycerides**	0.020	0.025	0.807	$4.20e^{-01}$	0.005	-0.007	0.016
**Pairs matching – Cardiometabolic principal components:**							
**Principal component 1**	-0.004	0.005	-0.739	$4.60e^{-01}$	-0.004	-0.015	0.007
**Principal component 2**	0.004	0.005	0.649	$5.16e^{-01}$	0.004	-0.007	0.015
**Principal component 3**	-0.004	0.006	-0.588	$5.57e^{-01}$	-0.003	-0.015	0.008
**Pairs matching – Cardiometabolic principal components without imputed data:**							
**Principal component 1**	-0.004	0.005	-0.802	$4.22e^{-01}$	-0.005	-0.016	0.006
**Principal component 2**	-0.003	0.006	-0.439	$6.61e^{-01}$	-0.003	-0.014	0.009
**Principal component 3**	-0.003	0.006	-0.409	$6.82e^{-01}$	-0.002	-0.014	0.009
**Pairs matching – Liver fat variables:**							
**Liver fat**	0.006	0.013	0.489	$6.25e^{-01}$	0.003	-0.008	0.014
**Nonalcoholic fatty liver disease**	0.008	0.020	0.402	$6.88e^{-01}$	0.002	-0.009	0.014
**Metabolic dysfunction-associated fatty liver disease**	0.024	0.018	1.361	$1.74e^{-01}$	0.008	-0.003	0.019
**Metabolic dysfunction-associated steatotic liver disease**	0.008	0.020	0.383	$7.02e^{-01}$	0.002	-0.009	0.013
**Pairs matching – White matter hyperintensities:**							

	β	SE	T	P	R	LCI	UCI
**White matter hyperintensities**	0.004	0.008	0.424	$6.72e^{-01}$	0.002	-0.009	0.014

Notes: Results derived from multiple linear regression analyses adjusted for age, age^2, sex, interactions between age and sex, interactions between age^2 and sex, site, smoking status, alcohol consumption, intracranial volume, and education.

Abbreviations: β, Standardized beta; SE, Standard error; T, T-statistic; P, P-value; R, Partial correlation coefficient; LCI, Lower confidence interval; UCI, Upper confidence interval

Table S24: Mediation analyses on cardiometabolic factors, liver fat, and white matter hyperintensities

	Direct effect				Indirect effect				Total effect			
	β	SE	Z	P	β	SE	Z	P	β	SE	Z	P
**Cardiometabolic risk factors:**												
**BMI**	0.087	0.005	16.839	$0.00e^{+00}$	0.020	0.002	9.469	$0.00e^{+00}$	0.107	0.005	22.564	$0.00e^{+00}$
**WC**	0.101	0.006	16.755	$0.00e^{+00}$	0.023	0.002	9.356	$0.00e^{+00}$	0.124	0.006	22.531	$0.00e^{+00}$
**SBP**	0.095	0.005	18.452	$0.00e^{+00}$	0.010	0.001	12.818	$0.00e^{+00}$	0.105	0.005	20.589	$0.00e^{+00}$
**DBP**	0.103	0.005	20.785	$0.00e^{+00}$	0.012	0.001	12.464	$0.00e^{+00}$	0.115	0.005	23.697	$0.00e^{+00}$
**PP**	0.049	0.005	9.293	$0.00e^{+00}$	0.003	0.001	5.618	$1.94e^{-08}$	0.052	0.005	9.845	$0.00e^{+00}$
**CRP**	0.026	0.005	5.226	$1.73e^{-07}$	0.020	0.001	14.652	$0.00e^{+00}$	0.046	0.005	9.396	$0.00e^{+00}$
**HbA1c**	0.037	0.005	7.283	$3.26e^{-13}$	0.012	0.001	13.002	$0.00e^{+00}$	0.049	0.005	9.797	$0.00e^{+00}$
**HDL**	-0.022	0.005	-4.037	$5.42e^{-05}$	-0.024	0.002	-15.185	$0.00e^{+00}$	-0.046	0.005	-8.705	$0.00e^{+00}$
**LDL**	-0.002	0.005	-0.401	$6.88e^{-01}$	0.007	0.001	11.707	$0.00e^{+00}$	0.005	0.005	1.150	$2.50e^{-01}$
**CHOL**	-0.004	0.005	-0.863	$3.88e^{-01}$	0.004	0.001	7.296	$2.97e^{-13}$	-0.000	0.005	-0.051	$9.59e^{-01}$
**TG**	0.016	0.005	2.935	$3.33e^{-03}$	0.027	0.002	15.050	$0.00e^{+00}$	0.042	0.005	8.425	$0.00e^{+00}$
**Cardiometabolic principal components:**												
**PC1**	0.124	0.006	22.201	$0.00e^{+00}$	0.017	0.002	8.246	$2.22e^{-16}$	0.141	0.005	27.309	$0.00e^{+00}$
**PC2**	0.023	0.005	4.624	$3.77e^{-06}$	0.010	0.001	12.922	$0.00e^{+00}$	0.033	0.005	6.691	$2.21e^{-11}$
**PC3**	0.025	0.005	5.093	$3.53e^{-07}$	-0.020	0.001	-16.589	$0.00e^{+00}$	0.005	0.005	1.057	$2.90e^{-01}$
**Cardiometabolic principal components without imputed data:**												
**PC1**	0.117	0.006	20.403	$0.00e^{+00}$	0.016	0.002	7.738	$9.99e^{-15}$	0.133	0.005	24.715	$0.00e^{+00}$
**PC2**	-0.025	0.005	-4.906	$9.28e^{-07}$	-0.009	0.001	-11.759	$0.00e^{+00}$	-0.034	0.005	-6.625	$3.48e^{-11}$
**PC3**	0.023	0.005	4.494	$6.97e^{-06}$	-0.019	0.001	-15.181	$0.00e^{+00}$	0.004	0.005	0.833	$4.05e^{-01}$

Notes: Results derived from structural equation modeling mediation analyses adjusted for age, age^2, sex, site, smoking status, and alcohol consumption.

Abbreviations: β, Standardized beta; SE, Standard error; Z, Z-score; P, P-value; BMI, Body mass index; WC, Waist circumference; SBP, Systolic blood pressure; DBP, Diastolic blood pressure; PP, Pulse pressure; CRP, C-reactive protein; HbA1c, Glycated hemoglobin; HDL, High-density lipoprotein cholesterol; LDL, Low-density lipoprotein cholesterol; CHOL, Total cholesterol; TG, Triglycerides; PC, Principal component

Table S25: Mediation analyses on cardiometabolic factors, liver fat, and white matter hyperintensities, adjusted for cardiometabolic diagnoses

	Direct effect				Indirect effect				Total effect			
	β	SE	Z	P	β	SE	Z	P	β	SE	Z	P
**Cardiometabolic risk factors:**												
**BMI**	0.072	0.005	13.783	$0.00e^{+00}$	0.017	0.002	8.454	$0.00e^{+00}$	0.089	0.005	18.313	$0.00e^{+00}$
**WC**	0.084	0.006	13.788	$0.00e^{+00}$	0.020	0.002	8.312	$0.00e^{+00}$	0.104	0.006	18.404	$0.00e^{+00}$
**SBP**	0.085	0.005	16.343	$0.00e^{+00}$	0.008	0.001	10.874	$0.00e^{+00}$	0.092	0.005	17.918	$0.00e^{+00}$
**DBP**	0.095	0.005	18.989	$0.00e^{+00}$	0.010	0.001	10.464	$0.00e^{+00}$	0.105	0.005	21.256	$0.00e^{+00}$
**PP**	0.040	0.005	7.620	$2.53e^{-14}$	0.002	0.000	3.559	$3.72e^{-04}$	0.042	0.005	7.901	$2.66e^{-15}$
**CRP**	0.022	0.005	4.317	$1.58e^{-05}$	0.016	0.001	12.510	$0.00e^{+00}$	0.038	0.005	7.689	$1.49e^{-14}$
**HbA1c**	0.020	0.006	3.591	$3.30e^{-04}$	0.008	0.001	10.405	$0.00e^{+00}$	0.028	0.006	5.044	$4.55e^{-07}$
**HDL**	-0.011	0.005	-2.000	$4.55e^{-02}$	-0.019	0.001	-13.196	$0.00e^{+00}$	-0.030	0.005	-5.711	$1.12e^{-08}$
**LDL**	0.020	0.005	4.089	$4.32e^{-05}$	0.009	0.001	12.075	$0.00e^{+00}$	0.029	0.005	5.931	$3.01e^{-09}$
**CHOL**	0.018	0.005	3.667	$2.46e^{-04}$	0.006	0.001	10.428	$0.00e^{+00}$	0.024	0.005	4.899	$9.65e^{-07}$
**TG**	0.011	0.005	1.994	$4.62e^{-02}$	0.022	0.002	12.917	$0.00e^{+00}$	0.032	0.005	6.466	$1.01e^{-10}$
**Cardiometabolic principal components:**												
**PC1**	0.112	0.006	19.909	$0.00e^{+00}$	0.014	0.002	6.792	$1.11e^{-11}$	0.125	0.005	23.893	$0.00e^{+00}$
**PC2**	-0.002	0.005	-0.421	$6.74e^{-01}$	0.006	0.001	10.057	$0.00e^{+00}$	0.004	0.005	0.767	$4.43e^{-01}$
**PC3**	0.017	0.005	3.372	$7.47e^{-04}$	-0.017	0.001	-14.196	$0.00e^{+00}$	-0.001	0.005	-0.104	$9.17e^{-01}$
**Cardiometabolic principal components without imputed data:**												
**PC1**	0.107	0.006	18.461	$0.00e^{+00}$	0.013	0.002	6.645	$3.03e^{-11}$	0.120	0.005	21.980	$0.00e^{+00}$
**PC2**	-0.004	0.005	-0.691	$4.90e^{-01}$	-0.006	0.001	-9.431	$0.00e^{+00}$	-0.009	0.005	-1.738	$8.23e^{-02}$
**PC3**	0.016	0.005	2.992	$2.77e^{-03}$	-0.017	0.001	-13.233	$0.00e^{+00}$	-0.001	0.005	-0.232	$8.17e^{-01}$

Notes: Results derived from structural equation modeling mediation analyses adjusted for age, age^2, sex, site, smoking status, alcohol consumption, hypertension, diabetes, and dyslipidemia.

Abbreviations: β, Standardized beta; SE, Standard error; Z, Z-score; P, P-value; BMI, Body mass index; WC, Waist circumference; SBP, Systolic blood pressure; DBP, Diastolic blood pressure; PP, Pulse pressure; CRP, C-reactive protein; HbA1c, Glycated hemoglobin; HDL, High-density lipoprotein cholesterol; LDL, Low-density lipoprotein cholesterol; CHOL, Total cholesterol; TG, Triglycerides; PC, Principal component

Table S26: Sex-stratified mediation analyses on cardiometabolic factors, liver fat, and white matter hyperintensities

	Males												Females											
	Direct effect				Indirect effect				Total effect				Direct effect				Indirect effect				Total effect			
	β	SE	Z	P	β	SE	Z	P	β	SE	Z	P	β	SE	Z	P	β	SE	Z	P	β	SE	Z	P
**Cardiometabolic risk factors:**																								
**BMI**	0.117	0.008	15.581	$0.00e^{+00}$	0.018	0.003	6.421	$1.35e^{-10}$	0.136	0.007	19.493	$0.00e^{+00}$	0.067	0.007	9.268	$0.00e^{+00}$	0.018	0.003	6.151	$7.69e^{-10}$	0.085	0.007	12.965	$0.00e^{+00}$
**WC**	0.112	0.008	14.842	$0.00e^{+00}$	0.019	0.003	6.837	$8.06e^{-12}$	0.132	0.007	18.825	$0.00e^{+00}$	0.068	0.007	9.351	$0.00e^{+00}$	0.018	0.003	5.805	$6.42e^{-09}$	0.086	0.007	13.151	$0.00e^{+00}$
**SBP**	0.078	0.007	10.448	$0.00e^{+00}$	0.009	0.001	9.088	$0.00e^{+00}$	0.087	0.007	11.762	$0.00e^{+00}$	0.105	0.007	15.133	$0.00e^{+00}$	0.009	0.001	7.952	$1.78e^{-15}$	0.114	0.007	16.586	$0.00e^{+00}$
**DBP**	0.089	0.007	12.375	$0.00e^{+00}$	0.012	0.001	9.634	$0.00e^{+00}$	0.101	0.007	14.311	$0.00e^{+00}$	0.112	0.007	17.013	$0.00e^{+00}$	0.010	0.001	7.032	$2.04e^{-12}$	0.122	0.006	18.878	$0.00e^{+00}$
**PP**	0.034	0.008	4.486	$7.26e^{-06}$	0.001	0.001	1.705	$8.82e^{-02}$	0.036	0.008	4.640	$3.48e^{-06}$	0.057	0.007	7.888	$3.11e^{-15}$	0.003	0.001	4.977	$6.45e^{-07}$	0.060	0.007	8.358	$0.00e^{+00}$
**CRP**	0.033	0.008	4.413	$1.02e^{-05}$	0.017	0.002	10.494	$0.00e^{+00}$	0.050	0.007	6.745	$1.53e^{-11}$	0.022	0.007	3.217	$1.29e^{-03}$	0.020	0.002	9.061	$0.00e^{+00}$	0.042	0.007	6.440	$1.19e^{-10}$
**HbA1c**	0.037	0.007	5.036	$4.76e^{-07}$	0.013	0.001	9.706	$0.00e^{+00}$	0.050	0.007	6.879	$6.01e^{-12}$	0.033	0.007	4.901	$9.55e^{-07}$	0.011	0.001	8.394	$0.00e^{+00}$	0.044	0.007	6.520	$7.05e^{-11}$
**HDL**	-0.035	0.007	-4.760	$1.94e^{-06}$	-0.021	0.002	-10.841	$0.00e^{+00}$	-0.057	0.007	-7.882	$3.33e^{-15}$	-0.013	0.007	-1.889	$5.89e^{-02}$	-0.020	0.002	-9.575	$0.00e^{+00}$	-0.033	0.006	-5.101	$3.38e^{-07}$
**LDL**	-0.018	0.007	-2.558	$1.05e^{-02}$	0.005	0.001	5.647	$1.63e^{-08}$	-0.013	0.007	-1.866	$6.20e^{-02}$	0.004	0.007	0.553	$5.80e^{-01}$	0.008	0.001	8.648	$0.00e^{+00}$	0.012	0.007	1.764	$7.78e^{-02}$
**CHOL**	-0.021	0.007	-3.050	$2.28e^{-03}$	0.003	0.001	3.243	$1.18e^{-03}$	-0.019	0.007	-2.661	$7.78e^{-03}$	0.001	0.007	0.157	$8.76e^{-01}$	0.004	0.001	5.308	$1.11e^{-07}$	0.005	0.007	0.686	$4.93e^{-01}$
**TG**	0.009	0.007	1.255	$2.09e^{-01}$	0.026	0.002	11.595	$0.00e^{+00}$	0.035	0.007	4.980	$6.37e^{-07}$	0.015	0.007	2.077	$3.78e^{-02}$	0.025	0.003	9.402	$0.00e^{+00}$	0.039	0.007	5.993	$2.06e^{-09}$
**Cardiometabolic principal components:**																								
**PC1**	0.113	0.008	14.984	$0.00e^{+00}$	0.019	0.002	7.555	$4.20e^{-14}$	0.131	0.007	18.638	$0.00e^{+00}$	0.116	0.007	15.888	$0.00e^{+00}$	0.011	0.003	3.949	$7.86e^{-05}$	0.128	0.007	18.934	$0.00e^{+00}$
**PC2**	0.047	0.007	6.689	$2.24e^{-11}$	0.010	0.001	9.362	$0.00e^{+00}$	0.058	0.007	8.142	$4.44e^{-16}$	0.010	0.007	1.449	$1.47e^{-01}$	0.010	0.001	9.121	$0.00e^{+00}$	0.020	0.007	2.985	$2.84e^{-03}$
**PC3**	0.006	0.007	0.839	$4.02e^{-01}$	-0.019	0.002	-11.503	$0.00e^{+00}$	-0.013	0.007	-1.745	$8.09e^{-02}$	0.042	0.007	6.197	$5.77e^{-10}$	-0.020	0.002	-11.435	$0.00e^{+00}$	0.022	0.007	3.370	$7.51e^{-04}$
**Cardiometabolic principal components without imputed data:**																								
**PC1**	0.104	0.008	13.371	$0.00e^{+00}$	0.018	0.002	7.279	$3.36e^{-13}$	0.123	0.007	16.607	$0.00e^{+00}$	0.111	0.008	14.336	$0.00e^{+00}$	0.010	0.003	3.450	$5.61e^{-04}$	0.121	0.007	17.009	$0.00e^{+00}$
**PC2**	-0.052	0.008	-6.939	$3.94e^{-12}$	-0.009	0.001	-8.596	$0.00e^{+00}$	-0.061	0.007	-8.175	$2.22e^{-16}$	-0.010	0.007	-1.451	$1.47e^{-01}$	-0.008	0.001	-8.008	$1.11e^{-15}$	-0.019	0.007	-2.662	$7.77e^{-03}$
**PC3**	-0.000	0.008	-0.004	$9.97e^{-01}$	-0.018	0.002	-10.689	$0.00e^{+00}$	-0.019	0.008	-2.456	$1.41e^{-02}$	0.043	0.007	6.167	$6.95e^{-10}$	-0.018	0.002	-10.287	$0.00e^{+00}$	0.025	0.007	3.656	$2.56e^{-04}$

Notes: Results derived from structural equation modeling mediation analyses adjusted for age, age^2, site, smoking status, and alcohol consumption.

Abbreviations: β, Standardized beta; SE, Standard error; Z, Z-score; P, P-value; BMI, Body mass index; WC, Waist circumference; SBP, Systolic blood pressure; DBP, Diastolic blood pressure; PP, Pulse pressure; CRP, C-reactive protein; HbA1c, Glycated hemoglobin; HDL, High-density lipoprotein cholesterol; LDL, Low-density lipoprotein cholesterol; CHOL, Total cholesterol; TG, Triglycerides; PC, Principal component

Table S27: Sex-stratified mediation analyses on cardiometabolic factors, liver fat, and white matter hyperintensities, adjusted for cardiometabolic diagnoses

	Males												Females											
	Direct effect				Indirect effect				Total effect				Direct effect				Indirect effect				Total effect			
	β	SE	Z	P	β	SE	Z	P	β	SE	Z	P	β	SE	Z	P	β	SE	Z	P	β	SE	Z	P
**Cardiometabolic risk factors:**																								
**BMI**	0.101	0.008	13.205	$0.00e^{+00}$	0.016	0.003	5.903	$3.57e^{-09}$	0.118	0.007	16.331	$0.00e^{+00}$	0.052	0.007	7.188	$6.58e^{-13}$	0.015	0.003	5.183	$2.18e^{-07}$	0.067	0.007	9.982	$0.00e^{+00}$
**WC**	0.097	0.008	12.679	$0.00e^{+00}$	0.017	0.003	6.210	$5.29e^{-10}$	0.114	0.007	15.880	$0.00e^{+00}$	0.053	0.007	7.278	$3.40e^{-13}$	0.015	0.003	4.905	$9.33e^{-07}$	0.068	0.007	10.163	$0.00e^{+00}$
**SBP**	0.068	0.007	9.082	$0.00e^{+00}$	0.007	0.001	7.849	$4.22e^{-15}$	0.075	0.007	10.062	$0.00e^{+00}$	0.093	0.007	13.295	$0.00e^{+00}$	0.006	0.001	6.441	$1.19e^{-10}$	0.099	0.007	14.279	$0.00e^{+00}$
**DBP**	0.081	0.007	11.261	$0.00e^{+00}$	0.010	0.001	8.367	$0.00e^{+00}$	0.091	0.007	12.810	$0.00e^{+00}$	0.103	0.007	15.475	$0.00e^{+00}$	0.007	0.001	5.514	$3.51e^{-08}$	0.110	0.007	16.812	$0.00e^{+00}$
**PP**	0.027	0.008	3.488	$4.86e^{-04}$	0.000	0.001	0.335	$7.37e^{-01}$	0.027	0.008	3.502	$4.61e^{-04}$	0.046	0.007	6.397	$1.58e^{-10}$	0.002	0.001	3.138	$1.70e^{-03}$	0.048	0.007	6.627	$3.43e^{-11}$
**CRP**	0.030	0.008	4.006	$6.17e^{-05}$	0.014	0.001	9.159	$0.00e^{+00}$	0.044	0.007	5.939	$2.87e^{-09}$	0.016	0.007	2.298	$2.16e^{-02}$	0.015	0.002	7.407	$1.29e^{-13}$	0.031	0.007	4.762	$1.91e^{-06}$
**HbA1c**	0.018	0.008	2.104	$3.54e^{-02}$	0.008	0.001	7.512	$5.82e^{-14}$	0.026	0.008	3.112	$1.86e^{-03}$	0.018	0.007	2.399	$1.64e^{-02}$	0.007	0.001	6.659	$2.75e^{-11}$	0.024	0.007	3.316	$9.12e^{-04}$
**HDL**	-0.024	0.007	-3.203	$1.36e^{-03}$	-0.018	0.002	-9.569	$0.00e^{+00}$	-0.042	0.007	-5.726	$1.03e^{-08}$	-0.004	0.007	-0.533	$5.94e^{-01}$	-0.016	0.002	-8.009	$1.11e^{-15}$	-0.019	0.007	-2.949	$3.19e^{-03}$
**LDL**	0.011	0.008	1.400	$1.62e^{-01}$	0.008	0.001	8.088	$6.66e^{-16}$	0.019	0.008	2.482	$1.31e^{-02}$	0.019	0.007	2.734	$6.26e^{-03}$	0.008	0.001	7.597	$3.04e^{-14}$	0.026	0.007	3.913	$9.13e^{-05}$
**CHOL**	0.007	0.007	0.902	$3.67e^{-01}$	0.006	0.001	6.961	$3.39e^{-12}$	0.013	0.007	1.723	$8.48e^{-02}$	0.017	0.007	2.384	$1.71e^{-02}$	0.005	0.001	6.589	$4.43e^{-11}$	0.021	0.007	3.059	$2.22e^{-03}$
**TG**	0.006	0.007	0.832	$4.06e^{-01}$	0.022	0.002	10.022	$0.00e^{+00}$	0.028	0.007	3.937	$8.27e^{-05}$	0.006	0.007	0.878	$3.80e^{-01}$	0.019	0.002	7.828	$4.88e^{-15}$	0.026	0.007	3.889	$1.01e^{-04}$
**Cardiometabolic principal components:**																								
**PC1**	0.103	0.008	13.634	$0.00e^{+00}$	0.015	0.002	6.463	$1.03e^{-10}$	0.118	0.007	16.585	$0.00e^{+00}$	0.103	0.007	13.877	$0.00e^{+00}$	0.008	0.003	2.946	$3.22e^{-03}$	0.111	0.007	16.078	$0.00e^{+00}$
**PC2**	0.020	0.008	2.625	$8.67e^{-03}$	0.006	0.001	6.973	$3.11e^{-12}$	0.026	0.008	3.429	$6.05e^{-04}$	-0.009	0.007	-1.355	$1.75e^{-01}$	0.007	0.001	7.345	$2.06e^{-13}$	-0.003	0.007	-0.419	$6.75e^{-01}$
**PC3**	-0.004	0.007	-0.572	$5.67e^{-01}$	-0.017	0.002	-10.012	$0.00e^{+00}$	-0.021	0.007	-2.837	$4.55e^{-03}$	0.035	0.007	5.116	$3.12e^{-07}$	-0.016	0.002	-9.369	$0.00e^{+00}$	0.019	0.007	2.795	$5.20e^{-03}$
**Cardiometabolic principal components without imputed data:**																								
**PC1**	0.096	0.008	12.219	$0.00e^{+00}$	0.015	0.002	6.362	$1.99e^{-10}$	0.111	0.007	14.933	$0.00e^{+00}$	0.099	0.008	12.715	$0.00e^{+00}$	0.008	0.003	2.763	$5.73e^{-03}$	0.107	0.007	14.782	$0.00e^{+00}$
**PC2**	-0.028	0.008	-3.458	$5.43e^{-04}$	-0.006	0.001	-6.563	$5.27e^{-11}$	-0.034	0.008	-4.182	$2.89e^{-05}$	0.005	0.007	0.751	$4.52e^{-01}$	-0.006	0.001	-6.660	$2.73e^{-11}$	-0.000	0.007	-0.027	$9.78e^{-01}$
**PC3**	-0.009	0.008	-1.223	$2.21e^{-01}$	-0.016	0.002	-9.367	$0.00e^{+00}$	-0.026	0.008	-3.410	$6.50e^{-04}$	0.036	0.007	5.172	$2.31e^{-07}$	-0.016	0.002	-8.688	$0.00e^{+00}$	0.021	0.007	3.042	$2.35e^{-03}$

Notes: Results derived from structural equation modeling mediation analyses adjusted for age, age^2, site, smoking status, alcohol consumption, hypertension, diabetes, and dyslipidemia.

Abbreviations: β, Standardized beta; SE, Standard error; Z, Z-score; P, P-value; BMI, Body mass index; WC, Waist circumference; SBP, Systolic blood pressure; DBP, Diastolic blood pressure; PP, Pulse pressure; CRP, C-reactive protein; HbA1c, Glycated hemoglobin; HDL, High-density lipoprotein cholesterol; LDL, Low-density lipoprotein cholesterol; CHOL, Total cholesterol; TG, Triglycerides; PC, Principal component

Table S28: Mediation analyses on liver fat variables, white matter hyperintensities, and general cognitive performance

	Direct effect				Indirect effect				Total effect			
	β	SE	Z	P	β	SE	Z	P	β	SE	Z	P
**LF**	-0.020	0.006	-3.341	$8.35e^{-04}$	-0.006	0.001	-8.460	$0.00e^{+00}$	-0.026	0.006	-4.418	$9.95e^{-06}$
**NAFLD**	-0.015	0.006	-2.596	$9.42e^{-03}$	-0.004	0.001	-7.169	$7.56e^{-13}$	-0.019	0.006	-3.276	$1.05e^{-03}$
**MAFLD**	-0.021	0.006	-3.568	$3.60e^{-04}$	-0.005	0.001	-8.168	$2.22e^{-16}$	-0.026	0.006	-4.465	$8.00e^{-06}$
**MASLD**	-0.015	0.006	-2.567	$1.03e^{-02}$	-0.004	0.001	-7.175	$7.21e^{-13}$	-0.019	0.006	-3.248	$1.16e^{-03}$

Notes: Results derived from structural equation modeling mediation analyses adjusted for age, age^2, sex, site, smoking status, alcohol consumption, intracranial volume, and education.

Abbreviations: β, Standardized beta; SE, Standard error; Z, Z-score; P, P-value; LF, Liver fat; NAFLD, Nonalcoholic fatty liver disease; MAFLD, Metabolic dysfunction-associated fatty liver disease; MASLD, Metabolic dysfunction-associated steatotic liver disease

Table S29: Mediation analyses on liver fat variables, white matter hyperintensities, and cognitive tests

	Direct effect				Indirect effect				Total effect			
	β	SE	Z	P	β	SE	Z	P	β	SE	Z	P
**Liver fat:**												
**Numeric memory**	-0.039	0.006	-6.032	$1.62e^{-09}$	-0.003	0.001	-4.855	$1.20e^{-06}$	-0.042	0.006	-6.594	$4.28e^{-11}$
**Fluid intelligence**	-0.009	0.006	-1.545	$1.22e^{-01}$	-0.004	0.001	-6.712	$1.92e^{-11}$	-0.013	0.005	-2.340	$1.93e^{-02}$
**Trail making test B**	-0.004	0.007	-0.624	$5.32e^{-01}$	0.005	0.001	6.488	$8.70e^{-11}$	0.001	0.006	0.143	$8.86e^{-01}$
**Matrix test**	-0.019	0.007	-2.836	$4.57e^{-03}$	-0.005	0.001	-6.255	$3.97e^{-10}$	-0.024	0.007	-3.570	$3.57e^{-04}$
**Symbol digit substitution**	-0.021	0.007	-3.219	$1.29e^{-03}$	-0.006	0.001	-7.212	$5.53e^{-13}$	-0.027	0.007	-4.087	$4.36e^{-05}$
**Tower rearranging**	0.029	0.007	4.123	$3.74e^{-05}$	-0.004	0.001	-4.859	$1.18e^{-06}$	0.025	0.007	3.591	$3.30e^{-04}$
**Paired associate learning**	-0.036	0.006	-5.570	$2.55e^{-08}$	-0.004	0.001	-5.871	$4.32e^{-09}$	-0.040	0.006	-6.259	$3.86e^{-10}$
**Pairs matching**	-0.021	0.006	-3.667	$2.45e^{-04}$	0.002	0.001	3.504	$4.58e^{-04}$	-0.019	0.006	-3.310	$9.32e^{-04}$
**Nonalcoholic fatty liver disease:**												
**Numeric memory**	-0.031	0.007	-4.683	$2.83e^{-06}$	-0.002	0.000	-4.774	$1.80e^{-06}$	-0.033	0.007	-5.043	$4.59e^{-07}$
**Fluid intelligence**	-0.001	0.006	-0.178	$8.59e^{-01}$	-0.003	0.000	-6.227	$4.76e^{-10}$	-0.004	0.005	-0.681	$4.96e^{-01}$
**Trail making test B**	-0.004	0.007	-0.617	$5.38e^{-01}$	0.003	0.001	5.733	$9.86e^{-09}$	-0.001	0.007	-0.143	$8.87e^{-01}$
**Matrix test**	-0.020	0.007	-2.964	$3.03e^{-03}$	-0.003	0.001	-5.570	$2.55e^{-08}$	-0.023	0.007	-3.419	$6.28e^{-04}$
**Symbol digit substitution**	-0.013	0.007	-2.010	$4.44e^{-02}$	-0.004	0.001	-6.284	$3.29e^{-10}$	-0.017	0.007	-2.563	$1.04e^{-02}$
**Tower rearranging**	0.018	0.007	2.584	$9.75e^{-03}$	-0.002	0.001	-4.406	$1.05e^{-05}$	0.016	0.007	2.257	$2.40e^{-02}$
**Paired associate learning**	-0.027	0.006	-4.194	$2.74e^{-05}$	-0.003	0.001	-5.557	$2.75e^{-08}$	-0.030	0.006	-4.628	$3.69e^{-06}$
**Pairs matching**	-0.022	0.006	-3.749	$1.78e^{-04}$	0.001	0.000	3.253	$1.14e^{-03}$	-0.020	0.006	-3.526	$4.22e^{-04}$
**Metabolic dysfunction-associated fatty liver disease:**												
**Numeric memory**	-0.034	0.006	-5.375	$7.66e^{-08}$	-0.003	0.001	-4.940	$7.82e^{-07}$	-0.037	0.006	-5.859	$4.66e^{-09}$
**Fluid intelligence**	-0.005	0.005	-0.829	$4.07e^{-01}$	-0.004	0.001	-6.480	$9.15e^{-11}$	-0.008	0.005	-1.477	$1.40e^{-01}$
**Trail making test B**	0.001	0.007	0.089	$9.29e^{-01}$	0.004	0.001	6.176	$6.57e^{-10}$	0.005	0.006	0.715	$4.75e^{-01}$
**Matrix test**	-0.016	0.007	-2.363	$1.81e^{-02}$	-0.004	0.001	-6.096	$1.09e^{-09}$	-0.020	0.007	-2.966	$3.02e^{-03}$
**Symbol digit substitution**	-0.025	0.006	-3.941	$8.11e^{-05}$	-0.005	0.001	-6.847	$7.55e^{-12}$	-0.030	0.006	-4.662	$3.13e^{-06}$
**Tower rearranging**	0.025	0.007	3.614	$3.02e^{-04}$	-0.003	0.001	-4.689	$2.75e^{-06}$	0.022	0.007	3.175	$1.50e^{-03}$
**Paired associate learning**	-0.038	0.006	-5.855	$4.78e^{-09}$	-0.004	0.001	-5.879	$4.12e^{-09}$	-0.041	0.006	-6.431	$1.27e^{-10}$
**Pairs matching**	-0.023	0.006	-4.023	$5.74e^{-05}$	0.002	0.001	3.425	$6.14e^{-04}$	-0.021	0.006	-3.718	$2.01e^{-04}$
**Metabolic dysfunction-associated steatotic liver disease:**												
**Numeric memory**	-0.030	0.007	-4.592	$4.39e^{-06}$	-0.002	0.000	-4.802	$1.57e^{-06}$	-0.033	0.007	-4.955	$7.24e^{-07}$
**Fluid intelligence**	-0.000	0.005	-0.037	$9.71e^{-01}$	-0.003	0.000	-6.065	$1.32e^{-09}$	-0.003	0.005	-0.550	$5.83e^{-01}$
**Trail making test B**	-0.002	0.007	-0.358	$7.20e^{-01}$	0.003	0.001	5.731	$9.97e^{-09}$	0.001	0.007	0.119	$9.05e^{-01}$
**Matrix test**	-0.019	0.007	-2.830	$4.66e^{-03}$	-0.003	0.001	-5.622	$1.89e^{-08}$	-0.022	0.007	-3.282	$1.03e^{-03}$
**Symbol digit substitution**	-0.013	0.007	-1.991	$4.64e^{-02}$	-0.004	0.001	-6.362	$2.00e^{-10}$	-0.017	0.007	-2.538	$1.12e^{-02}$
**Tower rearranging**	0.019	0.007	2.672	$7.54e^{-03}$	-0.002	0.001	-4.374	$1.22e^{-05}$	0.017	0.007	2.348	$1.89e^{-02}$

	Direct effect				Indirect effect				Total effect			
	β	SE	Z	P	β	SE	Z	P	β	SE	Z	P
**Paired associate learning**	-0.029	0.007	-4.434	$9.23e^{-06}$	-0.003	0.000	-5.671	$1.42e^{-08}$	-0.032	0.007	-4.870	$1.12e^{-06}$
**Pairs matching**	-0.024	0.006	-4.079	$4.51e^{-05}$	0.001	0.000	3.284	$1.02e^{-03}$	-0.022	0.006	-3.853	$1.17e^{-04}$

Notes: Results derived from structural equation modeling mediation analyses adjusted for age, age^2, sex, site, smoking status, alcohol consumption, intracranial volume, education, hypertension, diabetes, and dyslipidemia.

Abbreviations: β, Standardized beta; SE, Standard error; Z, Z-score; P, P-value

Table S30: Mediation analyses on liver fat variables, white matter hyperintensities, and general cognitive performance, without imputed data

	Direct effect				Indirect effect				Total effect			
	β	SE	Z	P	β	SE	Z	P	β	SE	Z	P
**LF**	0.016	0.007	2.398	$1.65e^{-02}$	0.006	0.001	7.745	$9.55e^{-15}$	0.022	0.007	3.368	$7.56e^{-04}$
**NAFLD**	0.013	0.007	1.904	$5.69e^{-02}$	0.004	0.001	6.492	$8.48e^{-11}$	0.017	0.007	2.508	$1.21e^{-02}$
**MAFLD**	0.014	0.007	2.089	$3.67e^{-02}$	0.005	0.001	7.333	$2.24e^{-13}$	0.019	0.007	2.886	$3.90e^{-03}$
**MASLD**	0.011	0.007	1.674	$9.41e^{-02}$	0.004	0.001	6.623	$3.51e^{-11}$	0.015	0.007	2.277	$2.28e^{-02}$

Notes: Results derived from structural equation modeling mediation analyses adjusted for age, age^2, sex, site, smoking status, alcohol consumption, intracranial volume, and education.

Abbreviations: β, Standardized beta; SE, Standard error; Z, Z-score; P, P-value; LF, Liver fat; NAFLD, Nonalcoholic fatty liver disease; MAFLD, Metabolic dysfunction-associated fatty liver disease; MASLD, Metabolic dysfunction-associated steatotic liver disease

Table S31: Mediation analyses on liver fat variables, white matter hyperintensities, and general cognitive performance, adjusted for cardiometabolic diagnoses

	Direct effect				Indirect effect				Total effect			
	β	SE	Z	P	β	SE	Z	P	β	SE	Z	P
**LF**	-0.015	0.006	-2.560	$1.05e^{-02}$	-0.005	0.001	-7.686	$1.51e^{-14}$	-0.020	0.006	-3.409	$6.53e^{-04}$
**NAFLD**	-0.012	0.006	-1.952	$5.10e^{-02}$	-0.003	0.000	-6.140	$8.24e^{-10}$	-0.015	0.006	-2.458	$1.40e^{-02}$
**MAFLD**	-0.016	0.006	-2.689	$7.16e^{-03}$	-0.004	0.001	-7.161	$8.02e^{-13}$	-0.020	0.006	-3.359	$7.83e^{-04}$
**MASLD**	-0.011	0.006	-1.891	$5.87e^{-02}$	-0.003	0.000	-6.116	$9.61e^{-10}$	-0.014	0.006	-2.394	$1.67e^{-02}$

Notes: Results derived from structural equation modeling mediation analyses adjusted for age, age^2, sex, site, smoking status, alcohol consumption, intracranial volume, education, hypertension, diabetes, and dyslipidemia.

Abbreviations: β, Standardized beta; SE, Standard error; Z, Z-score; P, P-value

Supplementary figures

Figure S1: Flow chart of the study

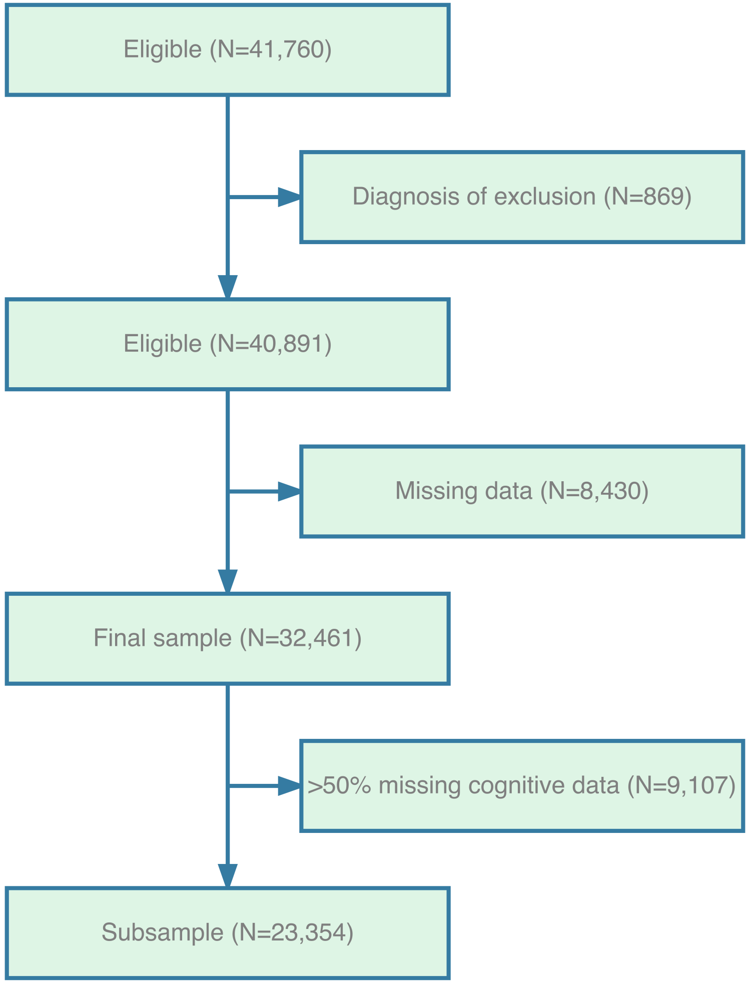

Figure S2: Correlations of the cardiometabolic variables

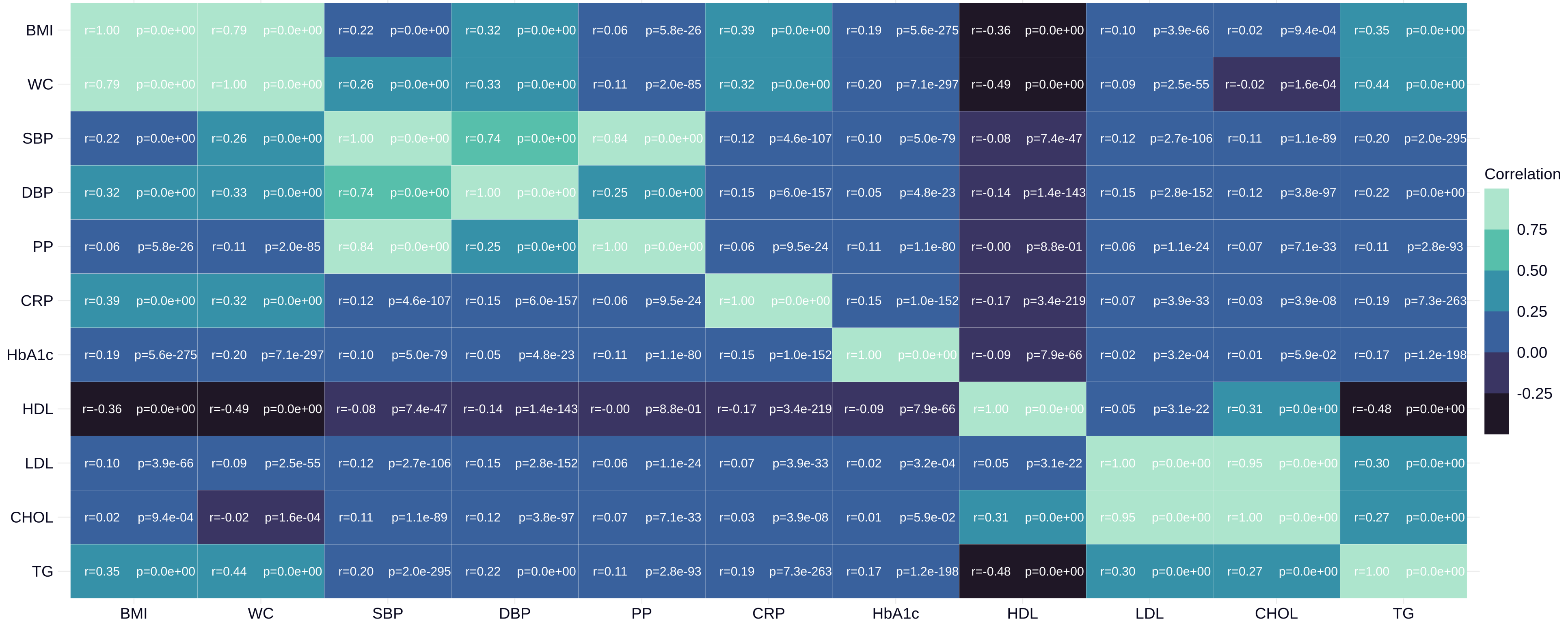

Notes: Pearson correlation matrix of the 11 included cardiometabolic variables.

Abbreviations: r, Partial correlation coefficient; p, P-value; BMI, Body mass index; WC, Waist circumference; SBP, Systolic blood pressure; DBP, Diastolic blood pressure; PP, Pulse pressure; CRP, C-reactive protein; HbA1c, Glycated hemoglobin; HDL, High-density lipoprotein cholesterol; LDL, Low-density lipoprotein cholesterol; CHOL, Total cholesterol; TG, Triglycerides

Figure S3: Histograms of the cardiometabolic and imaging-related variables

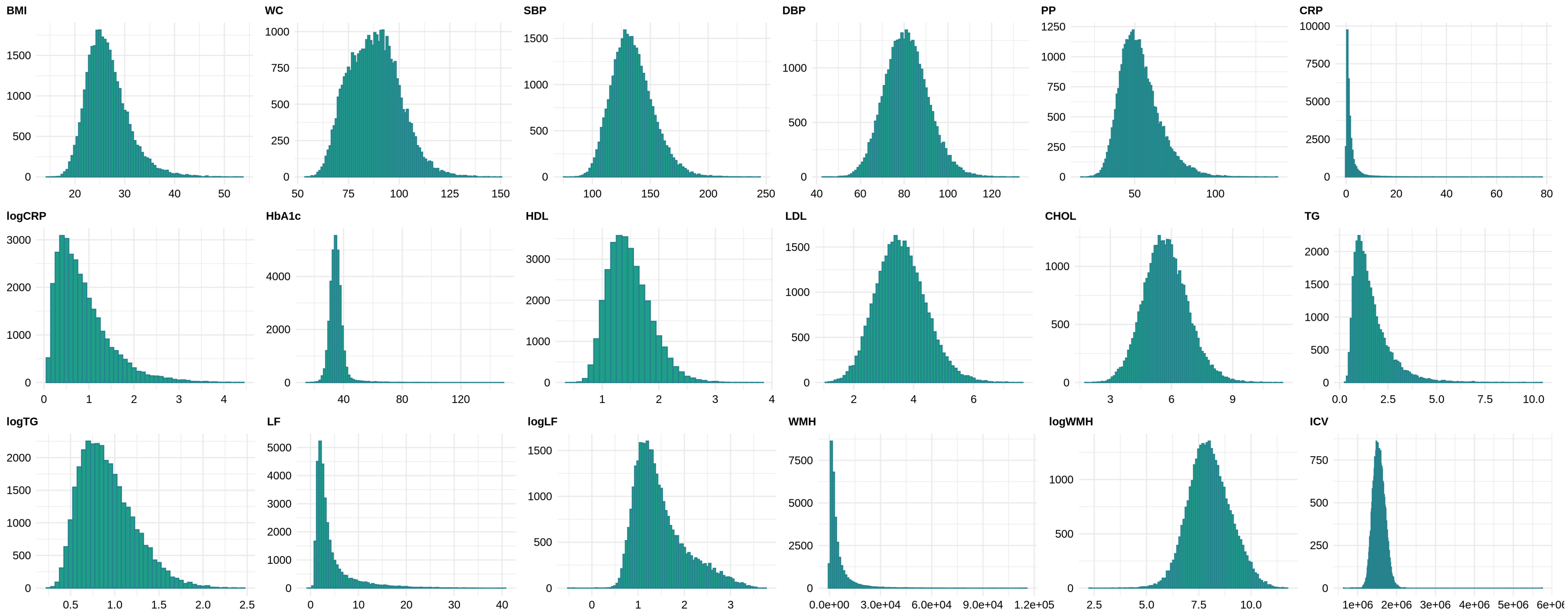

Notes: Histograms of the cardiometabolic and imaging-related variables.

Abbreviations: BMI, Body mass index; WC, Waist circumference; SBP, Systolic blood pressure; DBP, Diastolic blood pressure; PP, Pulse pressure; CRP, C-reactive protein; HbA1c, Glycated hemoglobin; HDL, High-density lipoprotein cholesterol; LDL, Low-density lipoprotein cholesterol; CHOL, Total cholesterol; TG, Triglycerides; LF, Liver fat; WMH, White matter hyperintensities; ICV, Intracranial volume

Figure S4: Quantile-quantile plots of the cardiometabolic and imaging-related variables

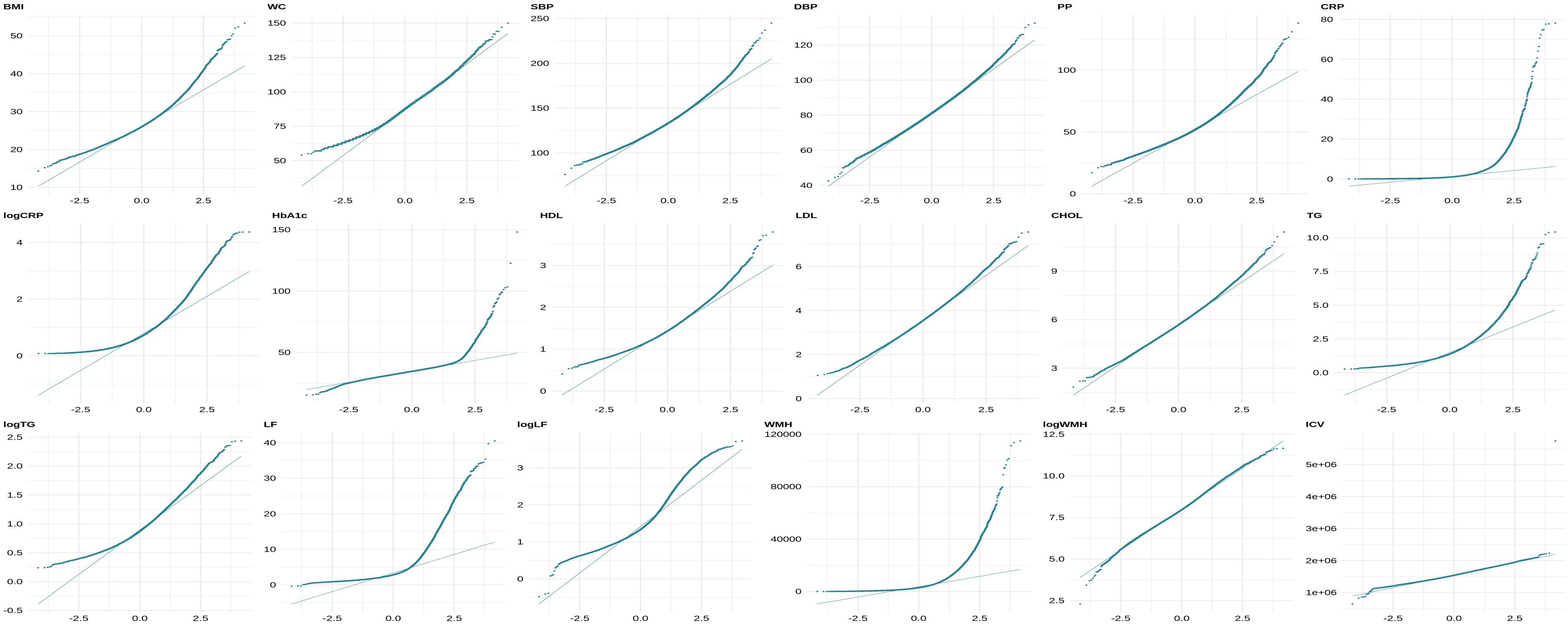

Notes: Quantile-quantile-plots of the cardiometabolic and imaging-related variables.
Abbreviations: BMI, Body mass index; WC, Waist circumference; SBP, Systolic blood pressure; DBP, Diastolic blood pressure; PP, Pulse pressure; CRP, C-reactive protein; HbA1c, Glycated hemoglobin; HDL, High-density lipoprotein cholesterol; LDL, Low-density lipoprotein cholesterol; CHOL, Total cholesterol; TG, Triglycerides; LF, Liver fat; WMH, White matter hyperintensities; ICV, Intracranial volume

Figure S5: Cardiometabolic principal component analysis

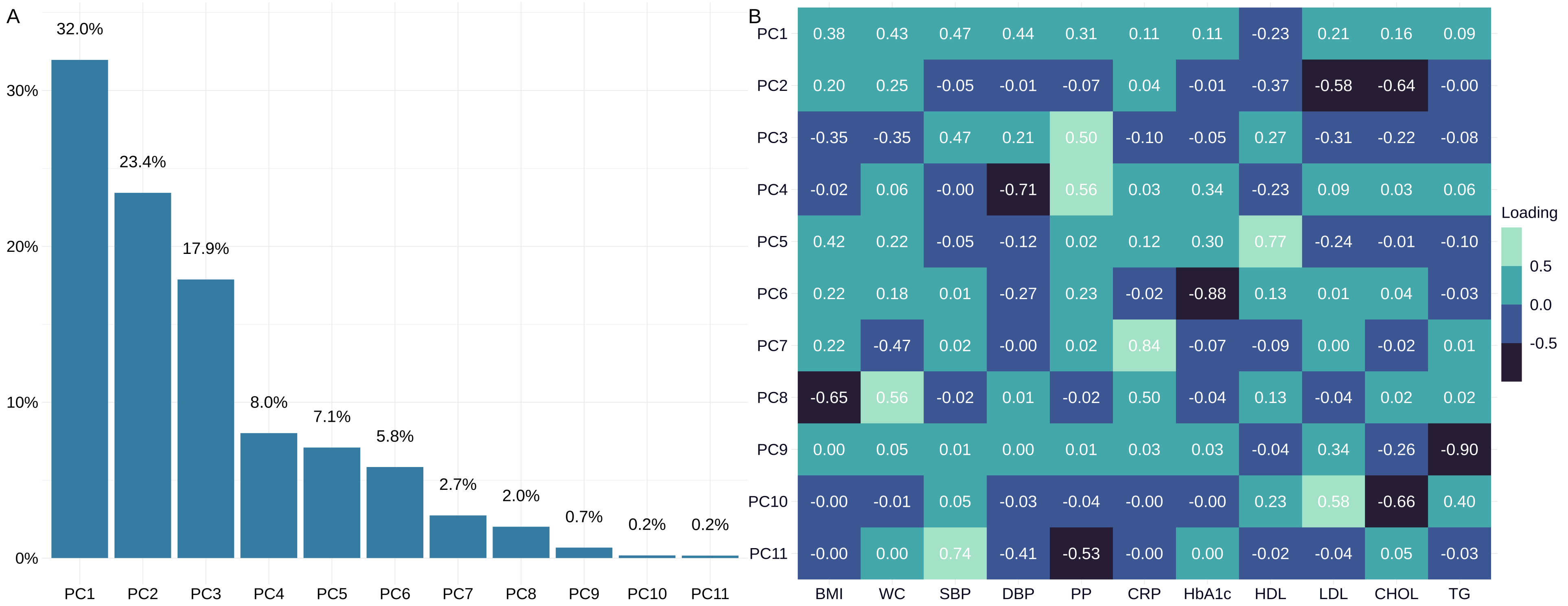

Notes: A) The explained variance of the 11 principal components from cardiometabolic principal component analysis and B) their loadings from the 11 included cardiometabolic variables.
Abbreviations: PC, Principal component; BMI, Body mass index; WC, Waist circumference; SBP, Systolic blood pressure; DBP, Diastolic blood pressure; PP, Pulse pressure; CRP, C-reactive protein; HbA1c, Glycated hemoglobin; HDL, High-density lipoprotein cholesterol; LDL, Low-density lipoprotein cholesterol; CHOL, Total cholesterol; TG, Triglycerides

Figure S6: Cardiometabolic principal component analysis without imputed data

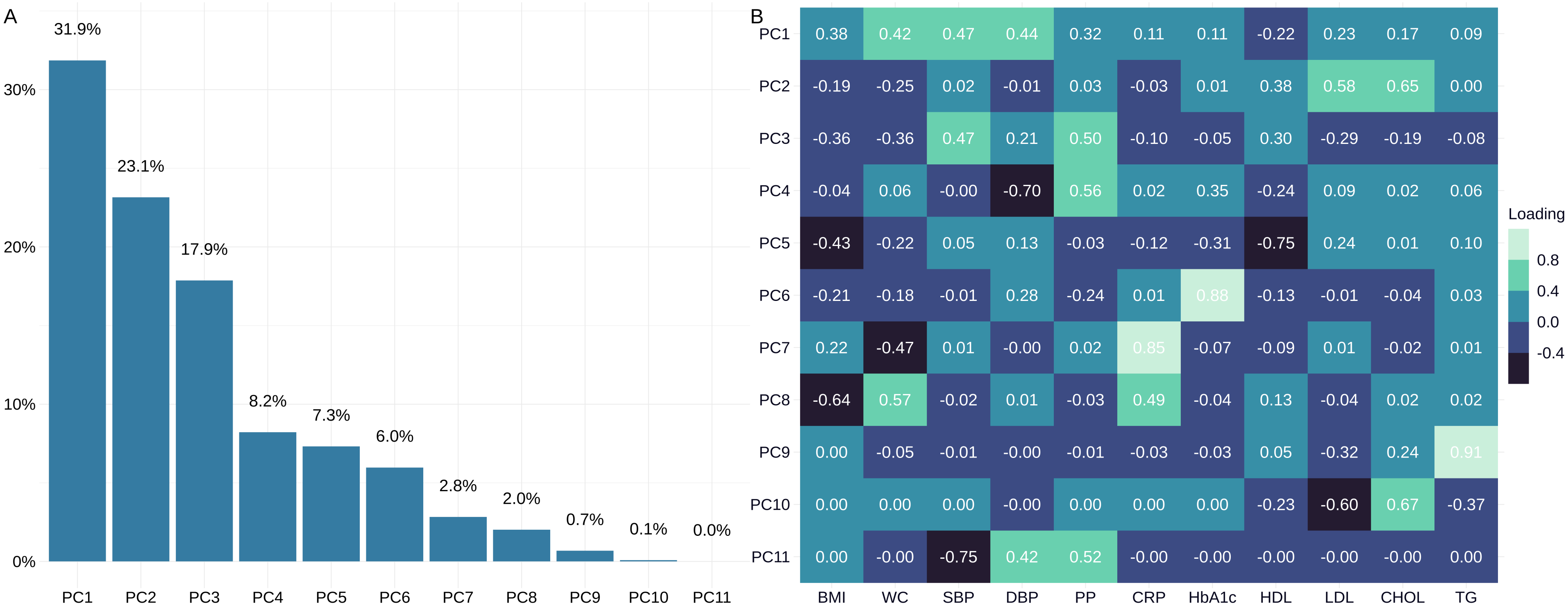

Notes: A) The explained variance of the 11 principal components from cardiometabolic principal component analysis without imputed data and B) their loadings from the 11 included cardiometabolic variables.
Abbreviations: PC, Principal component; BMI, Body mass index; WC, Waist circumference; SBP, Systolic blood pressure; DBP, Diastolic blood pressure; PP, Pulse pressure; CRP, C-reactive protein; HbA1c, Glycated hemoglobin; HDL, High-density lipoprotein cholesterol; LDL, Low-density lipoprotein cholesterol; CHOL, Total cholesterol; TG, Triglycerides

Figure S7: Correlations of the cognitive test variables

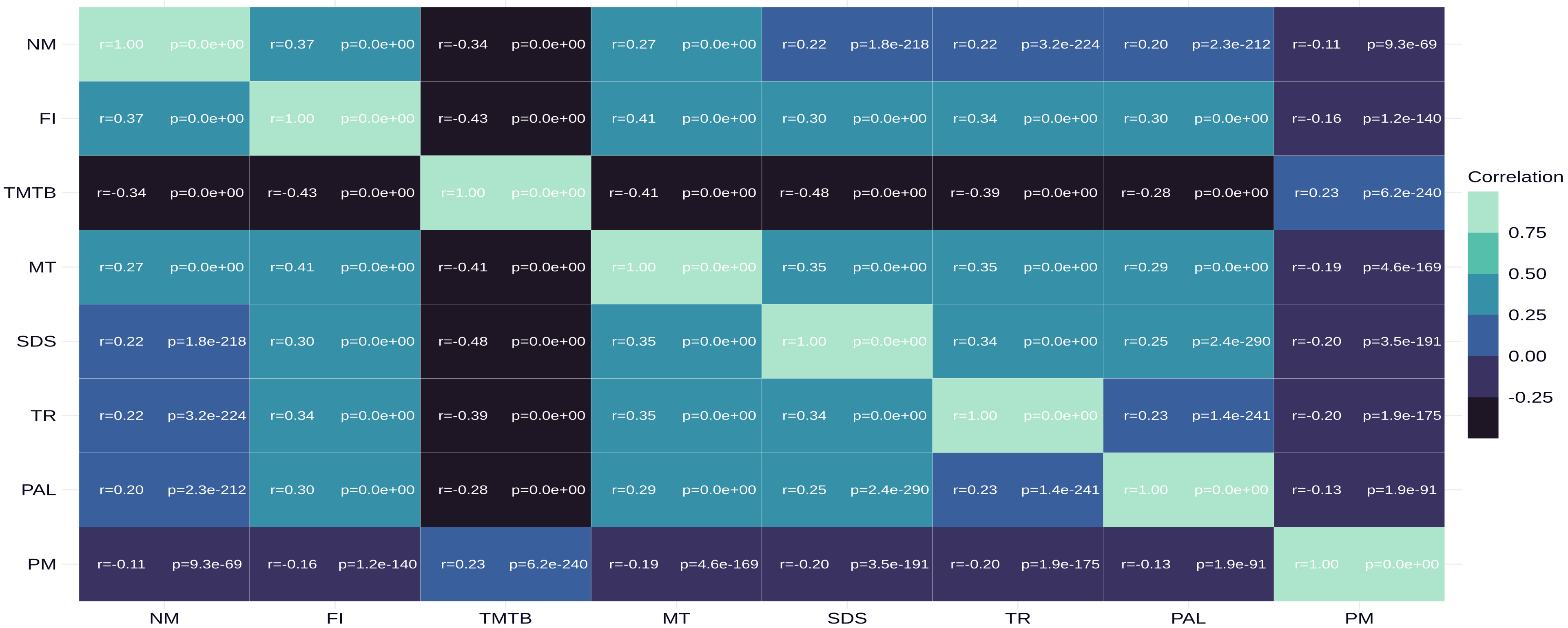

Notes: Pearson correlation matrix of the 8 included cognitive tests

Abbreviations: r, Partial correlation coefficient; p, P-value; NM, Numeric memory; FI, Fluid intelligence; TMTB, Trail making test B; MT, Matrix test; SDS, Symbol digit substitution; TR, Tower rearranging; PAL, Paired associate learning; PM, Pairs matching

Figure S8: Histograms of the cognitive test variables

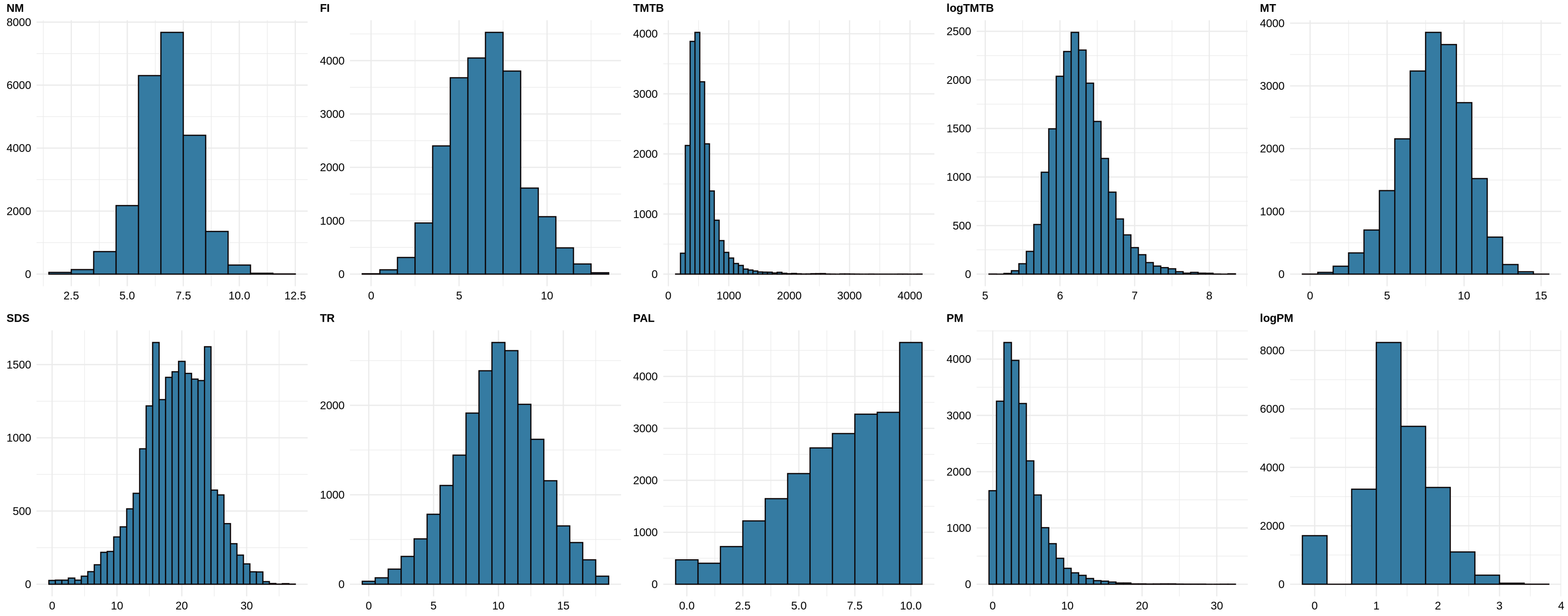

Notes: Histograms of the cognitive test variables.

Abbreviations: NM, Numeric memory; FI, Fluid intelligence; TMTB, Trail making test B; MT, Matrix test; SDS, Symbol digit substitution; TR, Tower rearranging; PAL, Paired associate learning; PM, Pairs matching

Figure S9: Quantile-quantile plots of the cognitive test variables

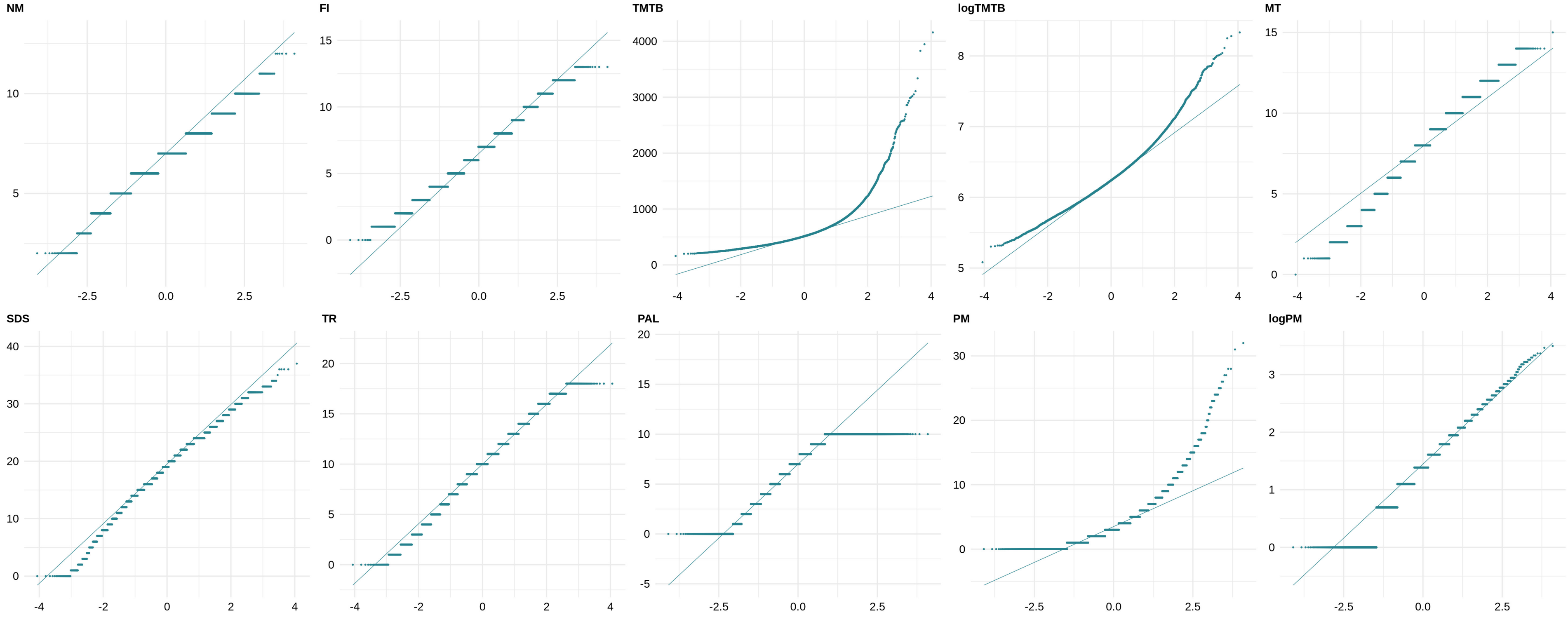

Notes: Quantile-quantile-plots of the cognitive test variables.

Abbreviations: NM, Numeric memory; FI, Fluid intelligence; TMTB, Trail making test B; MT, Matrix test; SDS, Symbol digit substitution; TR, Tower rearranging; PAL, Paired associate learning; PM, Pairs matching

Figure S10: Cognitive principal component analysis

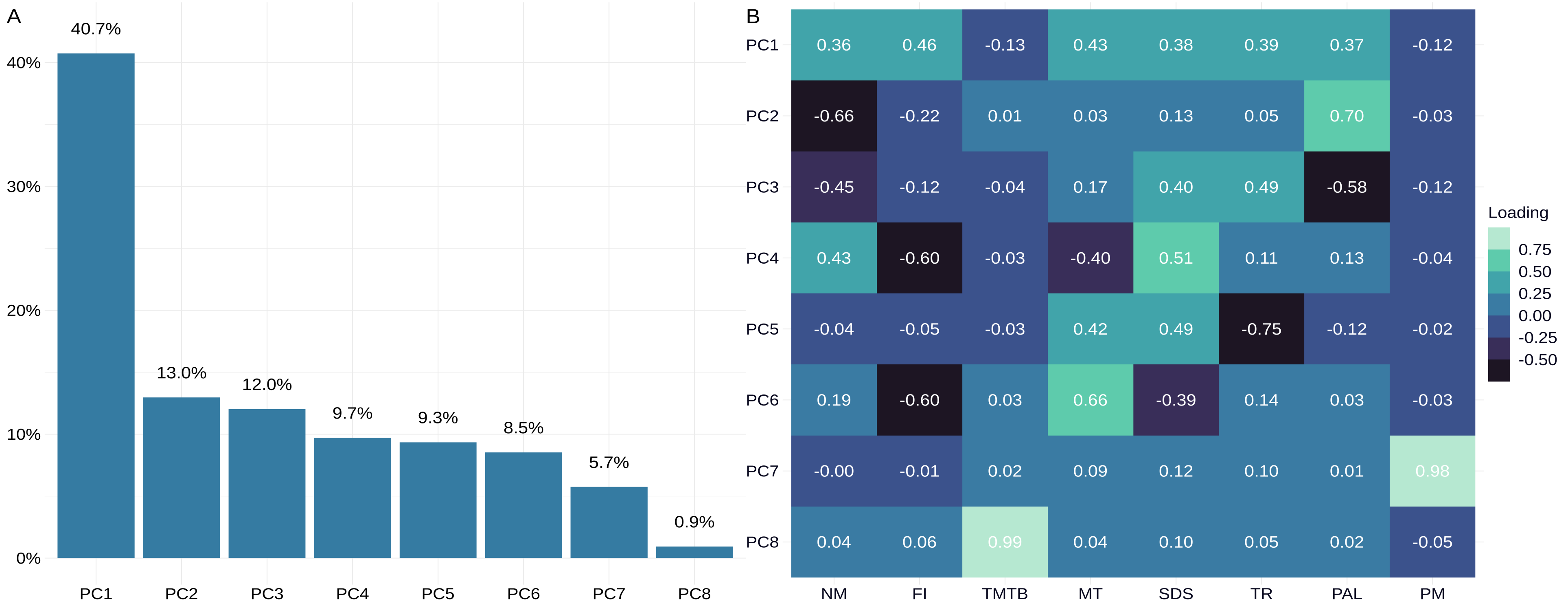

Notes: A) The explained variance of the 8 principal components from the cognitive principal component analysis and B) the components' loadings from the 8 included cognitive tests
Abbreviations: PC, Principal component; NM, Numeric memory; FI, Fluid intelligence; TMTB, Trail making test B; MT, Matrix test; SDS, Symbol digit substitution; TR, Tower rearranging; PAL, Paired associate learning; PM, Pairs matching

Figure S11: Cognitive principal component analysis without imputed data

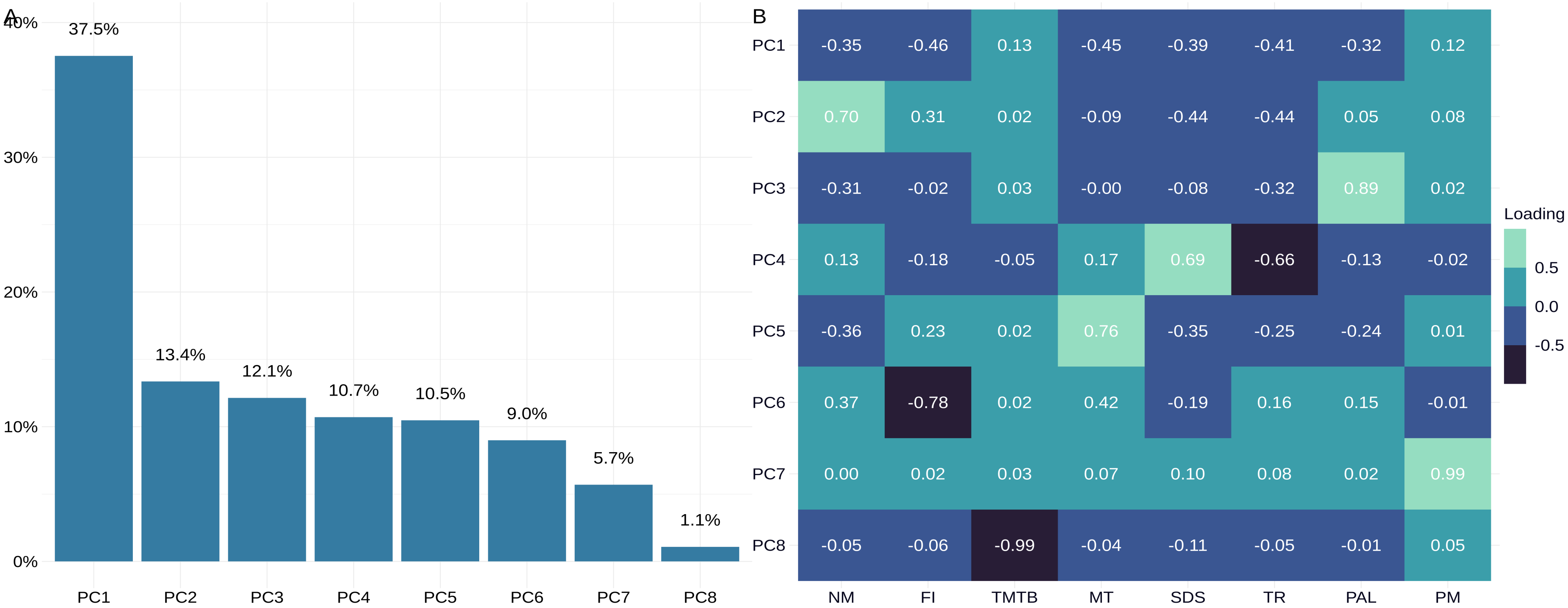

Notes: A) The explained variance of the 8 principal components from the cognitive principal component analysis without imputed data and B) the components' loadings from the 8 included cognitive tests
Abbreviations: PC, Principal component; NM, Numeric memory; FI, Fluid intelligence; TMTB, Trail making test B; MT, Matrix test; SDS, Symbol digit substitution; TR, Tower rearranging; PAL, Paired associate learning; PM, Pairs matching
